# Acute rapamycin treatment reveals distinct mechanisms of dysfunction in a maternal inflammation mouse model

**DOI:** 10.1038/s41467-026-74958-1

**Published:** 2026-07-23

**Authors:** JE Le Belle, M. C. Condro, C. Cepeda, KD Oikonomou, K. Tessema, L. Dudley, J. Schoenfield, R. Kawaguchi, D. Geschwind, AJ Silva, Z. Zhang, K. Shokat, NG Harris, HI Kornblum

**Affiliations:** 1 Semel Institute for Neuroscience & Human Behavior, Department of Psychiatry, David Geffen School of Medicine, UCLA, Los Angeles, CA USA; 2 The UCLA Brain Injury Research Center, Department of Neurosurgery, David Geffen School of Medicine, UCLA, Los Angeles, CA USA; 3 UCLA Intellectual and Developmental Disabilities Research Center, Semel Institute, Department of Psychiatry, David Geffen School of Medicine, UCLA, Los Angeles, CA USA; 4 Department of Psychiatry and Biobehavioral Sciences, David Geffen School of Medicine, UCLA, Los Angeles, CA USA; 5 Program in Neurogenetics, Department of Neurology, David Geffen School of Medicine, UCLA, Los Angeles, CA USA; 6 Department of Human Genetics, UCLA, Los Angeles, CA USA; 7 Brain Research Institute, UCLA, Los Angeles, CA USA; 8 Department of Neurobiology, UCLA, Los Angeles, CA USA; 9 Department of Psychology, UCLA, Los Angeles, CA USA; 10 Integrative Center for Learning and Memory, UCLA, Los Angeles, CA USA; 11 Department of Cellular and Molecular Pharmacology, Howard Hughes Medical Institute, University of California, San Francisco, CA USA; 12 Eli and Edythe Broad Center of Regenerative Medicine and Stem Cell Research, David Geffen School of Medicine at UCLA, Los Angeles, CA USA; 13 Jonsson Comprehensive Cancer Center, David Geffen School of Medicine at UCLA, Los Angeles, CA USA

**Keywords:** Autism spectrum disorders, Autism spectrum disorders

## Abstract

Maternal inflammatory response (MIR) during early mouse gestation induces a cascade of physiological and behavioral changes associated with autism spectrum disorder (ASD). We have shown that mild MIR causes chronic systemic and brain inflammation, mTOR pathway activation, mild brain overgrowth with regionally specific volumetric changes, sensory processing dysregulation, and repetitive behavior abnormalities. Prior rapamycin studies in autism models focused on chronic treatments that alter or prevent physical brain changes. Here, we focus on acute rapamycin effects to uncover novel mTOR pathway-mediated mechanisms of dysfunction. Within 2 hours, rapamycin rescues neuronal hyperexcitability, seizure susceptibility, functional network connectivity, brain community structure, repetitive behaviors, and sensory over-responsivity in adult MIR offspring. These CNS-mediated effects coincide with altered expression of genes associated with ASD, ion channels, and epilepsy. Our findings demonstrate that mTOR dysregulation drives dysfunctional brain development in MIR offspring but the adult brain remains amenable to rapid functional normalization, rescuing core and comorbid ASD-associated brain and behavior phenotypes. Restoring excitatory/inhibitory imbalance and sensory functional network modularity may be important targets for therapeutically addressing multiple ASD phenotypes.

## Abstract (web summary)

Here authors show acute rapamycin transiently rescues neuron hyperexcitability, network connectivity, repetitive behaviors, and sensory over-responsivity in an acquired model of autism, showing mTOR-driven phenotypes can be treatment responsive in the mature brain.

## Introduction

Neurodevelopmental disorders result from the disruption of brain development in utero or in early life^1^. Genetic, environmental, epigenetic, and immunological factors can all contribute to their complex pathogenesis^1–3^. For example, there are numerous different anatomical abnormalities in brain structure and cellular changes that have been observed in the brains of autistic individuals and in animal models of autism that indicate a multifactorial pathophysiology^4^. Despite this complex etiology and the spectrum of phenotypes in autism spectrum disorders (ASD), dysregulated mTOR pathway signaling in the brain is a common pathogenic mechanism^5–7^.

Abnormal PI3K/Akt/mTOR signaling pathway activity in response to divergent underlying causes can lead to aberrant brain structure, synapse formation, synaptic pruning, excitatory/inhibitory balance, neuro-inflammatory signaling, and brain size^3,7–10^. Hyperactive mTOR signaling contributes to seizures, sensory sensitivity, intellectual disability, macrocephalic brain growth, and a number of ASD-associated behavioral abnormalities^2^. This is thought to be related to the role of this important pathway in controlling cell growth, survival, energy balance, proliferation, autophagy, and ion channel expression. The mTOR pathway can become hyper-activated by several known mutations in ASD-associated genes^11^. ASD behaviors, macrocephaly, and epilepsy have also been recapitulated in a variety of *PTEN* and *TSC* murine knockout models in different cellular populations, developmental timeframes, and quantity of gene deletion. Furthermore, a number of high-confidence autism risk genes in humans belong to mTOR signaling gene sets^12–14^.

However, genetic mutations are not the only cause of increased mTOR activation and brain overgrowth. Hyper-activated mTOR signaling can both induce and be induced by neuro-inflammation of varying etiology^15–17^. There is considerable evidence of an elevated inflammatory state in ASD, including pro-inflammatory cytokines found in the blood and CSF and increased activated microglia found in post-mortem brains^6,18–20^. There is also evidence of brain overgrowth in ASD that is not linked to any specific genetic variation. Some studies have demonstrated a limited period of increased, albeit not macrocephalic, brain growth early in development that has no known genetic or non-genetic etiology to date^21^. Maternal immune system activation is a good candidate mechanism for this phenomenon because it is also known to contribute to brain overgrowth and ASD in humans and in rodent models^22–26^. We have previously shown that exposure to a low-level maternal inflammatory response (MIR) induced by lipopolysaccharide early in gestation produces offspring with mild brain overgrowth, hyperactive mTOR signaling in the neurogenic SVZ niche, and abnormal ASD-related behaviors in mice^27^.

In addition to the core behavioral features of ASD, there are many common comorbid phenotypes, including sensory over-responsivity (SOR), epilepsy, disordered sleep, and others^28^. Rather than approaching treatment as a “cure” for ASD, a common approach for current pharmacological therapy is focused on treating these non-core symptoms with existing approved drugs. The rationale for treating each aberrant phenotype separately rather than tackling core symptomology is the complex and not fully understood etiology of ASD, the spectrum of phenotypes and severity, and the lack of a core molecular target that underlies multiple aberrant behaviors. However, mTOR signaling is potentially one such convergent target that may impact both core and non-core phenotypes. Rapamycin, an inhibitor of mTOR, is known to be immunosuppressive, anti-proliferative, and an inducer of autophagy. It has been used in pre-clinical models of *PTEN* and *TSC* gene mutations to rescue the epileptic phenotype and several ASD-related behaviors in young mice by successfully preventing the development of aberrant brain physiological changes^29–31^. For example, developmental inhibition of mTOR over several weeks prevents brain overgrowth, loss of inhibitory neurons, seizures, and abnormal synaptic connectivity^29,32–35^. Rapamycin has, perhaps surprisingly, also been shown to rescue several abnormal phenotypes in adult mice despite the persistence of physiological brain abnormalities associated with *PTEN* and TSC gene mutations and mTOR activation, including macrocephalic brain overgrowth^6,29–31^. To date, rapamycin analogs have been used clinically to treat patients with ASD and *PTEN* or *TSC* gene mutations for weeks to months with mixed results, improving some cognitive functions, reducing seizure frequency, and improving social behaviors in some studies but not in others^36–38^. Chronic rapamycin treatment has also been shown to rescue some phenotypes in idiopathic ASD mouse models with elevated mTOR signaling^39,40^.

**Fig. 1:**
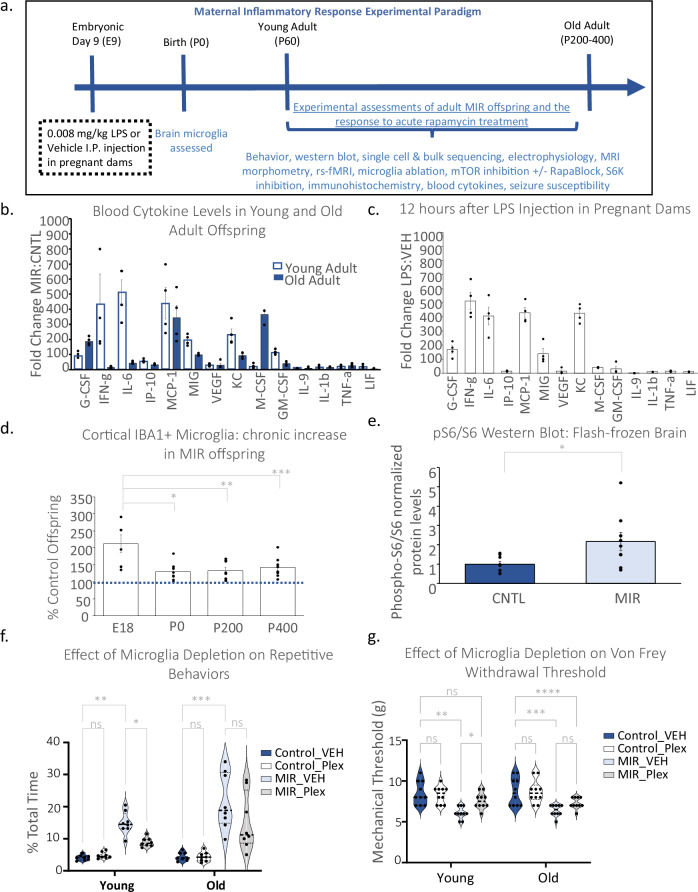
MIR mice have chronically elevated inflammatory state, mTOR pathway activation, repetitive behaviors, and sensory sensitivity that is reduced by microglia depletion in young but not old offspring. **a** Experimental timeline; **b** CNTL control, Y young, O old, inflammatory blood cytokines (shown as fold-change) significantly increased in Y and O MIR vs CNTL (two-way ANOVA with multiple comparisons (TWA-mc) cytokine x group effect F_(12, 44)_, *p* < 0.0001 and group effect F_(5, 18)_, *p* < 0.0001). Tukey’s (adjusted) (CNTL vs MIR) G-CSF (Y *p* = 0.0024, O *p* = 0.0048), IFN-g (Y *p* = 0.0137, O *p* = 0.0001), IL-6 (Y *p* = 0.0064, O *p* = 0.0015), IP-10 (Y non-significant (n.s.) *p* = 0.1132, O *p* = 0.0036), MCP-1 (Y *p* < 0.0001, O *p* = 0.0341), MIG (Y n.s., *p* = 0.2705, O *p* = 0.0419), VEGF (Y *p* = 0.0085, O *p* < 0.0001), KC (Y *p* = 0.0184, O *p* = 0.0027), M-CSF (Y *p* < 0.0001, O *p* = 0.0006), GM-CSF (Y *p* = 0.0044, O n.s., *p* = 0.0505), IL-9 (Y *p* = 0.0107, O *p* = 0.0009), IL-1b (Y *p* = 0.0003, O *p* = 0.0062), TNF-a (Y *p* = 0.0006, O *p* = 0.0036), LIF (Y *p* = 0.0009, O n.s., *p* = 0.7465), *n* = 4/group; **c** Pregnant dam blood cytokine changes VEH vs LPS (TWA-mc cytokine x group effect F_(12, 44)_, *p* < 0.0001 and group effect F_(5, 18)_, *p* < 0.0001). G-CSF (*p* < 0.0001), IFN-g (*p* = 0.0001), IL-6 (*p* < 0.0012), IP-10 (*p* < 0.0001), MCP-1 (n.s., *p* = 0.2705), MIG (*p* = 0.0016), VEGF (p = 0.0021), KC (*p* = 0.0197), M-CSF (*p* = 0.0011), GM-CSF (*p* < 0.0001), IL-9 (*p* = 0.0267), IL-1b (*p* = 0.0022), TNF-a (*p* = 0.0002), LIF (*p* = 0.0140)), *N* = 4/group; **d** IBA+ microglia in sensory-motor cortex MIR as % control, OWA-mc *p* < 0.0001, F_(3, 26)._ Tukey’s E18 v P0 (*p < 0.00001), E18 v P200 (**p < 0.0001), E18 v P400 (***p < 0.0001) and n.s. differences for P0 v P200 (*p* = 0.9975), P0 v P400 (*p* = 0.8248) and P200 v P400 (p = 0.9068), *N* = 8/group; **e** Phospho-S6 western blot on cortex, amygdala, and striatum (combined), Paired *t* test (two-tailed) significant difference CNTL vs MIR, *p = 0.0220, df = 8, *n* = 3/group/brain region; **f** TWA-mc significant group (*p* < 0.0001, F_(3,28)_) and age (*p* = 0.0140, F_(1, 28)_) effects in repetitive behaviors, Tukey’s Y-CNTL vs MIR (**p < 0.0001) and O-CNTL vs MIR (***p = 0.0001) for VEH diet and decreased in MIR VEH diet vs MIR Plexxikon 5622 (Plex) diet (Y *p = 0.0301) but not (O *p* = 0.0590) mice, *N* = 8/group (**g**) TWA-mc group effect (*p* < 0.0001, F_(3, 36)_) in Von Frey limb withdrawal threshold (LWT) in controls and MIR after Plex or VEH diets, Tukey’s lower LWT for MIR vs controls in (Y **p < 0.0001) and (O ***p = 0.0002) on VEH diet and increased LWT for MIR VEH vs MIR Plex diet in (Y *p = 0.0232) but not (O *p* = 0.3031); *n* = 10/group. All data Mean +/- SEM.

While there has been encouraging progress at rescuing mTOR-mediated brain pathology and behavioral abnormalities in pre-clinical models with chronic rapamycin treatments, there could still be risks to normal brain development with juvenile treatment in humans without specific mTOR pathway-related gene mutations, including adverse side effects from the use of such a potent growth inhibitor and immunosuppressant. We wanted to understand the mechanisms that underlie the effects of adult mTOR inhibition, where treatment isn’t aimed at preventing or reversing structural brain abnormalities. Here, we focus on the effects of mTOR inhibition within an acute timeframe, revealing new mechanisms and functional changes that are different from those reported with chronic treatment (Fig. 1a). We find that within two hours following rapamycin treatment of adult mice that had been exposed to mild maternal inflammation in utero results in significant normalization of deficits in behavior, sensory processing, brain functional connectivity and modularity, and electrophysiological hyperactivation. Furthermore, acute rapamycin affects specific cellular compartments and expression of ASD-, epilepsy- and ion channel-associated genes. Thus, with these findings, we have identified several potentially less disruptive targets for pharmacologic or neuromodulatory intervention, like systemic inflammation, sensory brain circuitry, functional network modularity, and neuronal hyper-excitability.

## Results

### Chronic pathophysiology in MIR offspring

**Fig. 2:**
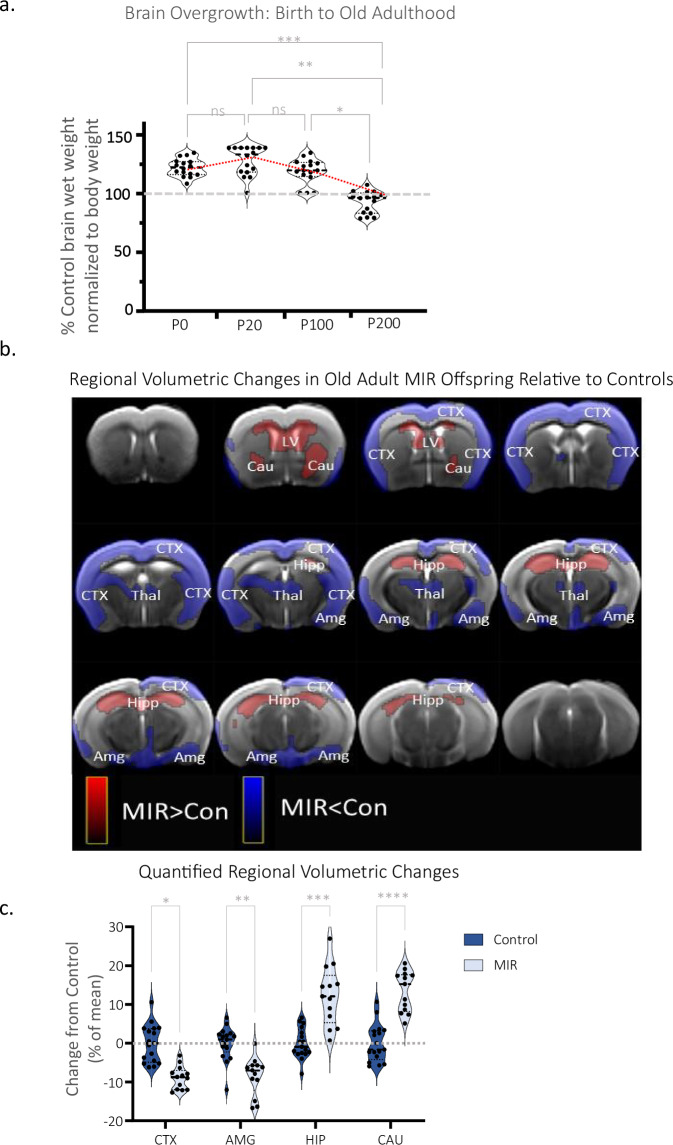
MIR offspring demonstrate growth patterns consistent with reports in humans with ASD of early brain overgrowth followed by undergrowth with cortical thinning and enlargement in other subcortical structures in adulthood. **a** Brain wet weights normalized to body weight of MIR offspring relative to control offspring from birth to post-natal day 200 (P200) show early overgrowth with most MIR brains being larger than average control brain (gray 100% line). A one-way ANOVA with multiple comparisons was overall significantly different between brain ages (*p* < 0.0001, F_(3, 60)_) with a significant (Tukey’s) decrease in brain size at P200 compared to P0 (***p < 0.0001), P20 (**p < 0.0001), and P100 (*p < 0.0001) but no significant size differences (ns) between P0 and P20 (*p* = 0.3602) or P20 and P100 (*p* = 0.0620), ages where brain overgrowth was maintained, *n* = 16/group. **b** Diffusion weighted MRI regional volumetric brain analysis of adult offspring (P400) showing mean percent differences between MIR and Control mice (blue = decreased red = increased brain volume) and variable size changes based on brain region with significant (*p* < 0.0001) decreased cortical thickness (CTX), thalamus (Thal) and amygdala (Amg) volumes but significant (*p* < 0.05) increases in lateral ventricle (LV), caudate (Cau), and hippocampus (Hipp) size. **c** Quantification of these regional deformation changes were significant overall by brain region and group in a two-way ANOVA with multiple comparisons (brain region x group, *p* < 0.0001, F_(2, 66)_, brain region, *p* < 0.0001, F_(2, 66)_, group, *p* = 0.0106, F_(1,28)_) with significant decrease in volume in MIR offspring compared to controls post-hoc tests (Tukey’s) in cortex (CTX, *p < 0.0001, DF = 27), amygdala (AMG, **p < 0.0001, DF = 24) and significant increases in volume in MIR compared to control in the hippocampus (HIP, ***p < 0.0001, DF = 17) and caudate (CAU, ****p < 0.0001, DF = 25), *n* = 17/group.

**Fig. 3:**
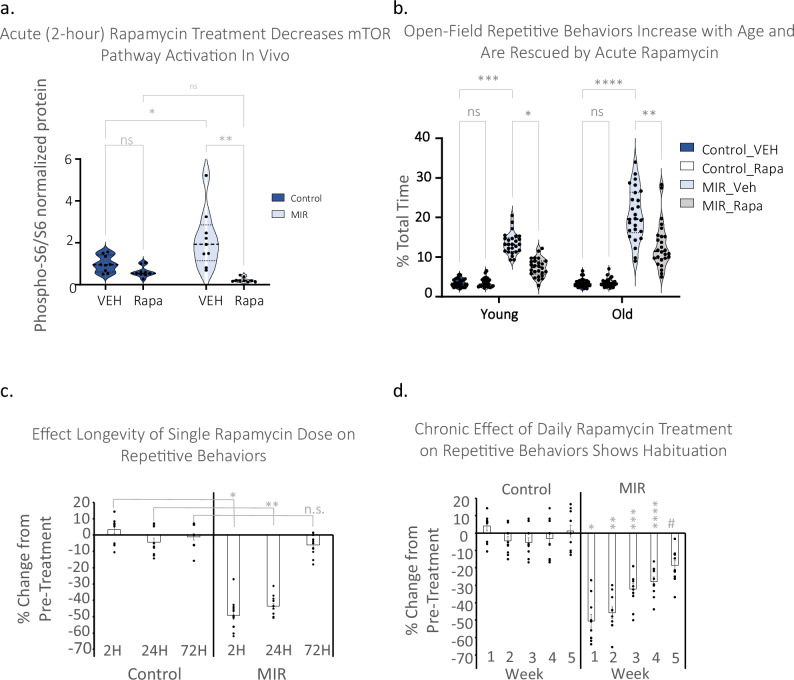
Acute rapamycin treatment rescues elevated brain mTOR signaling and repetitive and social behavior abnormalities in MIR offspring. **a** Two-way ANOVA analysis for multiple comparisons (TWA-mc) of western blot data from pooled cortex, striatum, and amygdala tissue showing significant differences in treatment x group (*p* < 0.0053, F_(1, 16)_) and treatment (*p* = 0.0002, F_(1,16)_). Post-hoc (Tukey’s) analysis shows a significant increase in phospho-S6/S6 in MIR vs controls treated with vehicle (VEH; *p = 0.0020, DF = 32) and a significant reduction in MIR vs controls treated with rapamycin compared to VEH (**p < 0.0001, DF = 16), demonstrating chronically upregulated mTOR pathway activation and rapamycin gets into the brain and reduces that activation within 2 hours, *n* = 3/group/brain region; **b** TWA-mc of repetitive behaviors shows an overall significant difference in age x group (*p* < 0.0001, F_(3,100)_), age (*p* < 0.0001, F_(1, 100)_), and group (*p* < 0.0001, F_(3, 100)_). Tukey’s shows significant increase in repetitive behaviors in MIR vs controls treated with vehicle in young (***p < 0.0001, DF = 200) and old (****p < 0.0001, DF = 200) mice and a significant decrease in behaviors in MIR treated with rapamycin vs MIR treated with vehicle in young (*p < 0.0001, DF = 200) and old (**p < 0.0001, DF = 200) offspring, *n* = 26/group. **c** TWA-mc of open field repetitive behaviors over time after single rapamycin treatment indicate significant time x group (*p* < 0.0001, F_(2, 39)_), time (*p* < 0.0001, F_(2, 39)_), and group (*p* < 0.0001, F_(1,22)_) effects. Tukey’s shows significant difference in Control vs MIR offspring at 2 hours (2H, *p < 0.0001, DF = 22) and 24 hours (24H, **p < 0.0001, DF = 20) but not 72 hours (72H, *p* = 0.0738, DF = 22); *n* = 12/group; **d** TWA-mc of open field repetitive behavior after repeat daily doses of rapamycin show time x group (*p* < 0.0001, F_(3, 48)_), time (*p* < 0.0001, F_(3, 48)_), and group (*p* < 0.0001, F_(1, 18)_) effects. Tukey’s indicates significant treatment effect in MIR vs controls with daily rapamycin at 1 week of daily treatment (W1, *p < 0.0001, DF = 16), week 2 (W2, **p < 0.0001, DF = 16), week 3 (W3, ***p < 0.0001, DF = 17), week 4 (W4, ****p < 0.0001, DF = 17), and week 5 (W5, #*p* = 0.0006, DF = 18) although the difference gets smaller each week, indicating gradual loss of efficacy with chronic administration. *N* = 10/group; All data shown as mean ±SEM.

**Fig. 4:**
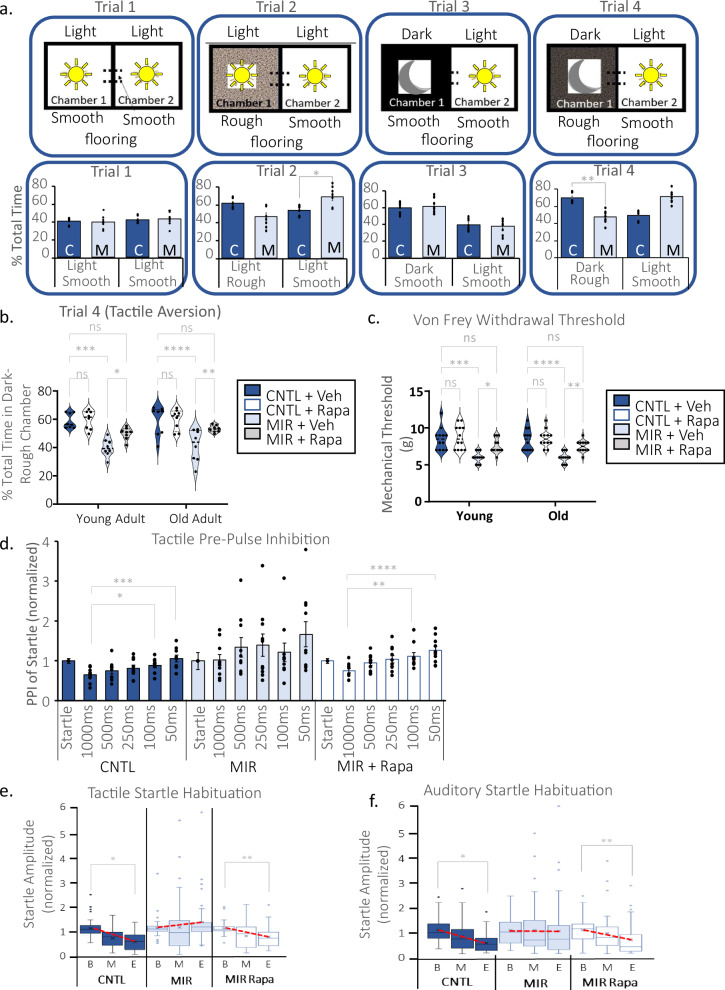
MIR mice display significant sensory over-responsivity (SOR) and rescue with rapamycin. **a** MIR mice have: normal exploratory behavior in trial 1 and 2, normal time spent in a dark chamber with smooth floors (Trial 3), but less time in the dark chamber when flooring is rough (Trial 4). Percent total time spent in chamber by MIR vs controls with two-tailed paired t-test shows no significant difference in trial 1 (*p* = 0.7074, DF = 7) and trial 3 (*p* = 0.5272, DF = 7), but significant difference in trial 2 (*p = 0.0083, DF = 7) and trial 4 (**p = 0.0002, DF = 7), *n* = 8/group. all data mean ± SEM. **b** A two-way ANOVA analysis for multiple comparisons (TWA-mc) of trial 4 found a significant group effect (*p* < 0.0001, F_(3, 28)_). Post-hoc (Tukey’s) shows MIR vs controls spend significantly less time in dark-rough chamber in both young (*p* < 0.0001, DF = 56) and old (*p* < 0.0001, DF = 56), and rapamycin increases this time in young (*p* = 0.0380, DF = 56) and old (*p* = 0.0102, DF = 56), *n* = 8/group. **c** TWA-mc of Von Frey limb withdrawal threshold (LWT) found a significant effect of group (*p* < 0.0001, F_(3,44)_). Tukey’s shows MIR mice have a significantly lower LWT than controls in young (***p < 0.0001, DF = 88) and old (****p < 0.0001, DF = 88). Rapamycin significantly increased LWT in MIR vs vehicle-treated MIR in young (*p = 0.0073, DF = 88) and old (**p = 0.0123, DF = 88), *n* = 12/group. **d** TWA-mc of startle inhibition shows significant interval (*p* = 0.0015, F_(2, 65)_) and group (*p* = 0.0132, F_(2, 27)_) differences. Tukey’s shows MIR mice treated with rapamycin show significant changes in startle response at 1000 ms vs 100 ms prepulse (*p = 0.0041, DF = 9 and MIR+Rapa *p = 0.0211, DF = 9) and at 1000 ms vs 50 ms (***p = 0.0042, DF = 9 and MIR+Rapa ****p = 0.0053, DF = 9), *n* = 10/group, all data mean ± SEM; **e** TWA-mc of startle response to tactile (airpuff) startle at beginning (B), middle (M), and end (E) of PPI test found significant time x group (*p* = < 0.0001, F_(4, 268)_), time (*p* = 0.0003, F_(2, 268)_), and group (*p* < 0.0001, F_(2, 147)_) effects. Tukey’s shows significant decreases in airpuff startle response at beginning vs end for control mice (*p < 0.0001, DF = 49) and MIR mice treated with rapamycin (*p < 0.0001, DF = 49) but no significant difference for MIR mice treated with vehicle (*p* = 0.1835, DF = 49), *n* = 10/group; **f** TWA-mc for audible startle found significant effects of time (*p* = 0.0005, F_(4, 288)_) and group (*p* = 0.0145, F_(2, 288)_). Tukey’s shows significant startle decreases in control (*p* = 0 < 0.0001, DF = 49) and MIR mice treated with rapamycin (*p* = 0.0011, DF = 49) but no significant difference in MIR mice treated with vehicle (*p* = 0.9757, DF = 49), *n* = 10/group. Box and whisker (**e**, **f**) bars represents the 0th and 100th percentile, bottom edge of box is 25th percentile, top edge of box is 75th percentile, median line in box is 50th percentile.

Building on our previous finding of mild brain overgrowth and increased PI3K/AKT/mTOR signaling in the juvenile and young adult SVZ niche^27^, we show that MIR-induced pathophysiology persists and evolves throughout adulthood, including, chronically elevated blood cytokines (Fig. 1b, c), increased brain microglia (Fig. 1d), elevated brain phospho-S6 indicating chronic mTOR activation (Fig. 1e), reduced reciprocal social interactions (Supplementary Fig. 3b), increased repetitive behaviors (Figs. 1f and 3c, Supplementary Figs. 1a, c, d and  3b), altered gross brain weight and regional volumes (Fig. 2a–c), and abnormal sensory responses across tactile avoidance, Von Frey, and pre-pulse inhibition (PPI) tests (Figs. 1g and 4a–f).

Blood cytokine analysis in pregnant dams (Fig. 1c) and young and old adult MIR offspring revealed an ongoing pro-inflammatory state with large fold-increases in G-CSF, IFN-γ, IL-6, MCP-1, MIG, KC, M-CSF, and GM-CSF, and smaller but significant increases in IL-9, IL-1β, TNF-α, VEGF, IP-10, and LIF (Fig. 1b). The largest increases were in IFN-γ, MCP-1, and IL-6 in young adults and M-CSF, MCP-1, and G-CSF in old adults. Acute 2-hour rapamycin had a negligible effect on these cytokines (Supplementary Fig. 3a), indicating its behavioral effects are not driven by directly reducing systemic inflammation, though they may mitigate its downstream consequences. IBA1+ immunostaining confirmed elevated microglia from before birth through old age (Fig. 1d), and pooled western blots from sensorimotor cortex, striatum, and amygdala confirmed elevated mTOR activation (Fig. 1e).

To test whether brain microglia drive these phenotypes, we depleted microglia with the CSF1R inhibitor Plexxikon5622. Microglia ablation partially rescued repetitive, social, and sensory abnormalities in young adult MIR mice but not in older adults, despite rapamycin remaining effective at the older age (Fig. 1f, g). Thus, microglia can contribute to the development of these behaviors but are unlikely to be the primary mediator of ongoing dysfunction.

The MIR model also recapitulates the brain growth trajectory reported in 50–70% of autistic humans^41^, with mild overgrowth at young ages followed by reduced growth at older adult ages via brain weight (Fig. 2a) and altered regional brain volumes by MRI analysis (Fig. 2b, c). Adult MIR mice show increased hippocampus, caudate, and amygdala volumes but decreased thalamus and sensory, motor, and association cortices, consistent with reported human ASD volumetric findings^42,43^.

### Acute rapamycin treatment effects on ASD-related behaviors

Given the persistently elevated mTOR activation in MIR offspring, we tested if acute rapamycin treatment could alter physiology and behavior without requiring structural brain changes like longer-term axonal and synaptic remodeling. Phospho-S6 western blots from pooled cortex, striatum, and amygdala confirmed BBB penetrance and brain mTOR inhibition within 2 hours (Fig. 3a), rationalizing an examination of the effect on multiple ASD-associated behaviors within that timeframe. We observed significant improvements in reciprocal social interactions (Supplementary Fig. 3b) and repetitive behaviors (grooming and circling in an open field) in both young and old adult mice (Fig. 3b). We observed high levels of repetitive behaviors in MIR offspring that become more severe in old MIR offspring (Fig. 3b), and acute rapamycin markedly reduces repetitive behavior and social deficits at both young and old ages (Fig. 3b, Supplementary Fig. 3b). A single dose of rapamycin has a temporary rescue effect on repetitive behaviors with significant behavioral abnormalities returning after 72 hours in MIR offspring (Fig. 3c). However, repeated daily treatment with rapamycin leads to an apparent tolerance effect over 5 weeks leading to a loss of effectiveness on repetitive behaviors (Fig. 3d).

MIR offspring also displayed SOR, a common ASD phenotype^44^. Compared to controls, MIR mice showed increased aversion to rough tactile textures in a novel light/dark avoidance test (Fig. 4a, b), reduced Von Frey thresholds for limb withdrawal (Fig. 4c), and abnormal sensory gating with absent startle habituation in PPI using tactile (air puff) pre-pulses and auditory and tactile startles (Fig. 4d–f). All sensory abnormalities were at least partially rescued by acute rapamycin in both young and old adult mice (Fig. 4b–f).

### Central nervous system actions of rapamycin

Because peripheral neuron abnormalities can mediate sensory phenotypes in ASD models^45^, we tested RapaBlock, a small molecule that blocks rapamycin’s peripheral effects without crossing the BBB^46^. RapaBlock did not change rapamycin’s rescue of repetitive behaviors, reciprocal social interactions, or sensory aversion (Supplementary Figs. 1a, b and  3b), suggesting that the main effect of acute rapamycin in MIR mice is on the central nervous system in MIR mice.

### mTOR pathway specificity of effects

To confirm mTOR pathway signaling specificity of behavioral rescue, we administered the S6K inhibitor PF4708671^47^ and observed partial rescue of repetitive behaviors at 6 hours (Supplementary Fig. 1c); a 2 hour treatment had no effect (data not shown), likely reflecting slower brain penetrance. Apocynin, an NADPH oxidase inhibitor that reduces PI3K/AKT/mTOR activity via redox regulation of PTEN^27,48^, had no acute effect but significantly reduced repetitive behaviors after 5 weeks of daily treatment (Supplementary Fig. 1d). In a direct comparison of treatment effects on repetitive behaviors, we show that rapamycin has the largest behavioral rescue effect compared to S6K inhibition, microglia depletion, or chronic apocynin treatments (Supplementary Fig. 1e).

### Neuronal hyper-excitability & seizure susceptibility

**Fig. 5:**
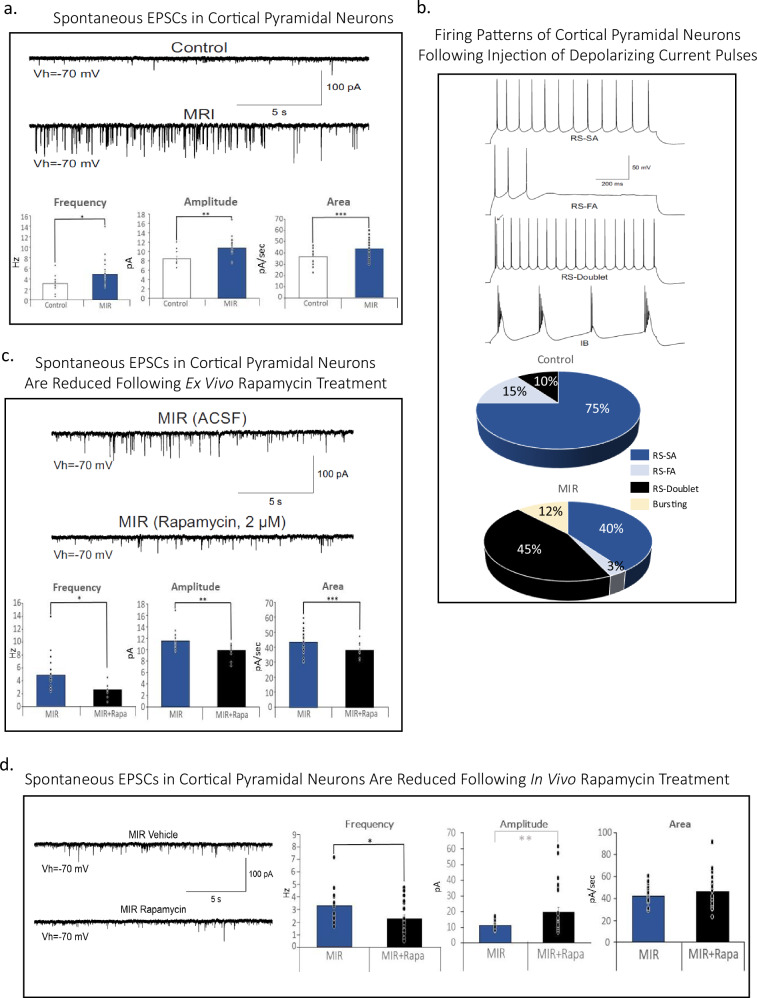
Cortical neurons in MIR offspring display abnormal electrophysiology indicating hyper-excitability which is rescued by acute rapamycin treatment. **a** A mixed effects analysis for multiple comparisons was performed on the quantification of the electrophysiological properties of spontaneous excitatory postsynaptic currents (EPSCs) in cortical pyramidal neurons and shows an overall significant effect by variable for fixed effects (*p* < 0.0001, F_(1, 36)_) and group (*p* = 0.0049, F_(1, 33)_). Post-hoc analysis (Sidak’s multiple comparisons) shows a significant difference between MIR and control mice for frequency (adjusted *p = 0.0160, DF = 29), amplitude (adjusted **p = 0.0022, DF = 24), and area (***p = 0.0312, DF = 21), *n* = 22/group. **b** Firing patterns of cortical pyramidal neurons following injection of depolarizing current pulses (500–1000 ms) show that layer 2/3 neurons from MIR mice have decreases in regular spiking, slow, and fast adaptation neuron firing, increased doublet firing, and ectopic burst firing that is not present in the neurons from control mice. **c** A mixed effects analysis for multiple comparisons was performed on the quantification of the electrophysiological properties of spontaneous excitatory postsynaptic currents (EPSCs) in cortical pyramidal neurons and shows an overall significant effect by variable (*p* < 0.0001, F_(1, 37)_) and group (*p* = 0.0028, F_(1, 32)_. Post-hoc analysis (Tukey’s) shows a significant rescue effect on MIR neurons treated by ex vivo rapamycin (2 μM) in slice culture compared to MIR neurons treated with vehicle during electrophysiology recording in frequency (adjusted *p = 0.0013, DF = 31), amplitude (adjusted **p = 0.0035, DF = 29), and area (adjusted ***p = 0.0167, DF = 30), *n* = 24/group. **d** A mixed effects analysis for multiple comparisons was performed on the quantification of the electrophysiological properties of spontaneous excitatory postsynaptic currents (EPSCs) in cortical pyramidal neurons and shows an overall significant effect by variable (*p* < 0.0001, F_(2, 65)_). Post-hoc analysis (Tukey’s) shows a significant rescue effect MIR mice treated by in vivo rapamycin (5 mg/kg 2 hours prior to brain slice preparation) compared to MIR mice treated with vehicle in frequency (adjusted *p = 0.0146, DF = 35), amplitude (adjusted **p = 0.0151, DF = 24), but not in area (adjusted *p* = 0.3925, DF = 26). The following abbreviations were used: Cm membrane capacitance, pF picofarads, Rm membrane resistance, MΩ ohms, Tau time constant, ms milliseconds, RMP resting membrane potential, mV millivolts, pA picoamperes. *n* = 24/group. All data mean ± SEM.

In order to understand potential physiological mechanisms underlying the behavioral changes in MIR offspring and the rapid effects of rapamycin, we examined the electrophysiological properties of neurons in brain slices. We found marked hyperexcitability in cortical layer 2/3 somatosensory pyramidal neurons (CPNs) in adult MIR offspring, indicated by reduced resting membrane potential, decreased Rheobase (Table 1), and significantly increased spontaneous Excitatory PostSynaptic Potential frequency, amplitude, and area (Fig. 5a) and voltage evoked responses (Supplementary Fig. 4a, b), indicating marked hyperexcitability. Under current clamp (depolarizing pulses of 500–1000 ms), control CPNs showed the typical regular spiking slowly adapting (RS-SA) pattern^49^ (75% RS-SA, 15% RS-FA, 10% RS-Doublet, 0% intrinsic bursting, Fig. 5b). MIR CPNs had a dramatically altered pattern (40% RS-SA, 3% RS-FA, 45% RS-Doublet, and 12% intrinsic bursting), which is not normally seen in upper layer neurons (Fig. 5b).

**Table 1:** Passive membrane properties of cortical pyramidal neurons

	Cm (pF)	RM (MΩ)	Tau (ms)	RMP (mV)	Rheobase (pA)
Control	208.7 ± 21	109.2 ± 16	4.0 ± 0.4	−78.1 ± 2.4	725.6 ± 83
MIR	207.8 ± 14	129.0 ± 16	3.6 ± 0.3	−70.6 ± 1.3*	499.2 ± 52**
MIR+Rapa	214.5 ± 8	106.9 ± 8	3.7 ± 0.17	−73.3 ± 1.4	647 ± 65

Passive membrane properties (membrane capacitance, input resistance, time constant, and resting membrane potential (RMP)), and Rheobase) of cortical pyramidal neurons show MIR mice have significantly higher resting membrane potential (*p < 0.05, closer to action potential threshold) and decreased Rheobase (**p < 0.05, lower minimum current to depolarize) indicating neuronal hyper-excitability in the cortex and increased membrane capacitance in the striatum compared to controls; all data mean ± SEM; *N* = 24/group/treatment; a two-way ANOVA analysis for multiple comparisons with post-hoc Tukey’s analysis of group effects was used.

In vitro rapamycin (2 µM in slice media) normalized resting membrane potential, increased Rheobase (Table 1), and rescued sEPSC frequency, amplitude, and area (Fig. 5c); in vivo treatment (5 mg/kg IP, 2 h prior) similarly reduced EPSC frequency (Fig. 5d). Striatal medium spiny neurons (MSNs) showed a nonsignificant trend toward increased sEPSC frequency that was reduced by acute rapamycin (Supplementary Fig. 4c, d). Blocking GABAA receptors with bicuculline (10 µM) ex vivo unmasked paroxysmal cortical discharges that propagated to the striatum, manifesting as large synaptic currents and bursts in MSNs that were rare in controls (Supplementary Fig. 4e); rapamycin also reduced this hyperexcitability induced by bicuculline (Supplementary Fig. 4f). MIR mice were also more susceptible to seizures induced by Pentylenetetrazole (PTZ), with all MIR mice (8/8) showing seizure activity at 40 mg/kg versus only 2/8 controls; rapamycin lowered seizure scores in MIR mice but did not abolish the effect of PTZ (Table 2, Supplementary Table 4). Together, these findings indicate significant neuronal hyperexcitability that is normalized by acute rapamycin, suggesting rapid restoration of excitatory and inhibitory balance.

**Table 2:** Seizure susceptibility by PTZ dosage

	PTZ dosage (mg/kg)									
	10	20	30	40	40 + Rapamycin					
Maximum seizure score	CNTL	MIR	CNTL	MIR	CNTL	MIR	CNTL	MIR	CNTL	MIR
0	**8/8**	**8/8**	**8/8**	**7/8**	**7/8**	**4/8**	**6/8**	**0/8**	**7/8**	**0/8**
1	0/8	0/8	0/8	**1/8**	**1/8**	**3/8**	**2/8**	**1/8**	**1/8**	**3/8**
2	0/8	0/8	0/8	0/8	0/8	**1/8**	**0/8**	**2/8**	**0/8**	**4/8**
3	0/8	0/8	0/8	0/8	0/8	0/8	0/8	**3/8**	0/8	**1/8**
4	0/8	0/8	0/8	0/8	0/8	0/8	0/8	**2/8**	0/8	0/8
5	0/8	0/8	0/8	0/8	0/8	0/8	0/8	0/8	0/8	0/8
6	0/8	0/8	0/8	0/8	0/8	0/8	0/8	0/8	0/8	0/8

Seizure scores during pentylenetetrazol (PTZ) dose escalation indicates a greater severity and larger number of mice per group displaying a seizure behavior in MIR mice compared to Control offspring that is decreased by acute rapamycin treatment. *N* = 8 mice/group, Bold text indicates there are mice in that seizure level and non-bold that there are no mice at that level.

### Circuit-level functional dysregulation of MIR brains is altered by acute rapamycin treatment

**Fig. 6:**
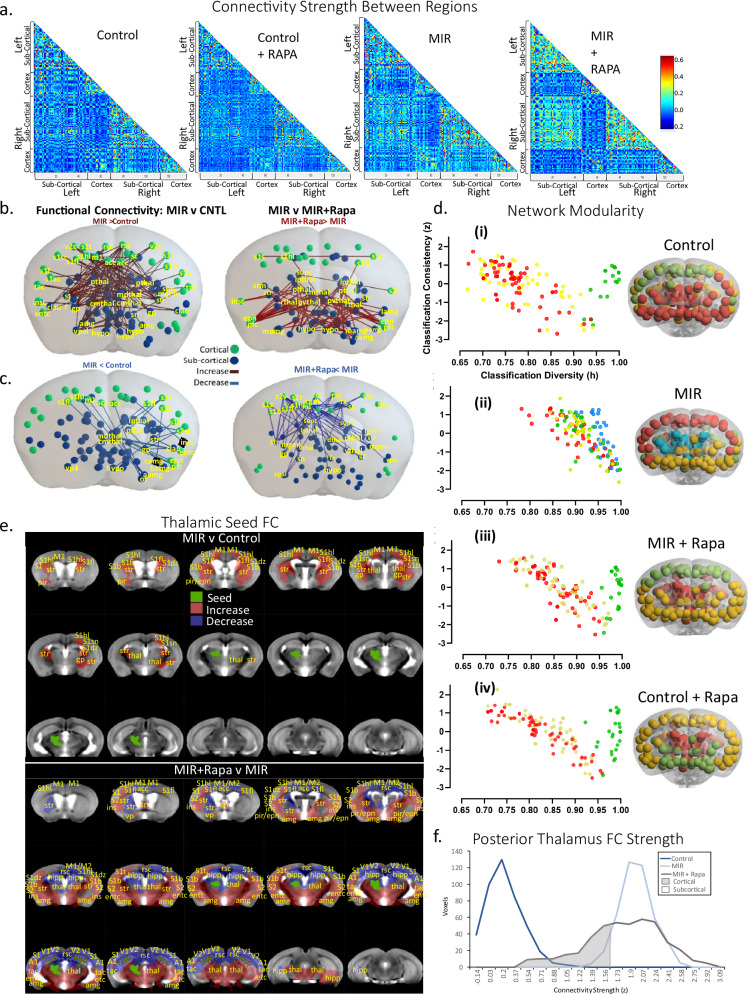
Disordered functional connectivity seen in MIR mice is significantly rescued by acute rapamycin treatment. **a** Fisher z scores (transformed *r* values, 0.2–0.6) indicating functional connectivity strength between cortical and subcortical brain regions in MIR and control mice before and after acute rapamycin treatment. **b** Regional increases in functional connectivity in resting state fMRI from MIR and control mice before and after acute rapamycin treatment (*P* < 0.01 NBS, 2-tailed). **c** Regional decreases in functional connectivity in resting state fMRI from MIR and control mice before and after acute rapamycin treatment (*P* < 0.01 NBS, two-tailed). **d** Differential network modularity organization across the brain is increased in MIR mice compared to control offspring and is rescued by acute rapamycin treatment. **e** Differential functional connectivity by brain region from a left posterior thalamus seed in MIR mice compared to control offspring before and after acute rapamycin treatment. **f** Voxel-wise quantification of posterior thalamus functional connectivity is significantly increased in MIR mice compared to control offspring and is differentially affected by acute rapamycin treatment with cortical regions showing decreased thalamic FC and subcortical regions showing increased thalamic FC following treatment; *N* = 16/group Abbreviations used as follows: **acc**=anterior cingulate cortex; **rsc**=retrosplenial cortex; **sc**=subicular complex; **v2c**=secondary visual cortex; **s1s**=primary somatosensory cortex; **s2s**=secondary somatosensory cortex; **s1b** = S1 barrel cortex; **s1a**=primary auditory cortex; **dthal**=dorsal thalamus; **vthal**=ventral thalamus; **pthal**=posterior thalamus; **pvthal**=paraventricular thalamus; **mdthal**=medial dorsal thalamus; **rthal**=reticular thalamus; **lpthal**=lateral posterior thalamus; **tac**=temporal association cortex; **stm**=stria terminalis; **mgen**=medial geniculate; **lgen**=lateral geniculate; **str**=striatum; **cmthal**=central median thalamus; **clau**=claustrum; **gp**=globus pallidus; **sn**=substantia nigra; **amg** =amygdala; **lamg**=lateral amygdala; **hypo**=hypothalamus; **vpal**=ventral pallidum; **entc**=entorhinal cortex; **insc**=insular cortex; **s1f** = primary face somatosensory cortex; **s1fl**=primary forelimb somatosensory cortex; **s1hl**=primary hindlimb somatosensory cortex; **s1t**=primary trunk somatosensory cortex; **m1**=primary motor cortex; **m2**=secondary motor cortex; **ot**=olfactory tubercles; **aamg**=anterior amygdala; **camg**=central amygdala; **bamg**=basal amygdala; **mamg** =medial amygdala; **pfc**=piriform cortex; **epn**=endopiriform nucleus; **s2c**=secondary somatosensory cortex; **supc**=superior colliculus; **fx**=hippocampal fornix; fim=hippocampal fimbria; **cgm**=cingulum; **hab**=habenular; **dg**=dentate gyrus; **ca3**=hippocampus CA3; **paqu**=periaqueductal gray; **rtf**=reticular formation; **lsep**=lateral septum.

A potential mechanism by which acute rapamycin treatment could rapidly affect brain function and behavior is through effects at the level of functional connectivity and network organization. Therefore, we performed resting state fMRI and found increased brain-wide functional connectivity (FC) in MIR mice compared to control offspring, especially within and between cortical and subcortical regions in each hemisphere (Fig. 6a). Network based statistical analysis (*P* < 0.01, two-tailed) revealed that altered FC in MIR mice was primarily due to increases between primary and higher-order (secondary) cortical sensory processing areas (somatosensory for hind limbs, forelimbs, whiskers, trunk, and neck/shoulder, visual, auditory, gustatory, and olfactory) and subcortical structures (thalamus and basal ganglia), but also some decreases were seen in FC between sensory cortical areas and amygdala (Fig. 6b, c)). Despite the strong repetitive behavior phenotype in MIR mice, motor cortex-related differences were more limited than sensory cortex, with primary motor cortex showing increased FC only to retrosplenial cortex, subicular complex, and striatum. The largest FC increases in MIR mice occurred between subcortical structures, primarily basal ganglia connections with thalamus and sensory cortex, while decreases were sparser and constrained to sensory cortex to thalamus and amygdala (Fig. 6b).

Acute rapamycin treatment then produced subcortical FC increases and cortical FC decreases in MIR mice with limited effect in controls (Fig. 6a–c, Supplementary Fig. 3c). Specific FC decreases occurred between somatosensory areas (primary somatosensory cortex for forelimb, hindlimb, trunk, barrel) and basal ganglia/thalamic structures, while increases occurred between left and right primary somatosensory cortex; from insular, piriform, and endo-piriform cortex to thalamic structures; and between amygdala and thalamic/basal ganglia structures (Fig. 6c).

Modularity analysis revealed four main modules in MIR brains (cortical, two subcortical/cortical, and thalamic-basal ganglia) with a modularity index of 0.693, versus only three modules in control offspring (anterior cortical, posterior cortical, mainly subcortical) and a significantly lower index of 0.511 (*p* < 0.01; Fig. 6d). Classification consistency and diversity (CC, CD) of brain regions showed that all MIR modules except the cortical modules had high CD relative to controls, indicating greater inter-module integration of connections. Acute rapamycin treatment normalized MIR architecture to three modules with index 0.552 (*P* < 0.05), shifting cortical CD up and subcortical CD down toward control values (Fig. 6dii–iii). In controls, rapamycin did not significantly change overall modularity (0.511 → 0.533) but moved cortical CD down and subcortical CD up, which is the opposite of its effect in MIR (Fig. 6d i, iv).

A posterior thalamic seed analysis (*P* < 0.01, cluster corrected) showed baseline increases in cortico-thalamic FC to primary motor, piriform, and primary somatosensory cortex (S1, S1DZ, hindlimb, forelimb, barrel, shoulder/neck), and increased FC from posterior thalamus to external globus pallidus, dorsal striatum, and contralateral anterior thalamus in MIR mice compared to control offspring (Fig. 6e). Rapamycin reversed several of these increases largely in thalamic-sensory cortical pathways. Voxelwise rsfMRI mapping of the thalamic seed FC data showed a pattern of increased FC strength and hyper-connectivity in MIR mice compared to controls. Global histogram analysis of this data using the difference between MIR and control mice (Fig. 6e) confirmed a large shift in FC strength for both cortical and subcortical structures in MIR mice, and a divergent effect of rapamycin treatment, both decreasing cortical hyper connectivity with the posterior thalamus and increasing connectivity for subcortical structures (Fig. 6f). Egr1 immunostaining 30 minutes after open field exploration validated these imaging data: Increased Egr1+ neurons occurred in motor cortex, striatum, and retrosplenial cortex in MIR mice, which is also consistent with the electrophysiological hyper-excitability, and decreased Egr1+ neurons occurred in medial amygdala (Supplementary Fig. 3d).

In summary, baseline FC in MIR was elevated in retrosplenial, auditory, and somatosensory cortex; cingulate cortex and amygdala; and insular cortex—congruent with the sensory, anxiety, and social phenotypes that rapamycin rescues^27^ (Fig. 4b–f, Supplementary Fig. 3b). Decreased baseline FC in MIR was seen between forelimb cortex, medial amygdala, striatum, globus pallidus, and periaqueductal gray—regions implicated in repetitive behavior—and was also altered by rapamycin (Fig. 6b, c). Few significant changes were seen in controls (Supplementary Fig. 3c), aligning with the lack of behavioral effects from rapamycin treatment (Figs. 3b and 4b–f, Supplementary Fig. 1a, b). Together, rapamycin appears to act acutely through rapid functional network normalization, predominantly affecting sensory processing networks.

### Analysis of gene expression alterations in MIR mice and after rapamycin treatment

Single-nucleus RNA sequencing (snRNA-seq) of adult somatosensory cortex showed differentially expressed genes (DEGs; Supplementary Fig. 2a–c), and DEG counts (Supplementary Fig. 2d, *p* < 0.05) were greatest between MIR mice and control offspring in oligodendrocytes, microglia, upper- and deep-layer excitatory neurons, and other excitatory populations, with more DEGs in excitatory than inhibitory neurons. mTOR pathway-related genes were significantly enriched in oligodendrocytes and their precursors, microglia, PV interneurons, upper-layer excitatory neurons, other (neurogranin) excitatory neurons, endothelial cells, and astrocytes (Supplementary Fig. 2e, *p* < 0.05). Pseudobulk analysis (LogFC>1, adj. *p* < 0.05, FDR < 0.1) identified ASD-, epilepsy/seizure-, and neuroinflammation-associated DEGs by cell type (Table 3), including downregulation of Kirrel and GRIP1—both also reduced in human ASD and other mouse models of SOR and repetitive behavior^50^.

**Table 3:** Single-nucleus sequencing analysis of significantly differentially expressed genes in MIR mice compared to control offspring is shown for autism-associated genes, epilepsy/seizure-associated genes, and neuro-inflammation-associated genes

Cell type	ASD-associated genes		Epilepsy/seizure-associated genes		Neuroinflammation-associated genes	
	Up MIR	Down MIR	Up MIR	Down MIR	Up MIR	Down MIR
Excitatory deep neurons	DBI HMGA2, CCND3, MAFB	NPAS4, MYOM2, EGR4, KIRREL*	EGR4, DBI, MAFB	NPAS4		
Excitatory upper neurons	FKBP5, CCND3, CD7	NPAS4, MYOM2, EGR4, COL11A1	FKBP5, EGR4	NPAS4, ACTN2		
Neurogranin Neurons	TBR1	ACTN2, MCM6, MRC2,DISP1, NOSTRIN, DISP1, KCTD18, KCNMB2,TTC28, NKAIN3, GPC6, CDK6, GRIP1*, EGFR, FILIP1, ST8SIA2	TBR1, DISP1	MCM6, DISP1, KCNMB2, NKAIN3, GRIP1*, ST8SIA2, EGFR,FILIP1		
Interneurons	WIPF1, SLC15A2, RPL4	EXD1, SLC17A8	WIPF1			SLC17A8
Microglia	FKBP5, E2F1, MCM6, C4B, PIK3C2G, FAM111A, DIAPH3,CCL12, HSPD1, TUBB5	SEMA4B, SLC44A2	FKBP5, FAM111A, DIAPH3	SEMA4B	XDH, CCL12, C4B, TUBB5	
Astrocytes	IRS2					
Oligodendrocyte s	HIF3A, FKBP5, C4B, CDH6, FGF1, DUSP1 BCAT1, KCNJ3	LYPD6, SLPI	DUSP1, KCNMA1		PHYHD1, C4B, MAT2a	SLPI
OPCs	HIF3A, FKBP5, NPAS1, HSP90AA1		FKBP5, ARF6			

All data use (LogFC >1, *P*_val_adj <0.05, FDR < 0.1) for abundance and significance criteria. *Sensory over-responsivity and repetitive behavior associated genes. *n* = 5 MIR, *n* = 6 control.

GSEA across all cell types showed enrichment of energy-dependent mTOR regulation and kainate receptor activity in MIR (Supplementary Fig. 5a). By cell type: all neurons showed enriched ion channel transport/homeostasis and decreased TGF-β and K⁺ channel signaling (Supplementary Fig. 5b); excitatory neurons showed enriched NF-κB, Notch, and TLR5/8 with decreased cytosolic Ca²⁺ levels and AP-2 transcription factors (Supplementary Fig. 5c); inhibitory neurons showed enriched GABA receptor activation and IL-1 signaling with decreased voltage-gated K⁺, NCAM-regulated neurite outgrowth, and gap junction regulation (Supplementary Fig. 5d). These changes suggest that cell-intrinsic mechanisms may drive neuron hyper-excitability, making neurotransmission and ion channel phenotypes in high mTOR-active cells (notably PV interneurons and upper-layer excitatory neurons) plausible targets for rapid rapamycin rescue (Supplementary Fig. 5e).

Bulk RNA sequencing of somatosensory cortex with and without acute rapamycin identified DEGs that can rapidly affect neuronal function, including GABA- and glutamate-related genes, transcription factors, and ion channel genes (LogFC>1, *p* < 0.05, FDR < 0.1; Table 4). Examination of ASD-risk genes^13,51^ showed significant up- or down-regulation in MIR mice of *TEK, PLAUR, ID1, Homer1, TEKT4, CNTNAP3, Slc35D3, Grin2c, CHD7, CDKN1A* (*p21*), and *NeuroD1* (Supplementary Table 1). Rapamycin rescued the over-expression of *TEK, PLAUR, CDKN1A*, and *ID1*, and the under-expression of *TEKT4, CHD7*, and *CNTNAP3* (Supplementary Table 2); it also decreased *Nestin, GEM, TIPARP*, and *APOLD1* and increased *HIF3A* and *PLIN4* (Supplementary Table 2). GSEA showed elevated glutamate receptor, oxytocin, PI3K, apoptosis, and angiogenesis pathways in MIR (Supplementary Fig. 5e), and biological-process GO showed elevated GPCR signaling and negative regulation of hydrolase activity but decreased cytokine regulation, amine metabolic processes, and autonomic nervous system development (Supplementary Fig. 5f). After rapamycin, GPCR signaling, innate immune, signal transduction, post-translational, and protein modification pathways were elevated (Supplementary Fig. 5g), and pathway GSEA showed increased neurotransmitter regulation, lipid regulation, and growth factor response with decreases in epithelial and skeletal systems (Supplementary Fig. 5h).

**Table 4:** Bulk sequencing analysis of significantly differentially expressed genes in MIR mice compared to control offspring is shown for transcription factors, GABAergic genes, Glutamatergic genes, ion channel genes, potassium ion genes, sodium ion genes, and calcium ion genes, which may affect neuronal excitability

Differentially Expressed Genes MIR vs CNTL													
Transcription Factors		GABAergic genes		Glutamatergic Genes		Ion Channels		Potassium Channels		Sodium Channels		Calcium Channels	
Up	Down	Up	Down	Up	Down	Up	Down MIR	Up	Down	Up	Down	Up	Down MIR
Runx2 Nkx1-2 E2f8 Trpc6 Tcf19	Nkx2-1 Nkx2-2 Myt1 Helt Eya2 Asic4 Tfap20 Tfap2a		Gabre Gabrq		Grik1 Grik2 Grin2c Grid2 Grm1 Grm4	Slc22a3 Slc22a4 Asic4 Clcnka Slco2b1 Trpc6	Trpc6 Clic6 Trpc7 Trpv1 Trpm3 Slc35d3	Kcnh3 Kcnv1	Kcne1l Kcnj5 Kcnab1 Kcnk10 Kcnj13		Slc4a5 Slc10a4 Slc8a3 Scn5a Scnn1g	Cacnb3	Cacng5 Cacna2d2

All data use (LogFC > 1, *P* < 0.05, FDR < 0.1) for abundance and significance criteria. *N* = 8/group/treatment.

These findings identify DEGs controlling excitatory/inhibitory neurotransmission, transcription factors, and ion channels as candidate substrates for the hyper-excitability and aberrant functional connectivity in MIR mice are rapidly altered by acute rapamycin and represent promising therapeutic targets.

## Discussion

Exposure to early gestation mild maternal inflammation in CD1 mice recapitulates multiple ASD phenotypes: early juvenile brain overgrowth followed by regional cortical thinning with enlargement of hippocampal, basal ganglia, and ventricular structures^42,43,52^; increased seizure susceptibility^53^, chronically elevated blood cytokines and brain microglia^6,18–20,22,51,54^; and social, communication, anxiety, and repetitive behaviors^27^. We have now extended these findings to show that MIR mice demonstrate significant SOR and sensory processing dysfunction. SOR is an extremely impairing clinical condition marked by avoidance and/or sensitivity to sensations and is present at high rates across the autism spectrum. SOR is associated with other core features of ASD, such as difficulties with social cognition and behavioral and emotional regulation^55^. Our findings add an acquired model of ASD to the numerous genetic models that exhibit SOR, and demonstrate that inhibition of mTOR signaling temporarily ameliorates this condition.

It may be surprising that a single exposure to low-level maternal inflammation, at a dose insufficient to cause sickness or behavioral changes in the dam or to reduce litter size, can produce such lasting effects on offspring's brain and behavior. However, we and others have found that early* in utero* exposure sets up chronic dysregulation in systemic immune function^56^, suggesting a chronic inflammatory state across the life span in MIR mice. Both clinical and pre-clinical studies have reported some success in the treatment of ASD behavioral impairments with an anti-inflammatory drug, pioglitazone, which improved abnormal phenotypes along with reducing neuro-inflammation^57^. Induction of systemic and brain inflammation in adult wildtype/control mice has not been found to produce any of the ASD-associated abnormal behaviors like those that we see in MIR mice where inflammation was induced *in utero* and persists chronically^58^. Therefore, an elevated inflammatory state in the adult brain does not, on its own, explain most of the MIR phenotypes, suggesting that there are likely important vulnerable periods in brain development where inflammatory exposure is especially deleterious and/or the effects of chronic inflammation and increased mTOR pathway activity have the strongest effects. It is well known that chronic neuro-inflammation can contribute to significant brain pathophysiology, and that maternal inflammation is also known to be a significant risk factor for both ASD and brain overgrowth^6,22–26,59^. Although we initially hypothesized that chronically elevated microglia may drive the MIR phenotype^60^, microglia ablation rescued behaviors in young but not older adults, while rapamycin rescued both. Microglia therefore likely play a role in establishing physical and functional changes but are not the primary cause of ongoing dysfunction. Thus, the effects of MIR exposure on behavior are complex and evolving over the lifespan, as indicated by early brain overgrowth followed by later regional decreases in brain volume. Chronic post-natal neuro-inflammation and mTOR activation in the brain have also been associated with epilepsy and a disturbed excitatory/inhibitory balance^53^, which is consistent with the cortical hyper-excitability and increased seizure susceptibility that we observe in MIR mice.

Given the long-term elevation in mTOR signaling in MIR offspring and the similarity of some ASD-associated brain and behavioral phenotypes seen in *TSC* and *PTEN* mutant mice that also have increased mTOR pathway activation, we examined the role of mTOR signaling in MIR pathology. The mTOR inhibitor, rapamycin, is known to rescue several abnormal ASD-associated behaviors in murine genetic models of *PTEN* and *TSC* mutations even in adult mice with existing/ongoing brain physiological abnormalities like brain overgrowth^6,29,31^. Here we observed an unanticipated rapid effect on MIR behaviors. After confirmation that the effects of rapamycin were based on CNS-mediated inhibition of the mTOR pathway using the peripheral-acting RapaBlock inhibitor, we focused our mechanistic search on rapidly modifiable physiological and functional processes rather than the structural remodeling that longer rapamycin treatment regimens produce^7,29,30,61^, potentially revealing mechanisms obscured by chronic dosing.

While synaptic strengthening/weakening through altered levels of neurotransmitter release or post-synaptic receptor expression can occur on short (minutes to hours) time scales, widespread physical synaptic remodeling is an unlikely mechanism for acute rapamycin effects. This led us to examine changes in neuronal gene expression, excitation, and functional activation and organization at the circuit level as possible fast-acting mechanisms that could underlie the brain and behavioral dysfunction rescued by acute rapamycin treatment in the MIR model. Single-nucleus sequencing showed mTOR pathway gene expression was upregulated across microglia, astrocytes, oligodendrocytes and their progenitors, upper-layer excitatory neurons, and parvalbumin interneurons, implicating cell-type-specific pathway dysregulation. Many genes associated with autism, epilepsy, and neuroinflammation were differentially expressed in upper and deep-layer excitatory neurons. Bulk sequencing identified rapidly modifiable changes in transcription factors, ion channels, and neurotransmitter synthesis genes, and several ASD risk genes^13,51^ were altered and reversed by acute rapamycin, which have known associations with brain size (*ASPM, KIF14*), epilepsy (*PLAUR, MAST3, HOMER1, ACE, ID1, CHD7, CDKN1A*), intellectual disability (*CHD7, TEKT4*), sensory processing (*PLAUR*), synaptic plasticity (*HOMER1, CNTNAP3, IQGAP3*), and neuroinflammation (*TEK, CNTNAP3, ACE, CDKN1A*). The rapid rescue of neuron hyperexcitability and seizure susceptibility within the same time frame as behavioral rescue suggests that restored excitation and inhibition balance underlies normalization of abnormal sensory and other ASD-related behaviors.

The majority of the circuit-level rs-fMRI differences in MIR mice were FC increases involving primarily sensory rather than motor circuits, despite the prominent repetitive motor behavioral phenotype. While M1 motor cortex showed increased basal ganglia FC, the dominant cortico-striatal and cortico-limbic increases originated from sensory cortex, and the involved basal ganglia structures themselves serve sensory as well as motor roles^62^. The most prominent changes were between thalamus and primary sensory cortices and salience network structures, aligning with the SOR phenotype. Therefore, given the close clinical correlation between repetitive behaviors and sensory sensitivity and the beneficial, self-regulation role repetitive behaviors can play^63^, our findings that sensory dysregulation is the more dominant brain phenotype in MIR mice further support the idea that repetitive behaviors may develop in response to SOR rather than being a primary dysfunction in ASD. This sensory-dominant hyper-connectivity profile aligns with a recent cross-species fMRI analysis^64^ that identifies a reproducible autism related hyper-connectivity subtype linked to immune, transcriptional, and mTOR-related pathway activation, distinct from a hypoconnectivity subtype tied to synaptic dysfunction. mTOR signaling was among the few synaptic-related pathways enriched in the hyper-connectivity subtype, positioning it as a molecular bridge between immune activation and functional network alterations. The thalamic, sensory, and salience network involvement we observed in MIR mice mirrors the hyper-connectivity signatures seen across multiple immune- and mTOR-linked mouse models of autism and in human data^64^. Furthermore, acute rapamycin reduced cortical FC toward control levels but further increased subcortical FC, indicating that behavioral rescue does not depend on full FC normalization. This is likely related to the fact that brain structural abnormalities remain, and acute rapamycin treatment is acting in the context of these persistent physical changes. Longer treatments with rapamycin in TSC mutant mice^7^ show that rescuing physical synaptic abnormalities produces a greater return to control FC patterns. These circuit-level insights into dysregulation in MIR mice and the pattern of network changes associated with behavioral recovery that we have identified are potentially useful neuromodulatory targets for non-pharmacological-based treatments like transcranial magnetic stimulation and low intensity focused ultrasound for ASD and SOR.

In a graph theory network analysis of community architecture, we found that MIR offspring have a larger number of functional modules compared to control offspring, suggesting that their brain circuitry is more segregated into distinct functional sub-networks in subcortical brain regions. However, the nodes within these subcortical modules have higher classification diversity values than in controls, indicating highly flexible membership between modules within the MIR group, reflecting increased information transfer across the brain, and greater functional integration. The opposite is true within the cortex of MIR mice, with a lower classification index indicating reduced information transfer across the brain. The combination of increased module number and altered intra-module nodal diversity suggests a complex reorganization of brain networks, which may reflect both deficits and compensatory adaptive changes in functional connectivity. Clinical fMRI studies in ASD show similar patterns of increased module number, intermodular connections, and module diversity^65,66^, demonstrating another cross-species phenotype that is present in our inflammatory, acquired model of ASD.

It has been suggested that the functional modular reorganization in the ASD brain may be a compensatory mechanism for restoring functional homeostasis at the circuit level in response to physical connectivity changes, and that therapeutic normalization of this altered modular organization would therefore be likely to worsen ASD dysfunction^65^. However, our acute rapamycin treatment data suggest otherwise. The rapid rescue of this abnormal network modularity in MIR mice following rapamycin treatment was one of the largest effects of the pharmacological treatment on the MIR model of brain dysfunction. Both the number of modules and the distribution of the nodal connectivity within these modules were returned to control levels after acute rapamycin, and this treatment restores information flow and network stability within the same time frame that we observe behavioral and electrophysiological rescue from acute rapamycin. Therefore, our findings suggest that the modular organization of functional brain networks are related to behavioral traits in ASD, and that this may be a central functional mechanism underlying inflammation-mediated behavioral dysfunction in several core autism phenotypes.

In summary, our data support a plausible etiology of mTOR-mediated dysfunction in ASD revealed through acute rapamycin: (1) in utero MIR exposure produces chronic postnatal neuroinflammation, (2) sustaining elevated mTOR signaling, (3) which increases neurogenesis and synaptogenesis during a critical developmental window, (4) producing brain overgrowth and abnormal physical connectivity, and (5) compensatory functional brain reorganization. Maternal inflammation could therefore account for the non-macrocephalic brain overgrowth seen in early childhood ASD^21,41^, with even mild growth alterations producing aberrant connectivity and brain-wide functional network and behavioral changes. Although this dysfunction is identifiable at the network level, it depends on processes operating at the immune, cellular, and subcellular (gene expression, signaling) levels. While longer mTOR suppression also reverses physical maladaptive plasticity^7^, acute rapamycin treatment identifies additional therapeutic targets across these levels and shows that even the adult MIR brain is amenable to rapid functional and excitability normalization, rescuing core and comorbid ASD behaviors without requiring lasting structural change. Therefore, restoring excitation and inhibition balance and normalizing sensory network modularity may be key mTOR-related targets for therapeutically addressing both SOR and compensatory repetitive behaviors in ASD.

## Methods

The following methods have been communicated using the ARRIVE 2.0 guidelines^67^.

### Study design

The primary objective of this study was to evaluate the low-dose, early gestation model of maternal inflammation on offspring studied chronically from young adult (P60-90) to old adult (P200-400), to assess the effects of acute rapamycin treatment at these ages, and to identify mechanisms contributing to its rapid effects on behavior. Our main hypotheses were that the MIR model replicates multiple phenotypes relevant to autism (brain overgrowth, chronic systemic inflammation, microglia contributions to dysfunction, sensory dysregulation, autism-associated gene expression changes, etc.), and that the rapid effects of rapamycin treatment could identify novel mechanisms of dysfunction that are different from published chronic effects of rapamycin treatment involving physical synaptic remodeling.

### Experimental framework

A parallel group design was used for most experiments, where there are separate MIR and Control offspring groups for every treatment and for different ages (young adult and old adult). The fMRI experiments used a within-subjects crossover design where mice were first tested at baseline and then again after rapamycin treatment. The experimental unit used for all studies is individual mouse offspring. Multiple litters of maternal inflammation-exposed offspring and vehicle control-exposed offspring were generated, and equal numbers of male and female offspring were randomly selected from all litters to generate each experimental group. Some mice were used as young adults, and other animals from the same litters were kept for experiments on older adult mice. These experiments were performed with 52 total litters generated over several years of experimentation (see Supplementary Table 3). Every new litter generated was evaluated for repetitive behaviors in an open field to confirm that the MIR phenotype was reproduced in the new offspring before using them for experiments, including non-behavior experiments. No MIR litters failed to display a significant repetitive behavior phenotype in offspring, demonstrating the reproducibility of the model. Because batch variations in LPS strength/effectiveness have been reported we used the same batch of LPS, stored in small aliquots at −80 °C, and made up fresh in saline for injection for every MIR litter generated. See Fig. 1a for an outline of this experimental timeframe.

### Sample size

Sample sizes were determined based on our own previous experiments and relevant experiments published in the literature to achieve a statistical power of 0.8 with an alpha of 0.05 to detect an effect size (Cohen’s d) of 1.2. The significance and the effect size were reported for every result in the figure legends and results section. Group numbers have been reported in all figure legends, and individual data points are plotted in the result figures.

### Inclusion and exclusion criteria

Mice from multiple litters were randomly assigned to each group, and no selection criteria were used. An equal number of male and female mice were used in each group. Mice injured due to fighting in home cages, which happened occasionally, were excluded from the study due to possible confounding effects of single housing and medical treatments. No animal exclusions were necessary due to a failure to perform the behavioral tests. Because we used an outbred strain of mice (CD1) that has some genetic variability, a range of phenotype severities was expected. Therefore, outlier data and outlier animals were not removed from any experiment since these mice likely represent the possible range of outcomes in the model.

### Blinding

Animal group identification was not written on cage cards so that researchers were blinded to experimental groups during data collection. Group identification was known to the investigator analyzing the data only after all data had been collected. The treatment group, but not the litter group, was known when rapamycin or vehicle treatment was administered. However, due to the development of extreme repetitive behaviors in a third of the MIR offspring at older ages, it was not possible to be fully blinded to the group for the old-adult mice. MIR mice at older ages also frequently weighed less due to constant repetitive activity compared to control mice, which normally become more sedentary and increase in weight significantly with age. MIR mice at young adult ages were not readily identifiable by inspection of home cage behavior.

### Experimental animals

Time-mated, pregnant CD-1 wild-type mice were obtained from Charles River Laboratories (CA, USA). On day E9 of gestation, pregnant dams received a single intraperitoneal (I.P.) injection of low-dose lipopolysaccharide (LPS, E. coli serotype 0111:B4; Sigma) at 0.008 mg/kg dissolved in sterile saline, or an equivalent volume of sterile saline (Vehicle Control). This low dose was chosen as it induces a cytokine response without causing overt sickness behavior in the pregnant dam and mild brain overgrowth in the offspring (15–20% greater than vehicle control offspring but not macrocephalic), as we have previous published^27^. The same batch of LPS was used for the generation of all MIR litters in this manuscript. A different batch of LPS used at the same dosage generated MIR offspring in our previous publication of this model, demonstrating its reproducibility. Litter sizes ranged from 9–16 offspring, and mice were weaned at post-natal day 21. After weaning, mice from the same litter were socially housed together 2–4 per cage by sex. Mice were randomly selected from multiple litters to form experimental groups, and equal numbers of male and female mice were used in each group for all experiments. Separate cohorts of mice were used at young adult ages (P60-90) and old adult ages (P200-400) for specific experiments. Mouse age group have been reported in figures and figure legends. The CD-1 strain of mice was used because of the similarity that their stem and progenitor brain cells have to human brain Hif1-alpha redox responses that are relevant to an inflammation model, their large litter sizes, and their consistent responses in behavioral testing, as seen in our original publication of this MIR model^27^.

#### Husbandry details

Animals were socially housed under standard pathogen-free conditions on a 12-hour light/dark cycle at a constant temperature (20–26 °C) and humidity (50%). They had ad libitum access to standard rodent chow and sterile water. Cages contained aspen chip bedding and were provided with nesting material for enrichment. At the end of all experiments, mice were euthanized by CO_2_ and rapid decapitation or by perfusion-fixation following pentobarbitol overdose and succession of respiration. No unexpected animal deaths occurred, and the only adverse events that occurred were due to a small number of cases of animal fighting in the home cages outside of experimental work.

### Pharmacological treatments

All pharmacological treatments were administered to the mice as young adults (P60-90) or old adults (P200-400), apart from the juvenile mice (P25) in the reciprocal social interaction test (see Supplementary methods and Supplementary Fig. 3b). Juvenile mice are commonly used for this test because they are less territorial and more likely to engage in reciprocal behaviors without aggression or dominance behaviors.

### Rapamycin

Rapamycin (5 mg/kg; Sigma-Aldrich) or vehicle (DMSO) was administered via intraperitoneal (I.P.) injection in the flank. For all acute treatment experiments in young and old MIR and control offspring, this was given 2 hours prior to data collection. The rapamycin dose was chosen based on previous published work that showed behavioral rescue in genetic murine models of autism in adult offspring^6^. For the chronic treatment experiment, daily I.P. injections (5 mg/kg) were given for 5 weeks.

### Microglia depletion

Young and old adult mice were fed a diet containing the CSF1R-inhibitor Plexxikon 5622 (Plexxikon-Daiichi, San Francisco) formulated at 1200 mg/kg in rodent chow (AIN-76A; Research Diets, New Brunswick, NJ) or control AIN-76A chow ad libitum for 3 weeks prior to and during behavioral testing. Microglia depletion was confirmed (>98%) with post-mortem immunohistochemistry with the myeloid cell marker, IBA1 (Wako 019-19741).

### PTZ treatment

Mice were tested for seizure susceptibility using an escalating pentylenetetrazole (PTZ, Millipore P6500) dosing protocol (0–40 mg/kg, I.P.) modified from Shimada and Yamata^68^. Following intraperitoneal injection with PZT, a GABAA receptor antagonist, animals were monitored continuously for 30 minutes. High doses of PTZ are known to induce an acute, severe seizure, but sequential injections of a sub-convulsive dose are used for the development of chemical kindling, an epilepsy model. With this method, vulnerability to PTZ-mediated seizures can be estimated. PTZ was dissolved at 2 mg/mL in sterile 0.9% (w/v) NaCl and prepared on the day of use. Mice were injected i.p. with a starting dose of 10 mg/kg and then injected every 2 days again, increasing the dose by 10 mg/kg. Mice were monitored for the following seizure-related escalating behaviors for 30 minutes following the injection: (0) no seizure behaviors, (1) immobilization, (2) head nodding and partial myoclonus (sudden, brief involuntary twitching or jerking of a muscle or group of muscles), (3) continuous whole body myoclonus, (4) rearing or tonic seizure (falling on the side followed by forelimb tonic contraction and hindlimb tonic extension), (5) tonic-clonic seizure with rushing and jumping. Both control and MIR mice were given escalating doses of PTZ until all mice from one group were displaying seizure-like activity anywhere on the 1–5 scale, and the number and severity of seizure symptoms at this maximum PTZ dose (40 mg/kg) were then compared between groups to determine relative induced seizure susceptibility. The effects on seizure-behavior level at the maximum PTZ dose after acute rapamycin treatment in MIR and vehicle-control offspring were also compared. Humane endpoint: the experiment was to be terminated for an animal if it experienced a tonic-clonic seizure lasting longer than 60 seconds or failed to recover normal posture within 5 minutes but no animals met these criteria. The primary outcome of the PTZ experiment was identifying the number of mice which reached each seizure-behavior level (1-6) with and without rapamycin treatment at the maximum PTZ dose (40 mg/kg). A quantitative seizure score was generated for each mouse based on what seizure level (1–6) the mouse was at for each dosage of PTZ and PTZ + rapamycin for statistical analysis in supplemental results (Supplementary Table 4).

### Blood cytokine analysis

Blood was collected from pregnant dams on E9 at 12-hours after LPS injection (0.008 mg/kg, I.P.) and from MIR and control offspring as adults. Blood plasma was separated and frozen before analysis by the UCLA Pathology Immune Assessment Core using the Luminex’s xMAP® Mouse 32-plex Cytokine/Chemokine Panel Immunoassay according to standard assay methods. The primary outcome was the blood cytokine levels resulting from acute LPS injection in pregnant dams and in adult MIR-exposed and vehicle-control-exposed offspring. See supplemental methods for full cytokine names with abbreviations

### Regional brain microdissection and western blot

The sensory-motor cortex, amygdala, and striatum were microdissected from adult MIR and control offspring and from a separate cohort of adult offspring after acute rapamycin treatment and flash frozen for standard western blot analysis. Tissue homogenates (cytosol) were lysed in RIPA buffer (25 mM Tris-HCl, 150 mM NaCl, 1% NP-40, 1% sodium deoxycholate, 0.1% SDS, pH 7.6 at 4 °C) containing a cocktail of protease inhibitors (Calbiochem). Equal amounts of protein were separated by SDS-PAGE (4–12% Bis-Tris gels, Invitrogen) and transferred to PVDF membranes (Invitrogen), blocked in TBST plus 5% non-fat milk and then incubated with the following primary antibodies: phospho-S6 (S235/236; 1:2000, Cell Signaling, #4858), S6 (1:2000, cell signaling, #2217), and b-actin (1:2000, Cell Signaling, #4970) overnight at 4 °C. The membranes were then washed with TBST and incubated for 1 h at room temperature. The washed membranes were then treated with an enhanced chemiluminescence detection reagent (Thermo Scientific). All Blots were developed using ChemiDoc XRS+ Molecular Imager (BioRad) and analyzed using Quantity One software (BioRad). Band densities were normalized to the total amount of protein loaded per lane using Sypro Ruby (BioRad). The three regions of brain tissue were pooled from MIR and vehicle-control offspring for comparison of phospho-S6 protein levels normalized to total S6 protein and GAPDH protein levels in mice treated with a 2-hour rapamycin or vehicle treatment.

### Behavioral testing

Mice were housed four per cage on a 12-hour light/dark cycle with ad libitum access to food and water. All behavioral experiments were performed during the light phase (10:00–16:00) by a blinded experimenter. Animals were habituated to handling for 3 days prior to experiments and habituated to the testing room for 60 minutes the day before and the day of any test. Mice which took part in the open field, light/dark, and Von Frey testing were habituated to the testing apparatus in a 10-minute habituation session on two consecutive days before testing. A battery of tests was performed on the same cohort of mice in order of least to most stressful, with at least 48 hours between tests: open field, tactile light/dark box, Von Frey, and finally PPI. A separate cohort of young mice (P25) was used for the juvenile reciprocal social interaction testing. Behavioral equipment was cleaned between animals with 70% ethanol followed by tap water, and then dried to remove residual odor cues.

### Open field repetitive behaviors

Measurement of the cumulative time spent performing repetitive behaviors in an open field chamber was performed during a 10-minute session according to the methods of Peñagarikano et al^69^. The total amount of time that the mice spent performing repetitive behaviors (grooming while stationary or while moving on all body regions, and repetitive circling seen in some of the older mice) was measured. The primary outcome measure was the percentage of total testing time spent performing repetitive behaviors of any kind. A square open-field arena (40 × 40 × 40 cm) constructed of opaque gray PVC was used. The floor was non-textured and non-absorbent, and no bedding was present. Illumination at the arena was set to 50 lux during testing.

### Von Frey SODU

The Von Frey simplified up-down (SODU) method from Bonin^70^ was used for assessing mechanical (tactile) sensitivity on the hind paws. We used standard Von Frey filaments (Harvard Apparatus), which are thin nylon fibers of varying diameters which are used to apply a controlled mechanical force onto the mouse’s paw to assess the force threshold at which the animal senses (notices) the touch and lifts/withdraws its paw. The paw withdrawal threshold (PWT) is a quantified measure of fiber force strength at which this behavioral response is observed. A wire mesh platform was used as the testing area. Mice were habituated to this platform while in a clear plastic holding chamber for 1 hour prior to testing. During the test, the mice were placed on top of the wire mesh platform and allowed to freely explore. A low threshold Von Frey fiber was used to touch the plantar surface of one of the hind paws of the mouse through the mesh frame for 1–2 seconds. The response of the mouse was recorded (either lifting its paw or no response). If the mouse withdrew its paw, then the next lower filament was used for the second touch. If the mouse did not respond, then the next higher filament was used for the second touch. This process was repeated for five touches in total. Outcome variables: we used the final response to the fifth filament to estimate the PWT. If the mouse did not withdraw its paw on the fifth touch, 0.5 was added to the logarithmic value of the fifth filament’s diameter. If the mouse withdrew its paw on the fifth touch, 0.5 was subtracted from the logarithmic value of the fifth filament’s diameter. The resulting logarithmic value was converted to a linear scale to obtain the PWT in grams. The primary outcome measure was the mechanical threshold in grams at which the mice felt/responded to the fiber pressure on the bottom of their hind paw on the 5th touch.

### Light/dark box tactile avoidance testing

The tactile avoidance light/dark box is a novel method developed to examine the tactile sensitivity of the paws and avoidance behaviors. This test used the classic light/dark chamber box, which is traditionally a test for anxiety in mice where normal mice will explore both light and dark chambers, but mice prefer the dark chamber, and mice with heightened anxiety will spend much more time in the “safe” dark chamber compared to normal controls^71^. We adapted this test to use either a smooth plastic floor or a rough textured floor in one chamber, using rough, 80-grit anti-slip stair adhesive strips. The time mice spend in the left or right chamber when both are lit and the floor is smooth plastic (trial 1), when both are lit but the floor in one chamber has the rough texture (trial 2), when one chamber is dark but both floors are smooth (trial 3–the classic light/dark box test), and finally when the dark (preferred) chamber had the rough textured floor and the lit chamber had a smooth floor (trial 4) was measured. Each trial was 10-minutes long and was performed 24 hours apart, with the order of the trials randomized for each group. The primary outcome measures were the time spent in each chamber for each trial. Spending less time in the normally preferred dark chamber when the floor was a rough texture but not when the floor was smooth, demonstrated an aversive tactile sensitivity and avoidance behavior. Each chamber was a square arena (40 × 40 × 40 cm) constructed of black acrylic. Illumination on the arena floor was set to 150 lux during all trials.

### PPPI and habituation to sensory startle

The San Diego Instruments SR-Lab Startle Response System was used to test for sensory gating ability in the different animal groups. The system software is programed to produce a randomized combination of a low-intensity airpuff onto the fur on the back of the mouse as the pre-pulse sensory “warning” followed by a loud (20 dB, startle-inducing) sound at an increasing distance in time (50–1000 ms) between the pre-pulse airpuff and the audible startle pulse according to the methods of Orefice, et al.^45^. Startle-only pulses were also included at the beginning, middle, and end of the full test. The amplitude of the startle response by the mouse was measured by a sensitive gyroscope within the soundproof testing chamber. Mice were placed within the startle enclosure, and testing time was ~30 minutes per session. When sensory gating is normal, the closer in time that the tactile pre-pulse warning (light airpuff) is given to the auditory startle sound, the greater the inhibition (decrease) there is in startle response from the mouse. Primary outcome measures: The SR-LAB software automatically records the mouse’s startle response (i.e., movement detected on the platform). This data was then used to calculate the PPI percentage by comparing the startle response amplitude in the presence of the changing time distance of the prepulse stimulus. In addition, a startle only (without prepulse warning) at the beginning, and end of the PPI program was used to test for habituation to startle over time and was shown as a ratio (amplitude of end startle: amplitude of beginning startle) where values > 1 indicate a lack of habituation.

### Single-cell and bulk genetic sequencing and analysis

Mice were euthanized according to approved protocols, and sensory-motor cortex tissue was microdissected from MIR and control offspring and flash frozen before being prepared for sequencing analysis. For bulk RNA sequencing, 12 MIR and 12 control mice were given rapamycin or vehicle 2 hours before euthanizing and flash freezing the brain tissue for sequencing. RNA was isolated using a Qiagen RNeasy Mini kit (Cat. 74106) followed by library preparation using a KAPA Stranded mRNA-Seq Kit (Cat. KR0960). The sequencing libraries were pooled and sequenced to generate a minimum of 220 M 50 paired-end reads on NovaSeq6000 (Illumina). Reads were aligned to the mouse mm10 genome reference using STAR aligner (v2.4.0). Read counts for refSeq genes were generated by HT-seq 0.6.1. Various QC were performed after alignment to examine the level of mismatch rate, mapping rate to the whole genome, repeats, chromosomes, key transcriptomic regions (exons, introns, UTRs, genes), insert sizes, AT/GC dropout, transcript coverage and GC bias. Outliers were removed based on QC results. Differential expression analysis was conducted with R-project and the Bioconductor package EdgeR. DEGs between groups were defined as those meeting all of the following criteria: false discovery rate (FDR) < 0.05, LogFC > 0.5, and *p* < 0.05 threshold. We also tested for overlap between these DEGs and high-confidence autism-associated genes in ASD databases^13,51^.

For single-nucleus RNA sequencing nuclei were isolated from frozen brain tissue using iodixanol-based density gradient centrifugation and submitted to UCLA Technology Center for Genomics and Bioinformatics for library preparation (via Chromium Single Cell 3’ v3 kit from 10x Genomics) and sequencing (via NovaSeq 6000 S2 platform from Illumina). Cell Ranger (10x Genomics) was used for alignment, filtering, barcode identification, and generation of gene-cell matrices for each sample. Ambient RNA was removed using CellBender. Pre-processing (minimum cells 3, minimum features 200, doublet removal via scDblFinder^72^, maximum mitochondrial content 5%), integration (3000 features), and clustering (resolution 0.8) were performed using the R package Seurat. Canonical lineage markers were used to identify cell types (microglia, astrocytes, oligodendrocytes, pericytes, endothelial cells, upper-layer and deep-layer excitatory neurons, and interneurons). Differential expression testing was performed using the pseudobulk approach (edgeR with likelihood ratio test) via the R package Libra. Gene set enrichment analysis (GSEA) was performed to assess differential enrichment of the “Hallmark mTORC1 Signaling” gene set^73,74^ using the R package fgsea^75^. The above comparisons underwent correction for multiple comparisons using the native functionality of the respective packages. Significance was defined as FDR or adjusted *p* < 0.05.

### Brain slice electrophysiology

Adult (2–6 mo) control and MIR mice (*N* = 6 animals per group) were deeply anesthetized with isoflurane and perfused transcardially with ice-cold, high-sucrose slicing solution containing (in mM): 26 NaHCO_3_, 1.25 NaH_2_PO_4_, 208 sucrose, 10 glucose, 2.5 KCl, 1.3 MgCl_2_, and 8 MgSO_4_. Mice were swiftly decapitated, brains extracted, and immediately placed in oxygenated high-sucrose slicing solution. Coronal slices containing both sensorimotor cortex and dorsolateral striatum were cut at 300 μm and transferred to an incubating chamber containing artificial cerebrospinal fluid (ACSF) (in mM): 130 NaCl, 5 KCl, 1.25 NaH_2_PO_4_, 26 NaHCO_3_, 2 MgCl_2_, 2 CaCl_2_, and 10 glucose) oxygenated with 95% O_2_–5% CO_2_ (pH 7.2–7.4, osmolality 290–310 mOsm/L, 32–34 °C). Slices were allowed to recover for 60 min and electrophysiological recordings were obtained at room temperature. Cortical pyramidal neurons (CPNs) in layers 2/3 or striatal medium-sized spiny neurons (MSNs) were visually identified using infrared illumination with differential interference contrast optics (IR-DIC). Whole-cell patch clamp recordings in voltage- or current-clamp modes were obtained using a MultiClamp 700B Amplifier (Molecular Devices) and the pCLAMP 10.5 acquisition software (Molecular Devices). For CPN recordings (in voltage and current clamp), the internal pipette solution contained (in mM): 112.5 K-gluconate, 4 NaCl, 17.5 KCl, 0.5 CaCl_2_, 1 MgCl_2_, 5 ATP (dipotassium salt), 1 NaGTP, and 10 HEPES (pH 7.2, 270–280 mOsm/L). For striatal MSN recordings (in voltage clamp only), the internal pipette solution contained (in mM): 130 Cs-methanesulfonate, 10 CsCl, 4 NaCl, 1 MgCl_2_, 5 MgATP, 5 EGTA, 10 HEPES, 5 GTP, 10 phosphocreatine, and 0.1 leupeptin (pH 7.2, 270 mOsm). Electrode impedance was typically 4–5 MΩ in the bath. Cells with access resistance exceeding 25 MΩ were discarded. Cells were first voltage clamped at −70 mV to assess passive membrane properties (membrane capacitance, input resistance and decay time constant) and measured by applying a 10 mV depolarizing step voltage command and using the membrane test function integrated in the pClamp10 software. Active membrane properties of CPNs (resting membrane potential, intrinsic excitability, and rheobase) were determined in current clamp mode and by injecting current pulses of increasing intensity. Rheobase (minimal current intensity required to evoke an action potential), consisted of a single depolarizing current pulse (5 ms duration) of increasing intensity (Δ = 15 pA). Spontaneous synaptic currents were recorded at −70 mV holding potential in the presence (MSNs) or absence (CPNs) of Bicuculline (BIC, 10–20 µM), a competitive antagonist of GABAA receptors, to better isolate glutamatergic events. Spontaneous synaptic currents were analyzed using the MiniAnalysis software (version 6.0, Synaptosoft, Fort Lee, NJ). The effects of rapamycin ex vivo (1–2 µM) on synaptic activity were examined by incubating slices from control or MIR mice for 2 hr before and during electrophysiological recordings. In vivo rapamycin treatment was also given to MIR mice (5 mg/kg, I.P.) or vehicle (DMSO) 2 hours before decapitation and slice electrophysiology. Primary outcomes for synaptic activity were the frequency and amplitude of spontaneous excitatory postsynaptic currents. For intrinsic excitability, the primary outcomes were rheobase measurements and the firing patterns following injection of depolarizing current pulses.

### Magnetic resonance imaging

Adult MIR and Control offspring were imaged using a 7 Tesla Biospec small animal MRI system using Paravision 5.2 software (Bruker) at the UCLA Brain Mapping Center. Mice were briefly anesthetized with 2% isoflurane vaporized in oxygen flowing at 1 L/min, then placed on an MRI-compatible cradle and a single-channel surface coil (Bruker) secured over the head. For resting-state scans, a 3 mm-thick agar gel cap (Sigma, 3% in distilled water) was placed between the head and the surface coil, in order to reduce signal distortion in the blood-oxygen-level-dependent (BOLD) signal^76^. To minimize time-dependent effects of the anesthetic on the BOLD signal, these initial steps were performed within a 10–15-minute time window. Isoflurane was gradually discontinued, and sedation was initiated with a single subcutaneous (s.c.) injection of dexmedetomidine (Dexdomitor®, Zoetis; 0.15 mg/kg) followed by continuous s.c. infusion at 0.3 mg/kg/hr throughout the duration of the imaging as described in a previous publication^32^. Respiration and body temperature of the mouse were continuously monitored remotely, and maintained in a physiological range (37 ± 1 °C; Small Animal Instruments Inc.) by a homeothermically-controlled forced warm air over the body (SA11 Instr, Inc., USA). At the end of the imaging session, sedation was reversed by atipamezole (Antisedan®, Pfizer) at 1.5 mg/kg (i.p.). Data were acquired using the S116 Bruker gradients (400 mT/m) in combination with a single-channel surface coil (described above) and a 72 mm birdcage transmit coil. An initial series of scans was performed to confirm proper head position, then localized FASTMAP shimming was performed to improve field homogeneity. T2-weighted structural scans were acquired with a Rapid-Relaxation-with-Enhancement (RARE) sequence (RARE factor=8, Echo time (TE) = 56 ms, repetition time (TR) = 6020 ms, four averages, data matrix = 128 × 128 in a field-of-view (FOV) = 20 × 20 mm, slice thickness = 0.5 mm, 14 slices, FA = 90 deg, bandwidth (BW) = 50 kHz). Then functional (BOLD) data were acquired using the same image geometry as the structural scans, with a one-shot, interleaved, gradient-echo echo planar imaging sequence with the following parameters: TE = 19 ms, TR = 2,000 ms, FA = 30 degrees, BW 400 kHz and a data matrix of 90 × 60) in a FOV of 20 m × 20 mm. 10 dummy scans were used to allow the T1 signal to reach steady-state prior to signal acquisition, after which 450 repetitions were acquired for 15 mins. For each mouse, baseline data was collected and on the following week rapamycin (5 mg/kg) or vehicle control (DMSO) was injected i.p. 2 hours before imaging.

### MRI analyses

Data were converted to nifti format and entered into a Python-scripted, preprocessing pipeline to do the following steps: brain extraction^77^, movement correction^78^ and co-registration to a study-created brain template^79^, slice timing correction, Gaussian smoothing to 0.8 mm, bandpass filtering 0.01–0.2 Hz using the FSL Toolbox^80^. Data were masked for inclusion of brain voxels common to all mice to prevent bias. A mouse brain atlas (Ekam Solutions, USA) was registered to the study brain template, masked for common brain regions, and then inverse warped back to subject space. Functional adjacency matrices were calculated from the pre-processed, time-series rsfMRI data using 136 brain regions common to all brains to obtain Pearson correlation coefficients that were then Fisher z-transformed. Statistical contrasts were generated between MIR and Control mice and within group between baseline and RAPA intervention using network-based statistics^81^ implemented in GraphVar^82^. Subnetwork edge connectivity was evaluated at *P* < 0.01, two-tailed, using non-parametric permutation testing over 1000 iterations. Seed analysis was conducted by temporal correlation of whole brain data to mean signal extraction from regions of interest at the pre-processed subject-level data, and then at the group-level within the general linear model framework implemented by using FSL Feat, with statistical correction at 2.1z, *P* < 0.01 cluster-based correction^83^. Distribution of brain networks into a community architecture was evaluated by calculation of modularity using 1000 iterations of the Louvain algorithm^84^. Modules derived from this process were then further classified at the nodal level by computing the classification diversity and classification consistency as described previously^85^ as implemented under GraphVar.

Tensor-based deformation analysis: Structural RARE data were converted to nifti image format, brain was extracted from extracranial tissue using FSL BET^86^. All data were used to construct a study-specific, mean deformation template (MDT) images using the ants Multivariate Template Construction script^87^ from Advanced Normalization Tools (ANTs, v.2.3.3)^88^ that consisted of bias field correction and a three-stage coregistration procedure of rigid, affine and non-linear deformable registrations. The raw data were then separately coregistered to the MDT template using a two-stage affine and symmetric diffeomorphic registration under ANTs. The resulting deformation field was then used to derive the Jacobian determinant that provides an indication of the local tissue expansion or atrophy compared to the MDT. All data were masked for a common space to ensure a contribution from all mice to each voxel, following which statistical group differences in voxel-based Jacobian values were computed using non-linear permutation testing implemented under FSL Randomise^89^ under the general linear model framework using cluster-based thresholding set at *z* = 3.1 (*P* < 0.001) and variance smoothing of 0.1 mm. Data were then corrected for multivoxel comparison using false discovery rate correction at *q* = 0.01.

### Immunofluorescence

Brains were flash frozen and cut on a cryostat at 30 μM. Brain sections were post-fixed on slides with 4% paraformaldehyde before being permeabilized with 3% TritonX-100 and immunostained with IBA1 (1:200 Wako 019-19741), NeuN (1:200 ThermoFisher), and Egr1 (1:100 Cell Signaling Technology) primary antibodies, followed by the appropriate Alexa fluorescent secondary antibodies (1:2000 ThermoFisher), and Hoescht counterstain. Positive cells were quantified on a Zeiss AxioImager Z1 fluorescent microscope with Apotome 2.0, using the unbiased optical fractionator approach (StereoInvestigator; MicroBrightField).

### Statistical analyses

Parallel group design was used for most experiments where there are separate MIR and Control offspring groups for every treatment and for different ages (young adult and old adult). Therefore, all univariate data were tested for normality and transformed to Gaussian where required. Behavioral data were analyzed by two-way analysis of variance with repeated-measures, two-tailed paired *t* tests, and mixed effects analysis for multiple comparisons when appropriate (GraphPad v8). Following an overall significant (*P* < 0.05) group and/or interaction effect, Tukey’s and Sidak’s post hoc tests were used to evaluate changes between groups and factors to account for multiple comparisons. Effect size (Cohen’s *d*) was calculated for each result.

### Ethical approval

All experimental procedures were designed to minimize animal suffering and were conducted in strict accordance with the Public Health Service Policy on Humane Care and Use of Laboratory Animals. The protocols were prospectively reviewed and approved by the University of California, Los Angeles (UCLA) Chancellor’s Animal Research Committee (ARC). This manuscript is reported in accordance with the ARRIVE 2.0 guidelines.

### Reporting summary

Further information on research design is available in the Nature Portfolio Reporting Summary linked to this article.

## Supplementary information

Supplementary Information

Reporting Summary

Transparent Peer Review file

## Source data

Source Data

## Data Availability

The data that support the findings of this study are available as follows. Single-nucleus RNA sequencing data have been deposited in the NCBI Gene Expression Omnibus (GEO) under these accession codes [superseries #GSE328222 with subseries GSE328221 (single cell) and subseries GSE328220 (bulk)] and are publicly available as of the date of publication. Raw and preprocessed resting-state functional MRI and structural MRI data have been deposited in [Zenodo.org] under [10.5281/zenodo.19491368]. Univariate blood cytokine, western blot, brain wet weight, behavioral analysis, and slice electrophysiology data in this study have been deposited in [FigShare.com] under [10.6084/m9.figshare.31966476]. Source data underlying all figures and Supplementary Figs. are provided with this paper. Any additional information required to reanalyze the data reported in this paper is available from the corresponding author upon request. Source data are provided with this paper.
