# Supplementary material for: Acute rapamycin treatment reveals distinct mechanisms of dysfunction in a maternal inflammation mouse model
Source: Nat Commun. 2026 Jul 23;17:6386. doi: 10.1038/s41467-026-74958-1 (PMC13396360; doi:10.1038/s41467-026-74958-1)
Supplement: Supplementary file 1 — Supplementary Information [file 41467_2026_74958_MOESM1_ESM.pdf]

Supplemental Methods

Pharmacological Treatments:

All pharmacological treatments were administered to the mice as young adult (P60-90) or old adults (P200-400), apart from the juvenile mice (P25) in the reciprocal social interaction test (see supplemental methods and Fig. S1B). Juvenile mice are commonly used for this test because they are less territorial and more likely to engage in reciprocal behaviors without aggression or dominance behaviors.

RapaBlock: To differentiate central vs. peripheral effects, young adult mice were co-administered rapamycin and the brain-impermeable inhibitor RapaBlock. RapaBlock is a small molecule152,153 that binds FKBP12, and acts to prevent rapamycin from accessing this necessary factor for mTOR inhibition in the peripheral nervous system. Therefore, mice co-administered both rapamycin and RapaBlock would still have mTOR inhibition in the central but not in the peripheral nervous system. RapaBlock was dissolved in DMSO and dissolved in DPBS containing Tween80 and PEG300. Mice were injected simultaneously with 5 mg/kg dose of rapamycin or vehicle control (DMSO) and with 40 mg/kg of RapaBlock or vehicle control, given at the same time via intraperitoneal injection in opposite flanks. Animal behavior was measured in control and MIR offspring groups in treatment conditions with and without rapamycin and with and without RapaBlock. The comparison of the treatment effects of Rapamycin given alone compared to Rapamycin co-administered with RapaBlock indicated whether any rescue effects are due to central nervous system effects of Rapamycin.

S6K1 Inhibitor: The S6K1 inhibitor PF-4708671 (75 mg/kg; formulated in 10% DMSO, 10% Tween-80, 80% water) as described in Huang, et al154., or vehicle was given I.P. 2 hours or 6 hours prior to data collection in young adult MIR and Control offspring.
NOX Inhibitor: The NADPH oxidase (NOX) inhibitor Apocynin (Sigma-Aldrich, cat no. 178385) or vehicle was given I.P. 2 hours prior to data collection or daily for 5 weeks for chronic treatment in young adult MIR and Control offspring.

Blood Cytokine Analysis:
Cytokine abbreviations are as follows: G-CSF=granulocyte colony stimulating factor, IFN-g=interferon gamma, IL-6=interleukin 6, IP-10=interferon gamma induced protein factor, MCP-1=monocyte chemoattractant, MIG= monokine induced by gamma protein interferon factor, VEGF=vascular endothelial growth factor, KC=keratinocyte chemoattractant factor, M-CSF=macrophage colony stimulating factor, GM-CSF=granulocyte macrophage colony stimulating factor, IL-9=interleukin 9 factor, IL-1b=interleukin 1 beta factor, TNF-a=tumor necrosis factor alpha factor, and LIF=leukemia inhibitory factor

Behavioral Testing:
Reciprocal social Interactions: Experimental and age- and sex-matched juvenile (P25) stranger mice were placed together in standard housing cages with clean bedding and both social and non-social behaviors were measured for the number of bouts of each during a 10-minute session as described by Silverman et al., 2010. Social parameters included following (experimental mouse walks slowly behind the stranger mouse, keeping pace), push-crawl (physical contact including pushing the snout or head underneath the partner’s body, squeezing between the partner and the arena wall or floor, and crawling over or under the partner’s body), and sniffing (nose-to-nose or nose-to-anogenital). Non-social parameters include solo arena exploration and self-grooming. The main outcome measures are the number of bouts of each type of behavior the mice performed during the testing session. Social and non-social behavior bouts are compared.

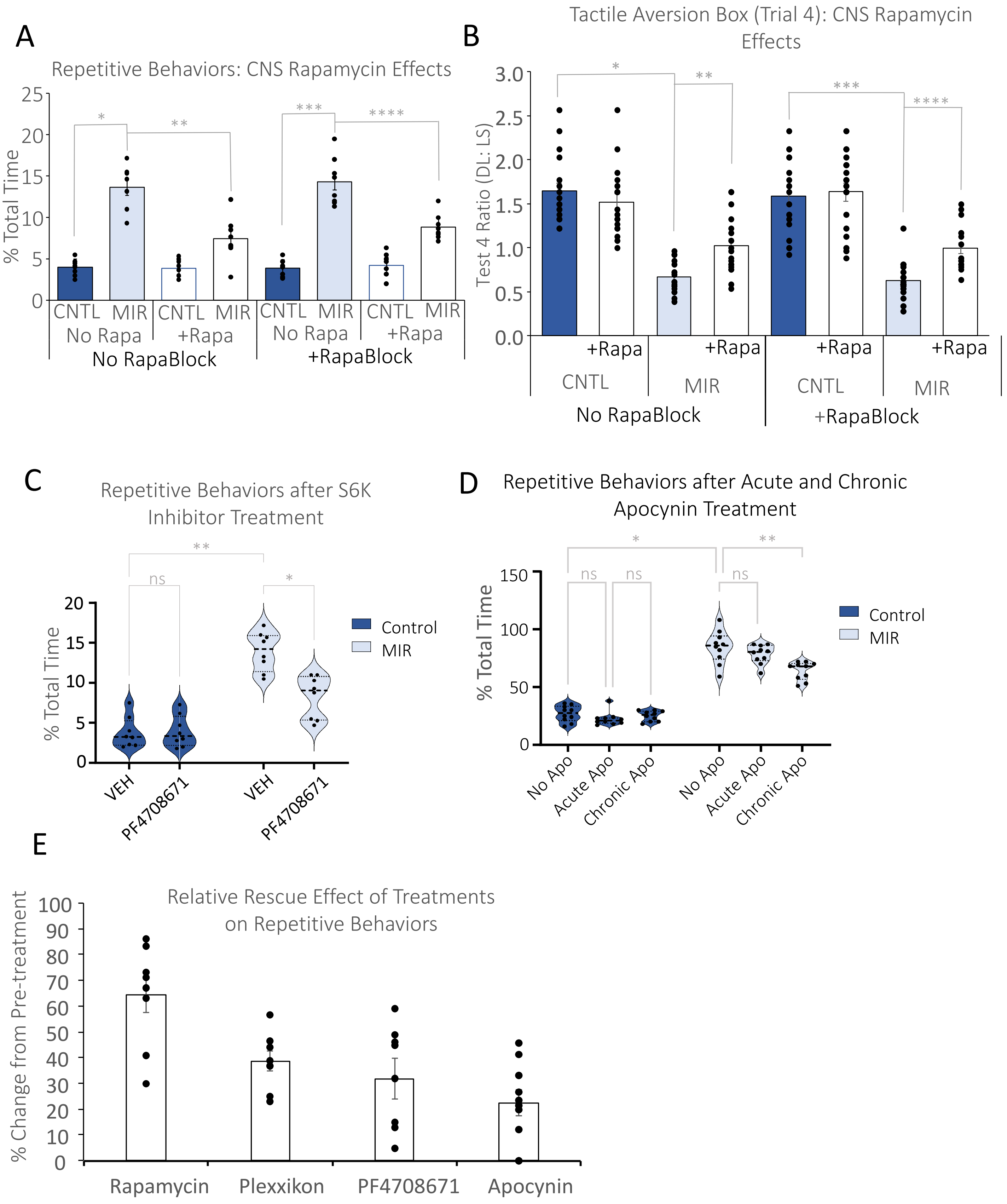

A
Repetitive Behaviors: CNS Rapamycin Effects
% Total Time
0
5
10
15
20
25
*
**
***
****
CNTL
MIR
CNTL
MIR
CNTL
MIR
CNTL
MIR
No Rapa
+Rapa
No Rapa
+Rapa
No RapaBlock
+RapaBlock
B
Tactile Aversion Box (Trial 4): CNS Rapamycin Effects
Test 4 Ratio (DL: LS)
0.0
0.5
1.0
1.5
2.0
2.5
3.0
*
**
***
****
+Rapa
+Rapa
+Rapa
+Rapa
CNTL
MIR
CNTL
MIR
No RapaBlock
+RapaBlock
C
Repetitive Behaviors after S6K Inhibitor Treatment
% Total Time
0
5
10
15
20
**
ns
*
Control
MIR
VEH
PF4708671
VEH
PF4708671
D
Repetitive Behaviors after Acute and Chronic Apocynin Treatment
% Total Time
0
50
100
150
*
**
ns
ns
ns
Control
MIR
No Apo
Acute Apo
Chronic Apo
No Apo
Acute Apo
Chronic Apo
E
Relative Rescue Effect of Treatments on Repetitive Behaviors
% Change from Pre-treatment
0
10
20
30
40
50
60
70
80
90
100
Rapamycin
Plexxikon
PF4708671
Apocynin

Supplemental Figure 1. **RapaBlock co-treatment with Rapamycin confirms the central nervous system action of rapamycin rescue of MIR offspring and S6K inhibition confirms that rescue of repetitive behaviors occurs via the mTOR signaling pathway.** (**A**) A two-way ANOVA analysis for multiple comparisons of repetitive behaviors test in MIR and control offspring treated with vehicle or rapamycin in combination with RapaBlock or RapaBlock vehicle found an overall significant effect of Group (p<0.0001, $F_{(3, 56)}$) but not in RapaBlock treatment (p=0.2398, $F_{(1, 56)}$). Post-hoc analysis (Tukey's) shows that MIR mice had significantly more repetitive behaviors than control offspring when there was no RapaBlock (vehicle, adjusted *p<0.0001, DF=56) or Rapablock given (adjusted **p<0.0001, DF=56) showing the abnormal MIR phenotype is not affected by Rapablock alone. MIR mice treated with rapamycin had significantly reduced repetitive behaviors compared to MIR mice treated with rapa-Vehicle when there was no RapaBlock (vehicle, adjusted ***p<0.0001, DF=56) or there was RapaBlock co-treatment (****p<0.0001, DF=56), suggesting that rapamycin is not acting on the peripheral nervous system in MIR mice. N=8/group, Data shown as mean +/- SEM. (**B**) A two-way ANOVA analysis for multiple comparisons of the ratio of time spent in the dark-rough vs light-smooth (Trial 4) chamber in the tactile light-dark box test shows there is an overall significant group effect (p<0.0001, $F_{(3, 136)}$). Post-hoc analysis (Tukey's) shows that MIR mice spent significantly less time in the dark-rough chamber than control offspring when there was no RapaBlock (vehicle, adjusted *p<0.0001, DF=136) or Rapablock given (adjusted **p<0.0001, DF=136) showing the abnormal MIR phenotype is not affected by Rapablock alone. MIR mice treated with rapamycin spent significantly more time in the dark-rough chamber compared to MIR mice treated with rapa-Vehicle when there was no RapaBlock (vehicle, adjusted ***p<0.0001, DF=136) or there was RapaBlock co-treatment (****p=0.0085, DF=136), also suggesting that rapamycin is not acting on the peripheral nervous system in MIR mice. N=18/group, Data shown as mean +/- SEM (**C**) Two-way ANOVA analysis for multiple comparisons of repetitive behaviors in an open field test in control and MIR offspring treated with the s6-kinase inhibitor PF4708671 or vehicle control shows and overall significant effect of treatment (p=0.0018, $F_{(1, 28)}$), group (p<0.0001, $F_{(1,28)}$), and interaction (p=0.0011, $F_{(1, 28)}$). Post-hoc analysis (Tukey's) shows a significant increase in repetitive behaviors in MIR mice compared to control offspring with vehicle treatment (**p<0.0001, DF=28) and significantly less repetitive behaviors in MIR mice treated with the S6K drug compared to MIR mice treated with vehicle (*p<0.0001, DF=28) but no effect of the drug on control offspring (p=0.8875, DF=28), confirming that downstream targets in the mTOR pathway may also produce some improvement in behavior, n=10/group; (**D**) Two-way ANOVA analysis for multiple comparisons of repetitive behaviors in MIR and control offspring treated with acute and chronic apocynin, an inhibitor of the NADPH oxidase enzyme in the redox-mTOR pathway, shows that there are significant treatment x group (p=0.0006, F(2, 34)), treatment (p=0.0002, F(2, 34)), and group (p<0.0001, F(1, 18)) effects. Post-hoc analysis (Tukey's) shows that MIR mice have significantly more repetitive behaviors compared to control offspring in apocynin (Apo) vehicle-control conditions (adjusted *p<0.0001, DF=13) and MIR mice have less repetitive behaviors in the chronic apocynin group compared to the MIR mice with vehicle-apocynin (adjusted **p=0.0061, DF=9) but not with the acute apocynin group (p=0.1589, DF=9), n=10/group. (**E**) The relative rescue effects of rapamycin, Plexxikon 5622, and PF4708671 are compared demonstrating that the greatest effect on repetitive behaviors is from rapamycin; n=10/group; data shown as mean ± SEM

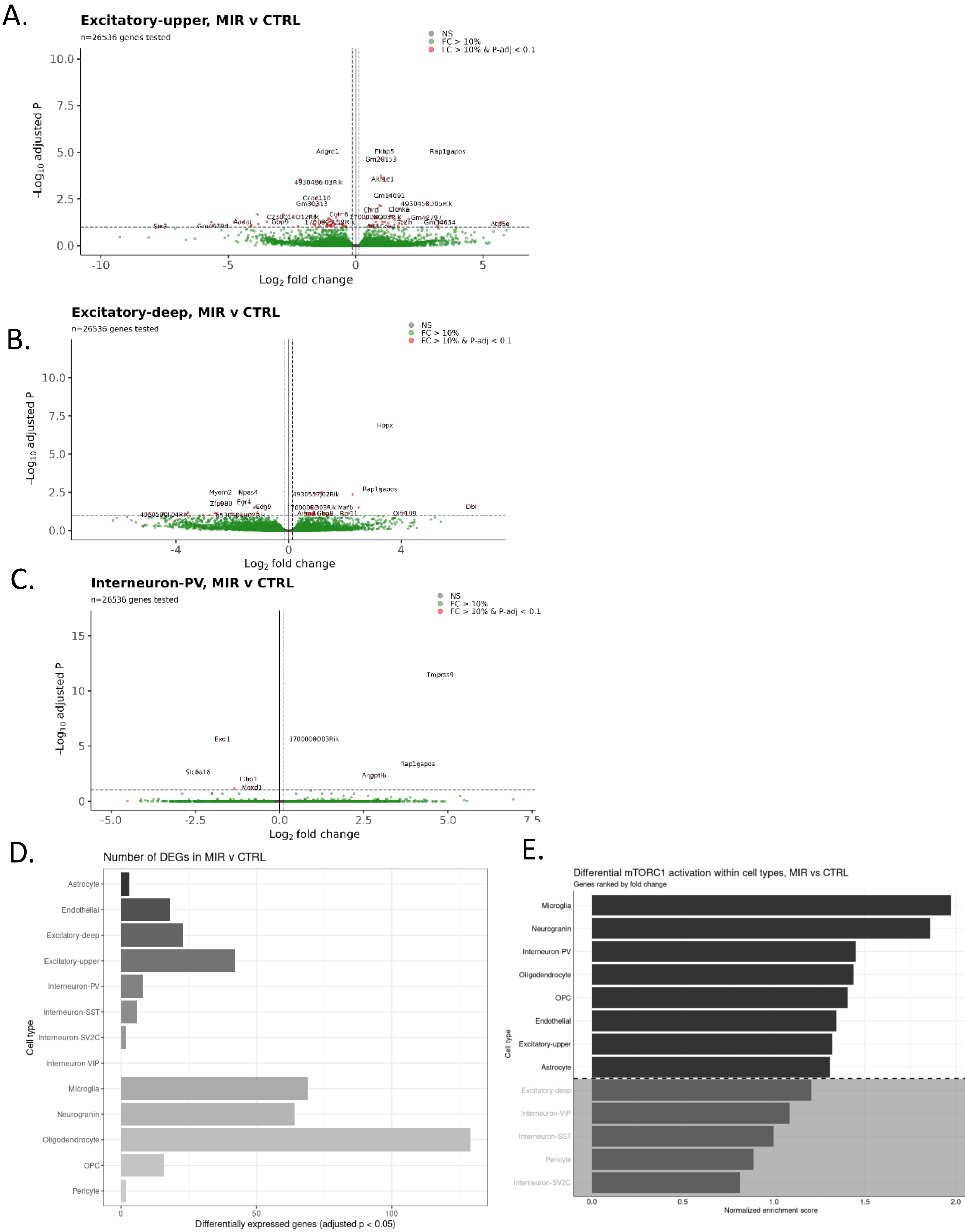
A.
Excitatory-upper, MIR v CTRL
n=26536 genes tested
NS
FC > 10%
FC > 10% & P-adj < 0.1
-Log10 adjusted P
Log2 fold change
Adgrel
Fkbp5
Gm28153
Rap1gapos
Gm14091
Ccdc110
Gm30313
Chrd
Clcnka
Gm34654
Six3
B.
Excitatory-deep, MIR v CTRL
n=26536 genes tested
NS
FC > 10%
FC > 10% & P-adj < 0.1
-Log10 adjusted P
Log2 fold change
Hopx
Myom2
Npas4
Zfp980
Egr4
Cdh9
Rap1gapos
Mafb
Olfr109
Dbi
C.
Interneuron-PV, MIR v CTRL
n=26536 genes tested
NS
FC > 10%
FC > 10% & P-adj < 0.1
-Log10 adjusted P
Log2 fold change
Tmprss9
Exd1
1700008O03Rik
Rap1gapos
Slc6a16
Angptl6
D.
Number of DEGs in MIR v CTRL
Cell type
Astrocyte
Endothelial
Excitatory-deep
Excitatory-upper
Interneuron-PV
Interneuron-SST
Interneuron-SV2C
Interneuron-VIP
Microglia
Neurogranin
Oligodendrocyte
OPC
Pericyte
Differentially expressed genes (adjusted p < 0.05)
E.
Differential mTORC1 activation within cell types, MIR vs CTRL
Genes ranked by fold change
Cell type
Microglia
Neurogranin
Interneuron-PV
Oligodendrocyte
OPC
Endothelial
Excitatory-upper
Astrocyte
Excitatory-deep
Interneuron-VIP
Interneuron-SST
Pericyte
Interneuron-SV2C
Normalized enrichment score

Supplemental Figure 2. **Cell sequencing from sensorimotor cortex microdissections from MIR and control adult offspring.** Clustering of single-cell RNA seq data from sensorimotor cortex microdissections as volcano plots of differentially expressed genes in MIR offspring compared to Controls in (**A**) upper layer and (**B**) deep layer excitatory neurons and in (**C**) parvalbumin (PV) inhibitory interneurons; (**D**) The number of genes enriched in MIR mice by cell type; (**E**) Relative enrichment (fold change) of mTOR pathway regulated genes significantly enriched in MIR mice by cell type indicates that MIR mice have an increase in these genes in microglia, neurogranin neurons, PV+ interneurons, oligodendrocytes, oligodendrocyte precursor cells (OPC), endothelial cells, upper layer excitatory neurons, and astrocytes compared to control offspring. N=5 MIR, N=6 Control, for all sequencing data a false discovery rate (FDR) < 0.05, LogFC > 0.5, and $p < 0.05$ threshold was used.

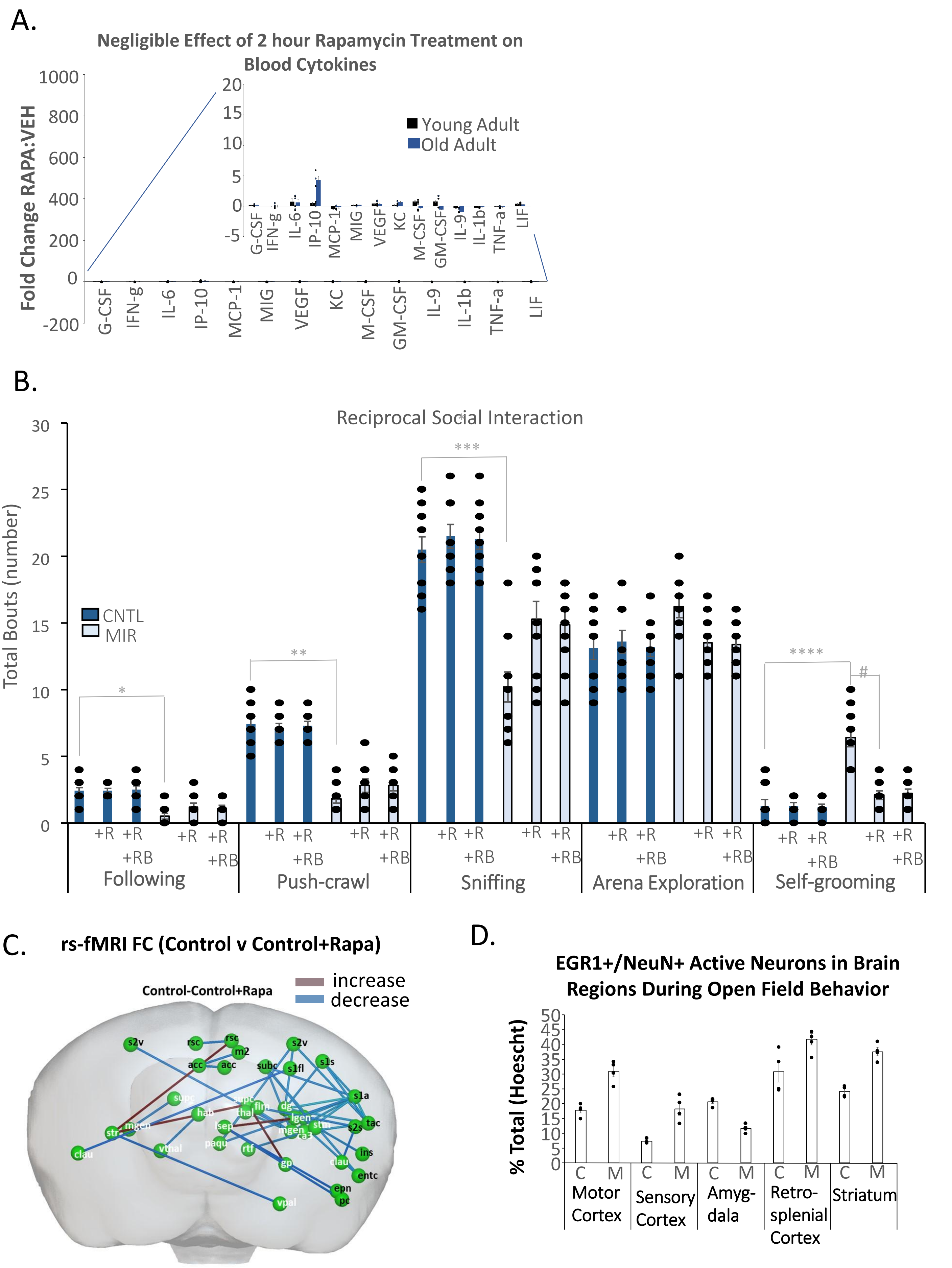
A.
Negligible Effect of 2 hour Rapamycin Treatment on Blood Cytokines
Fold Change RAPA:VEH
1000
800
600
400
200
0
-200
20
15
10
5
0
-5
Young Adult
Old Adult
G-CSF
IFN-g
IL-6
IP-10
MCP-1
MIG
VEGF
KC
M-CSF
GM-CSF
IL-9
IL-1b
TNF-a
LIF
B.
Reciprocal Social Interaction
Total Bouts (number)
30
25
20
15
10
5
0
CNTL
MIR
*
**
***
****
#
+R
+RB
Following
Push-crawl
Sniffing
Arena Exploration
Self-grooming
C.
rs-fMRI FC (Control v Control+Rapa)
Control-Control+Rapa
increase
decrease
D.
EGR1+/NeuN+ Active Neurons in Brain Regions During Open Field Behavior
% Total (Hoescht)
50
45
40
35
30
25
20
15
10
5
0
C
M
Motor Cortex
Sensory Cortex
Amyg-dala
Retro-splenial Cortex
Striatum

Supplemental Figure 3. Acute Rapamycin Doesn't Decrease Inflammatory Cytokines in MIR Mice, MIR mice have deficits in some reciprocal social interaction behaviors that is improved by acute rapamycin and is not affected by RapaBlock, Rapamycin-induced Functional Connectivity Changes in Resting State fMRI in Control Offspring, and Immunohistochemistry Validation of Increased Neuron Activity in Brain Regions Indicated by fMRI Results

(**A**) Blood cytokines are unchanged by 2-hour Rapamycin treatment in adult MIR mice (all n.s.), see Figure 1 legend for cytokine abbreviations and full names, N=4/group; (**B**) A two-way ANOVA analysis for multiple comparisons of social approach behaviors in MIR and control offspring treated with rapamycin or vehicle control in combination with RapaBlock or RapaBlock-vehicle found significant behavior x group (p<0.0001, F(11, 115)), behavior (p<0.0001, F(2, 115)) and group (p<0.0001, F(5, 54)) effects. Post-hoc analysis (Tukey's) shows significant deficits in MIR mice treated with vehicle-rapamycin and vehicle-RapaBlock compared to control offspring treated with vehicle-rapamycin and vehicle-RapaBlock for following behavior (adjusted *p=0.0005, DF=18), push-crawl behavior (**p<0.0001, DF=16), and sniffing behavior (adjusted ***p<0.0001, DF=17). There was no significant difference between MIR offspring receiving rapamycin and no RapaBlock compared to MIR offspring receiving rapamycin and RapaBlock for following behavior (adjusted #p=0.9998, DF=17), push-crawl behavior (adjusted p>0.9999, DF=17), and sniffing behavior (adjusted p=0.9999, DF=17). There was a similar but opposite effect with self-grooming during the test with MIR offspring treated with rapamycin-vehicle and RapaBlock-vehicle had significantly increased behavior compared to control offspring treated with rapamycin-vehicle and RapaBlock-vehicle (adjusted ****p=0.0001, DF=16), a significant difference between MIR offspring treated with rapamycin-vehicle and RapaBlock-vehicle and MIR mice treated with rapamycin and RapaBlock-vehicle (adjusted p=0.0007, DF=13) but there was no significant difference in grooming for MIR offspring treated with rapamycin and RapaBock-vehicle and rapamycin and RapaBlock (adjusted p>0.9999, DF=17), demonstrating no RapaBlock effect on rapamycin treatment which suggests that the effects of rapamycin are not via the peripheral nervous system, N=8/group/treatment; (**C**) The changes in FC in control mice after acute rapamycin treatment were small compared to MIR mice (Fig. 6C) and were mostly decreases (blue lines) between cortical and subcortical regions with few increases (red lines) between retrosplenial cortex and basal ganglia structures, N=16/group; (**D**) A two-way ANOVA analysis for multiple comparisons of the number of NeuN+ neurons that express the immediate early gene protein EGF1 (as a percentage of total Hoescht+ cells) shows significant overall effects in brain region x group (p=0.0005, $F_{(2, 12)}$), brain region (p<0.0001, $F_{(2, 12)}$), and group (p=0.0003, $F_{(1, 6)}$). Post-hoc analysis (Tukey's) shows that there are significant increases in these cells in the brains from MIR mice compared to control offspring in the motor cortex (adjusted p=0.0024, DF =5), the sensory-motor cortex (adjusted p=0.0126, DF=3), and striatum (adjusted p=0.0005, DF=5) and a significant decrease in the amygdala (adjusted p<0.0001, DF=6). There was no significant difference in the retrosplenial cortex (adjusted p=0.0587, DF=5), N=4/group; All data mean +/- SEM.

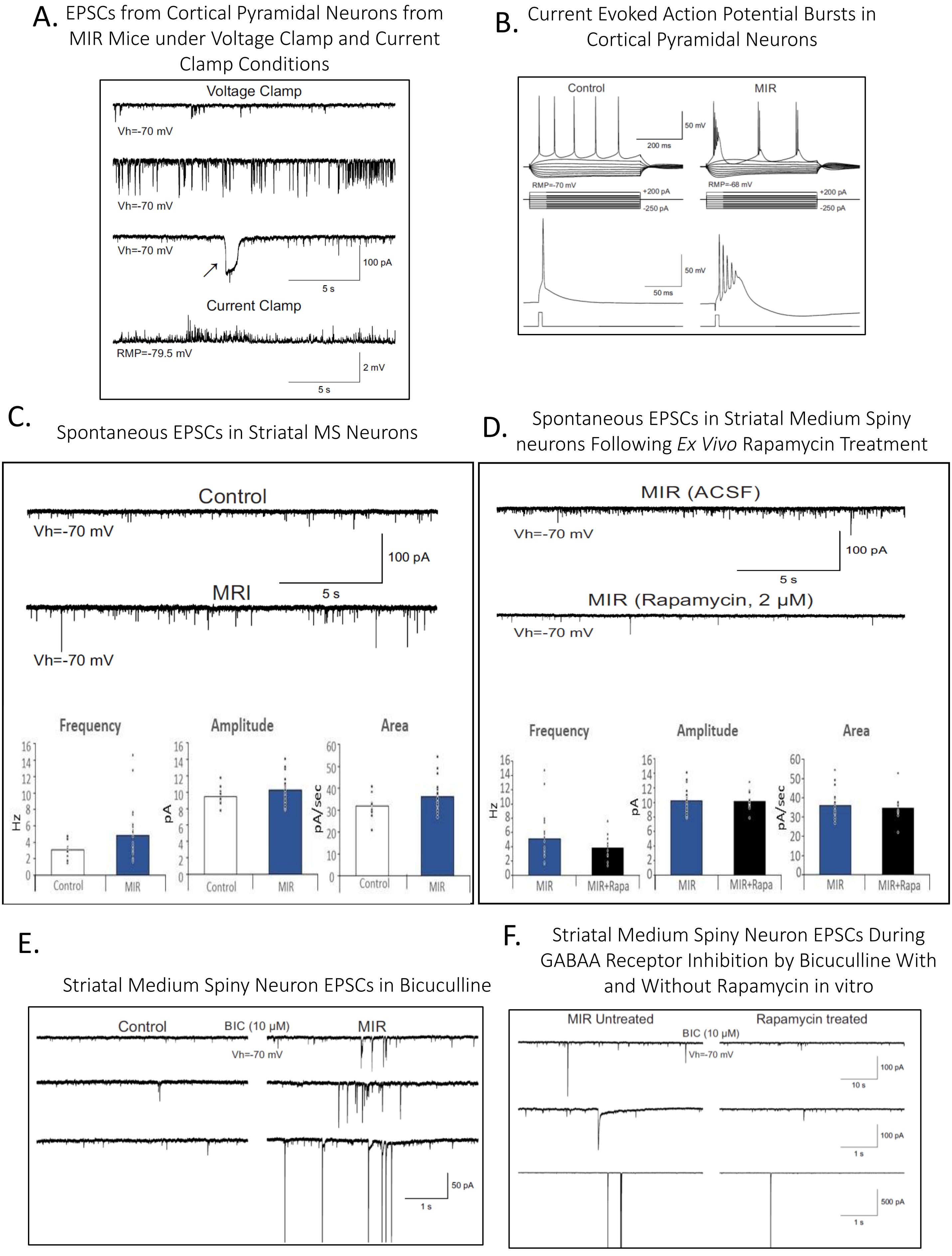
A. EPSCs from Cortical Pyramidal Neurons from MIR Mice under Voltage Clamp and Current Clamp Conditions
Voltage Clamp
Vh=-70 mV
Vh=-70 mV
Vh=-70 mV
100 pA
5 s
Current Clamp
RMP=-79.5 mV
2 mV
5 s
B. Current Evoked Action Potential Bursts in Cortical Pyramidal Neurons
Control
MIR
50 mV
200 ms
RMP=-70 mV
RMP=-68 mV
+200 pA
-250 pA
+200 pA
-250 pA
50 mV
50 ms
C. Spontaneous EPSCs in Striatal MS Neurons
Control
Vh=-70 mV
100 pA
5 s
MRI
Vh=-70 mV
Frequency
Amplitude
Area
Hz
pA
pA/sec
Control
MIR
Control
MIR
Control
MIR
D. Spontaneous EPSCs in Striatal Medium Spiny neurons Following Ex Vivo Rapamycin Treatment
MIR (ACSF)
Vh=-70 mV
100 pA
5 s
MIR (Rapamycin, 2 μM)
Vh=-70 mV
Frequency
Amplitude
Area
Hz
pA
pA/sec
MIR
MIR+Rapa
MIR
MIR+Rapa
MIR
MIR+Rapa
E. Striatal Medium Spiny Neuron EPSCs in Bicuculline
Control
BIC (10 μM)
MIR
Vh=-70 mV
50 pA
1 s
F. Striatal Medium Spiny Neuron EPSCs During GABAA Receptor Inhibition by Bicuculline With and Without Rapamycin in vitro
MIR Untreated
Rapamycin treated
BIC (10 μM)
Vh=-70 mV
100 pA
10 s
100 pA
1 s
500 pA
1 s

Supplemental Figure 4. Cortical Somatosensory Pyramidal Neurons Display Multiple Signs of Hyper-Excitability in MIR Brains and Medium Spiny Neurons in the Striatum Show Trends Towards Hyper-excitability that is Reduced by Acute Rapamycin Treatment. (**A**) Example of excitatory post-synaptic current activity in cortical pyramidal neurons under voltage clamp and current clamp conditions; (**B**) Example of action potential bursting from cortical pyramidal neurons under current clamp conditions; (**C**) Traces and quantification of the frequency, amplitude, and area of spontaneous excitatory postsynaptic currents in striatal medium spiny neurons from adult MIR and control offspring show a trend for increases in MIR mice compared to control but do not reach significance; (**D**) Traces and quantification of the frequency, amplitude, and area of spontaneous excitatory postsynaptic currents in striatal medium spiny neurons from adult MIR mice treated acutely with rapamycin ex vivo (2uM) compared to controls shows a trend for decreased spiking frequency that does not reach significance; (**E**) Traces showing treatment with Bicuculline, a competitive antagonist of GABA A receptors, increases excitatory post-synaptic currents in medium spiny neurons in the striatum, (**F**) Traces showing acute *ex vivo* rapamycin treatment given simultaneously with Bicuculline prevents the increase in medium spiny neurons EPSCs; n=24/group

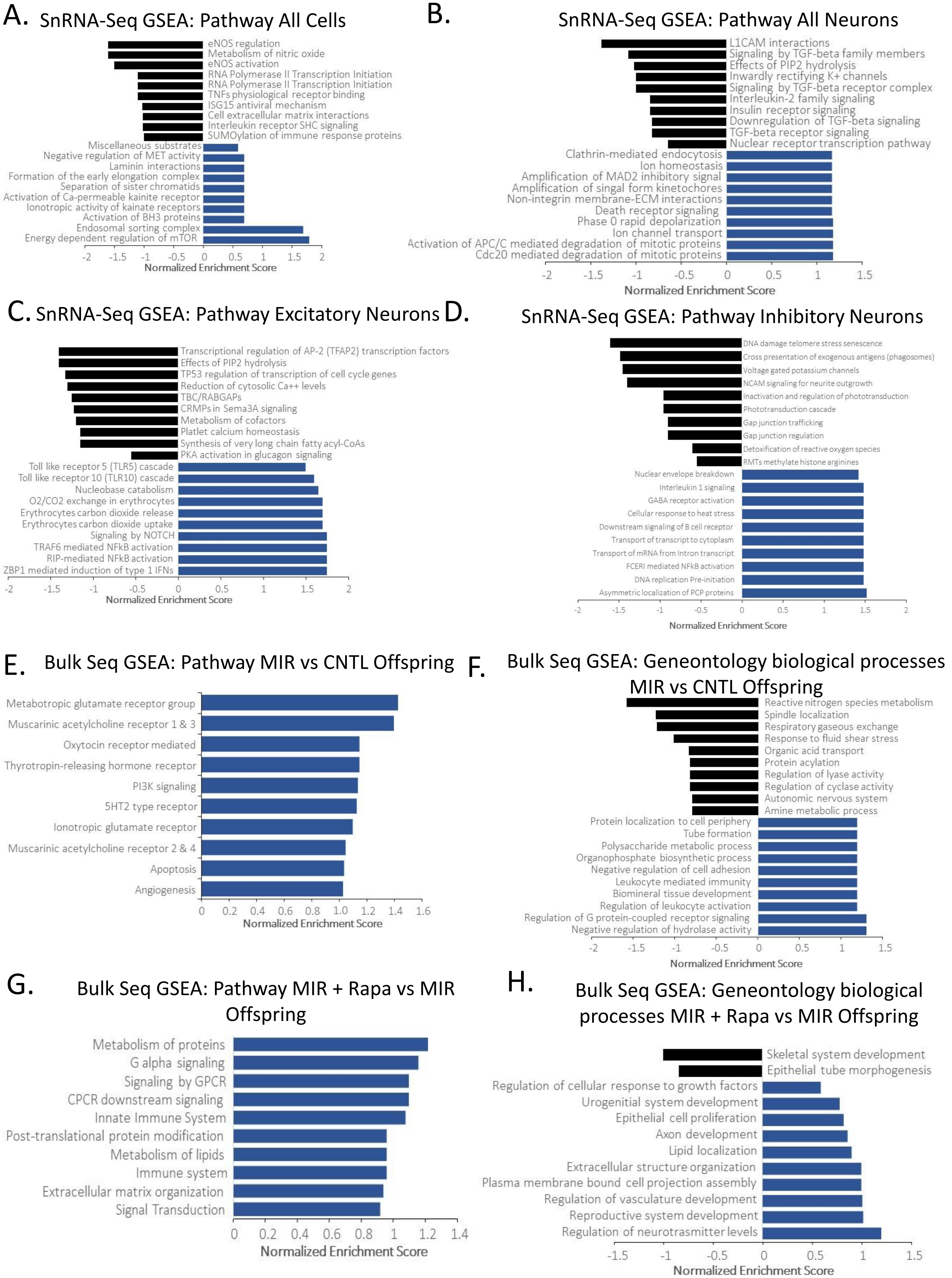
A. SnRNA-Seq GSEA: Pathway All Cells
eNOS regulation
Metabolism of nitric oxide
eNOS activation
RNA Polymerase II Transcription Initiation
RNA Polymerase II Transcription Initiation
TNFs physiological receptor binding
ISG15 antiviral mechanism
Cell extracellular matrix interactions
Interleukin receptor SHC signaling
SUMOylation of immune response proteins
Miscellaneous substrates
Negative regulation of MET activity
Laminin interactions
Formation of the early elongation complex
Separation of sister chromatids
Activation of Ca-permeable kainite receptor
Ionotropic activity of kainate receptors
Activation of BH3 proteins
Endosomal sorting complex
Energy dependent regulation of mTOR
Normalized Enrichment Score
B. SnRNA-Seq GSEA: Pathway All Neurons
L1CAM interactions
Signaling by TGF-beta family members
Effects of PIP2 hydrolysis
Inwardly rectifying K+ channels
Signaling by TGF-beta receptor complex
Interleukin-2 family signaling
Insulin receptor signaling
Downregulation of TGF-beta signaling
TGF-beta receptor signaling
Nuclear receptor transcription pathway
Clathrin-mediated endocytosis
Ion homeostasis
Amplification of MAD2 inhibitory signal
Amplification of singal form kinetochores
Non-integrin membrane-ECM interactions
Death receptor signaling
Phase 0 rapid depolarization
Ion channel transport
Activation of APC/C mediated degradation of mitotic proteins
Cdc20 mediated degradation of mitotic proteins
Normalized Enrichment Score
C. SnRNA-Seq GSEA: Pathway Excitatory Neurons
Transcriptional regulation of AP-2 (TFAP2) transcription factors
Effects of PIP2 hydrolysis
TP53 regulation of transcription of cell cycle genes
Reduction of cytosolic Ca++ levels
TBC/RABGAPs
CRMPs in Sema3A signaling
Metabolism of cofactors
Platlet calcium homeostasis
Synthesis of very long chain fatty acyl-CoAs
PKA activation in glucagon signaling
Toll like receptor 5 (TLR5) cascade
Toll like receptor 10 (TLR10) cascade
Nucleobase catabolism
O2/CO2 exchange in erythrocytes
Erythrocytes carbon dioxide release
Erythrocytes carbon dioxide uptake
Signaling by NOTCH
TRAF6 mediated NFkB activation
RIP-mediated NFkB activation
ZBP1 mediated induction of type 1 IFNs
Normalized Enrichment Score
D. SnRNA-Seq GSEA: Pathway Inhibitory Neurons
DNA damage telomere stress senescence
Cross presentation of exogenous antigens (phagosomes)
Voltage gated potassium channels
NCAM signaling for neurite outgrowth
Inactivation and regulation of phototransduction
Phototransduction cascade
Gap junction trafficking
Gap junction regulation
Detoxification of reactive oxygen species
RMTs methylate histone arginines
Nuclear envelope breakdown
Interleukin 1 signaling
GABA receptor activation
Cellular response to heat stress
Downstream signaling of B cell receptor
Transport of transcript to cytoplasm
Transport of mRNA from Intron transcript
FCERI mediated NFkB activation
DNA replication Pre-initiation
Asymmetric localization of PCP proteins
Normalized Enrichment Score
E. Bulk Seq GSEA: Pathway MIR vs CNTL Offspring
Metabotropic glutamate receptor group
Muscarinic acetylcholine receptor 1 & 3
Oxytocin receptor mediated
Thyrotropin-releasing hormone receptor
PI3K signaling
5HT2 type receptor
Ionotropic glutamate receptor
Muscarinic acetylcholine receptor 2 & 4
Apoptosis
Angiogenesis
Normalized Enrichment Score
F. Bulk Seq GSEA: Geneontology biological processes MIR vs CNTL Offspring
Reactive nitrogen species metabolism
Spindle localization
Respiratory gaseous exchange
Response to fluid shear stress
Organic acid transport
Protein acylation
Regulation of lyase activity
Regulation of cyclase activity
Autonomic nervous system
Amine metabolic process
Protein localization to cell periphery
Tube formation
Polysaccharide metabolic process
Organophosphate biosynthetic process
Negative regulation of cell adhesion
Leukocyte mediated immunity
Biomineral tissue development
Regulation of leukocyte activation
Regulation of G protein-coupled receptor signaling
Negative regulation of hydrolase activity
Normalized Enrichment Score
G. Bulk Seq GSEA: Pathway MIR + Rapa vs MIR Offspring
Metabolism of proteins
G alpha signaling
Signaling by GPCR
CPCR downstream signaling
Innate Immune System
Post-translational protein modification
Metabolism of lipids
Immune system
Extracellular matrix organization
Signal Transduction
Normalized Enrichment Score
H. Bulk Seq GSEA: Geneontology biological processes MIR + Rapa vs MIR Offspring
Skeletal system development
Epithelial tube morphogenesis
Regulation of cellular response to growth factors
Urogenitial system development
Epithelial cell proliferation
Axon development
Lipid localization
Extracellular structure organization
Plasma membrane bound cell projection assembly
Regulation of vasculature development
Reproductive system development
Regulation of neurotrasmitter levels
Normalized Enrichment Score

Supplemental Figure 5. Gene Set Enrichment Analysis of snRNA seq and Bulk sequencing of Genes that Are Significantly Differentially Expressed in MIR Offspring.

(A) Gene set enrichment analysis (GSEA) of pseudo-bulk expression from single-nucleus RNA sequencing in cellular pathways for all cell types in MIR mice relative to control offspring; (B) GSEA of pathways enriched in MIR mice in all neurons; (C) GSEA of pathways enriched in MIR mice in excitatory neurons; (D) GSEA of pathways enriched in MIR mice in inhibitory neurons; (E) Gene set enrichment analysis (GSEA) of pathways enriched in MIR mice compared to control offspring from bulk sequencing; (F) Geneontology of biological processes enriched in MIR mice compared to control offspring; (G) GSEA of pathways differentially expressed in MIR mice treated with rapamycin compared to vehicle treated MIR mice ; (H) Geneontology of biological processes differentially expressed in MIR mice treated with rapamycin compared to vehicle-treated MIR mice; expression and significance criteria used for all are LogFC>1, P≤0.05, FDR ≤0.1, snRNA seq n=5 MIR, n=6 control; Bulk seq n=8/group/treatment

ASD Associated Genes					
MIR vs CNTL Offspring		CNTL Rapa vs CNTL Veh		MIR Rapa vs MIR Veh	
**UP in MIR**	**Down in MIR**	**UP in CR**	**Down in CR**	**Up in MR**	**Down in MR**
TEK (Tie2)	ACE	CDH22	TEK (Tie2)	PCDHA7	TEK (Tie2)
PLAUR (CD87)	CNTNAP3	PLAUR (CD87)	NTNG1	TEKT4	PLAUR (CD87)
ID1	MFRP	PPP1R1B	MFRP		ID1
HOMER1	PCDH11X	ADA	CSMD3		SIK1
ASPM	CHD7	MEGF11	GPR37		CDKN1A (p21)
CDKN1A (p21)	TEKT4		PCDH15		
IQGAP3	NEUROD1		DCC		
KIF14	GRIN2C		KHDRBS2		
MAST3	SLC35D3				

Supplemental Table 1. (**F**) Bulk sequencing analysis of autism-associated genes that are significantly enriched in MIR mice relative to control offspring; All data use (LogFC >1, P<0.05, FDR<0.1) for abundance and significance criteria.

MIR v Control DEGs Reversed by Rapamycin	
**Decreased by Rapa**	**Increased by Rapa**
TEK (Tie 2)	HIF3A
PLAUR (uPAR, CD87)	PLIN4
NESTIN	TEKT4
GEM	CHD7
ID1	CNTNAP3
TIPARP	
APOLD1	
CDKN1A (p21)	

Supplemental Table 2. Bulk sequencing analysis of differentially expressed genes that were enriched in MIR offspring relative to control offspring that are rescued (reversed) by acute rapamycin treatment. All data use (LogFC >1, P<0.05, FDR<0.1) for abundance and significance criteria.

Litter	Group	**Litter Testing**	Figure	Table
1	CNTL	Blood Cytokines Old/Microglia IHC/+/-Rapa	1B, 1D	
2	CNTL	Blood Cytokines Old/Microglia IHC/+/-Rapa	1B, 1D	
3	MIR	Blood Cytokines Old/Microglia IHC/+/-Rapa	1B, 1D	
4	MIR	Blood Cytokines Old/Microglia IHC/+/-Rapa	1B, 1D	
5	CNTL	repetitive behaviors Old +/-Rapa/Longevity of Rapa/Chronic Rapa	3C-E	
6	CNTL	repetitive behaviors Old +/-Rapa/Longevity of Rapa/Chronic Rapa	3C-E	
7	CNTL	repetitive behaviors Old +/-Rapa/Longevity of Rapa/Chronic Rapa	3C-E	
8	MIR	repetitive behaviors Old +/-Rapa/Longevity of Rapa/Chronic Rapa	3C-E	
9	MIR	repetitive behaviors Old +/-Rapa/Longevity of Rapa/Chronic Rapa	3C-E	
10	MIR	repetitive behaviors Old +/-Rapa/Longevity of Rapa/Chronic Rapa	3C-E	
11	CNTL	repetitive behaviors Old/light-dark box/Von Frey/PPI/MRI+/-Rapa/Egfr1 IHC	4A-F, 2B-C, 7A-F, S1	
12	CNTL	repetitive behaviors Old/light-dark box/Von Frey/PPI/MRI+/-Rapa/Egfr1 IHC	4A-F, 2B-C, 7A-F, S1	
13	CNTL	repetitive behaviors Old/light-dark box/Von Frey/PPI/MRI+/-Rapa/Egfr1 IHC	4A-F, 2B-C, 7A-F, S1	
14	CNTL	repetitive behaviors Old/light-dark box/Von Frey/PPI/MRI+/-Rapa/Egfr1 IHC	4A-F, 2B-C, 7A-F, S1	
15	MIR	repetitive behaviors Old/light-dark box/Von Frey/PPI/MRI+/-Rapa/Egfr1 IHC	4A-F, 2B-C, 7A-F, S1	
16	MIR	repetitive behaviors Old/light-dark box/Von Frey/PPI/MRI+/-Rapa/Egfr1 IHC	4A-F, 2B-C, 7A-F, S1	
17	MIR	repetitive behaviors Old/light-dark box/Von Frey/PPI/MRI+/-Rapa/Egfr1 IHC	4A-F, 2B-C, 7A-F, S1	
18	MIR	repetitive behaviors Old/light-dark box/Von Frey/PPI/MRI+/-Rapa/Egfr1 IHC	4A-F, 2B-C, 7A-F, S1	
19	CNTL	repetitive behaviors Old/Von Frey/+/-PLex5622/e-Phys slice recording +/-Rapa	1F-G, 6A-D, S2	T1
20	CNTL	repetitive behaviors Old/Von Frey/+/-PLex5622/e-Phys slice recording +/-Rapa	1F-G, 6A-D, S2	T1
21	MIR	repetitive behaviors Old/Von Frey/+/-PLex5622/e-Phys slice recording +/-Rapa	1F-G, 6A-D, S2	T1
22	MIR	repetitive behaviors Old/Von Frey/+/-PLex5622/e-Phys slice recording +/-Rapa	1F-G, 6A-D, S2	T1
23	CNTL	repetitive behaviors Old/Von Frey/+/-PLex5622/e-Phys slice recording +/-Rapa	1F-G, 6A-D, S2	T1
24	CNTL	repetitive behaviors Old/Von Frey/+/-PLex5622/e-Phys slice recording +/-Rapa	1F-G, 6A-D, S2	T1
25	MIR	repetitive behaviors Old/Von Frey/+/-PLex5622/e-Phys slice recording +/-Rapa	1F-G, 6A-D, S2	T1
26	MIR	repetitive behaviors Old/Von Frey/+/-PLex5622/e-Phys slice recording +/-Rapa	1F-G, 6A-D, S2	T1
27	CNTL	repetitive behaviors Young/Von Frey/PPI/light-dark box/+/-Rapa+/-PF470	3C, 4A-F,5C, 5E	
28	CNTL	repetitive behaviors Young/Von Frey/PPI/light-dark box/+/-Rapa+/-PF470	3C, 4A-F,5C, 5E	
29	CNTL	repetitive behaviors Young/Von Frey/PPI/light-dark box/+/-Rapa+/-PF470	3C, 4A-F,5C, 5E	
30	MIR	repetitive behaviors Young/Von Frey/PPI/light-dark box/+/-Rapa+/-PF470	3C, 4A-F,5C, 5E	
31	MIR	repetitive behaviors Young/Von Frey/PPI/light-dark box/+/-Rapa+/-PF470	3C, 4A-F,5C, 5E	
32	MIR	repetitive behaviors Young/Von Frey/PPI/light-dark box/+/-Rapa+/-PF470	3C, 4A-F,5C, 5E	
33	CNTL	repetitive behaviors Young/Von Frey/+/-Plex5622/Sequencing +/- Rapa/PTZ seizure	1F-G, 8A-E, S3	T2-6, ST1
34	CNTL	repetitive behaviors Young/Von Frey/+/-Plex5622/Sequencing +/- Rapa/PTZ seizure	1F-G, 8A-E, S3	T2-6, ST1
35	CNTL	repetitive behaviors Young/Von Frey/+/-Plex5622/Sequencing +/- Rapa/PTZ seizure	1F-G, 8A-E, S3	T2-6, ST1
36	MIR	repetitive behaviors Young/Von Frey/+/-Plex5622/Sequencing +/- Rapa/PTZ seizure	1F-G, 8A-E, S3	T2-6, ST1
37	MIR	repetitive behaviors Young/Von Frey/+/-Plex5622/Sequencing +/- Rapa/PTZ seizure	1F-G, 8A-E, S3	T2-6, ST1
38	MIR	repetitive behaviors Young/Von Frey/+/-Plex5622/Sequencing +/- Rapa/PTZ seizure	1F-G, 8A-E, S3	T2-6, ST1
39	CNTL	repetitive behaviors/+/-Rapa/+/-Rapablock	5A-B	
40	CNTL	repetitive behaviors/+/-Rapa/+/-Rapablock	5A-B	
41	MIR	repetitive behaviors/+/-Rapa/+/-Rapablock	5A-B	
42	MIR	repetitive behaviors/+/-Rapa/+/-Rapablock	5A-B	
43	CNTL	Brain Wet Weight	2A	
44	CNTL	Brain Wet Weight	2A	
45	MIR	Brain Wet Weight	2A	
46	MIR	Brain Wet Weight	2A	
47	CNTL	PPI/light-dark box/+/-Rapa/+/-Rapablock/+/-Apo/Reciprocal Social Interaction+/-Rapa/+/-Rapablock	1E, 3B, 5A-B, 5D, 5E, S1	
48	CNTL	PPI/light-dark box/+/-Rapa/+/-Rapablock/+/-Apo/Reciprocal Social Interaction+/-Rapa/+/-Rapablock	1E, 3B, 5A-B, 5D, 5E, S1	
49	CNTL	PPI/light-dark box/+/-Rapa/+/-Rapablock/+/-Apo/Reciprocal Social Interaction+/-Rapa/+/-Rapablock	1E, 3B, 5A-B, 5D, 5E, S1	
50	MIR	PPI/light-dark box/+/-Rapa/+/-Rapablock/+/-Apo/Reciprocal Social Interaction+/-Rapa/+/-Rapablock	1E, 3B, 5A-B, 5D, 5E, S1	
51	MIR	PPI/light-dark box/+/-Rapa/+/-Rapablock/+/-Apo/Reciprocal Social Interaction+/-Rapa/+/-Rapablock	1E, 3B, 5A-B, 5D, 5E, S1	
52	MIR	PPI/light-dark box/+/-Rapa/+/-Rapablock/+/-Apo/Reciprocal Social Interaction+/-Rapa/+/-Rapablock	1E, 3B, 5A-B, 5D, 5E, S1	
Dams	Group	Dam Testing	Figure	
1-4	CNTL	Blood Cytokines Pregnant Dams	1C	
5-6	MIR	Blood Cytokines Pregnant Dams	1C	

Supplemental Table 3. Litters Generated for Experiments. Fifty-eight independent litters were generated over several years for the experiments performed in this manuscript. The experiments and the figures the mice contributed to by group (MIR or CNTL) are indicated. Abbreviations are as follows: MIR = Maternal Inflammatory Response Offspring, MRI = Magnetic Resonance Imaging, e-Phys = electrophsilogical CNTL = Vehicle Control Offspring, Rapa = Rapamycin, PPI = pre-pulse inhibition test, APO = apocynin; PTZ = pentylenetetrazol, IHC = immunohistochemistry.

Quantified Seizure Response by Mouse

	PTZ dosage (mg/kg)									
seizure level mouse is in	10		20		30		40		40 + Rapamycin	
mouse	**CNTL**	**MIR**	**CNTL**	**MIR**	**CNTL**	**MIR**	**CNTL**	**MIR**	**CNTL**	**MIR**
m1	0	0	0	1	1	1	1	1	1	1
m2	0	0	0	0	0	1	1	2	0	1
m3	0	0	0	0	0	1	0	2	0	1
m4	0	0	0	0	0	2	0	3	0	2
m5	0	0	0	0	0	0	0	3	0	2
m6	0	0	0	0	0	0	0	3	0	2
m7	0	0	0	0	0	0	0	4	0	2
m8	0	0	0	0	0	0	0	4	0	3

Supplemental Table 4. Quantified Seizure Response by Mouse. The highest seizure level reached on the 1-6 scale (see methods for descriptors) was recorded for each mouse at each PTZ dosage from 10-40 mg/kg) and at 40 mg/kg with 2 hour rapamycin treatment prior to PTZ administration. A 2-way ANOVA mixed model analysis for multiple comparisons showed significant treatment x group ($p<0.0001$, DF=4), treatment ($p<0.0001$, DF=4), and group ($p<0.0001$, DF=1) effects. Posthoc Tukey's multiple comparisons show that at 40mg/kg there is a significant difference between MIR and Control offspring ($p=0.0001$, DF=9.692) and between MIR 40mg/kg PTZ and MIR 40mg/kg PTZ + rapamycin ($p=0.0303$, DF=7). N=8/group/treatment, A two-way ANOVA analysis for multiple comparisons with post-hoc Tukey's analysis of group effects was used.
