# Supplementary material for: Acute rapamycin treatment reveals distinct mechanisms of dysfunction in a maternal inflammation mouse model
Source: Nat Commun. 2026 Jul 23;17:6386. doi: 10.1038/s41467-026-74958-1 (PMC13396360; doi:10.1038/s41467-026-74958-1)
Supplement: Supplementary file 2 — Reporting Summary [file 41467_2026_74958_MOESM2_ESM.pdf]

nature portfolio

Corresponding author(s): Harley Kornblum and Neil Harris

Last updated by author(s): May 6, 2026

Reporting Summary

Nature Portfolio wishes to improve the reproducibility of the work that we publish. This form provides structure for consistency and transparency in reporting. For further information on Nature Portfolio policies, see our Editorial Policies and the Editorial Policy Checklist.

Statistics

For all statistical analyses, confirm that the following items are present in the figure legend, table legend, main text, or Methods section.

n/a	Confirmed	
☐	☒	The exact sample size (*n*) for each experimental group/condition, given as a discrete number and unit of measurement
☐	☒	A statement on whether measurements were taken from distinct samples or whether the same sample was measured repeatedly
☐	☒	The statistical test(s) used AND whether they are one- or two-sided<br>*Only common tests should be described solely by name; describe more complex techniques in the Methods section.*
☐	☒	A description of all covariates tested
☐	☒	A description of any assumptions or corrections, such as tests of normality and adjustment for multiple comparisons
☐	☒	A full description of the statistical parameters including central tendency (e.g. means) or other basic estimates (e.g. regression coefficient) AND variation (e.g. standard deviation) or associated estimates of uncertainty (e.g. confidence intervals)
☐	☒	For null hypothesis testing, the test statistic (e.g. *F*, *t*, *r*) with confidence intervals, effect sizes, degrees of freedom and *P* value noted<br>*Give P values as exact values whenever suitable.*
☒	☐	For Bayesian analysis, information on the choice of priors and Markov chain Monte Carlo settings
☐	☒	For hierarchical and complex designs, identification of the appropriate level for tests and full reporting of outcomes
☐	☒	Estimates of effect sizes (e.g. Cohen's *d*, Pearson's *r*), indicating how they were calculated

Our web collection on statistics for biologists contains articles on many of the points above.

Software and code

Policy information about availability of computer code

Data collection	Behavior data used the San Diego Instruments SR-Lab Startle Response System, custom-made black and gray acrylic boxes with 40x40x40 cm chambers (Valley Plastics, Sherman Oaks, CA), hand-scoring with stopwatches, and Microsoft Excel spreadsheets for data collection. MRI data was collected using a 7 Tesla (T) Biospec small animal MRI system using Paravision 5.2 software (Bruker) at the UCLA Brain Mapping Center using S116 Bruker gradients (400mT/m) with a single-channel surface coil and standard imaging sequences (rapid-relaxation-with-enhancement RARE and gradient-echo planar sequences). Data were acquired using the S116 Bruker gradients (400 mT/m) in combination with a single-channel surface coil (described above) and a 72 mm birdcage transmit coil. An initial series of scans was performed to confirm proper head position, then localized FASTMAP shimming was performed to improve field homogeneity. T2-weighted structural scans were acquired with a Rapid-Relaxation-with-Enhancement (RARE) sequence (RARE factor=8, Echo time (TE)=56ms, repetition time (TR)=6,020 ms, 4 averages, data matrix=128x128 in a field-of-view (FOV)=20× 20mm, slice thickness=0.5 mm, 14 slices, FA=90 deg, bandwidth (BW)=50 kHz). Then functional (BOLD) data were acquired using the same image geometry as the structural scans, with a one-shot, interleaved, gradient-echo echo planar imaging sequence with the following parameters: TE=19 ms, TR=2,000 ms, FA=30 degrees, BW 400 kHz and a data matrix of 90×60) in a FOV of 20mx20mm. 10 dummy scans were used to allow the T1 signal to reach steady-state prior to signal acquisition after which 450 repetitions were acquired for 15mins. For each mouse, baseline data was collected and on the following week rapamycin (5mg/kg) or vehicle control (DMSO) was injected i.p. 2 hours before they were imaged in an identical manner. Single nucleus and bulk sequencing data was collected using a Quiagen RNeasy Mini Kit (#74106), KAPA Stranded mRNA-Seq Kit (#KR0960) on NovaSeq6000 S2 platform (Illumina), For brain slice electrophysiology performed whole-cell patch clamp recordings in voltage- or current-clamp modes using a MultiClamp 700B Amplifier (Molecular Devices) and the pCLAMP 10.5 acquisition software (Molecular Devices). Immunoshitology was quantified on a Zeiss AxioImager Z1 fluorescent microscope with Apotome 2.0 and Stereoinvestigator MicroBrhightField software. Blood serum and brain tissue was collected from mice post-mortem.
Data analysis	Statistical analysis for univariate data was performed using Prism Graphpad v8. Single nucleus and bulk sequencing used HT-seq 0.6.1 to

generate read counts, the STAR sequence aligner (v2.4.0) and Cell Ranger used for sequence alignment, expression analysis used R-project and the Bioconductor package EdgeR and R package Libra.Geneset enrichment analysis used R package fgea. Blood cytokines were analysed using Luminex xMAP Mouse 320plex cytokine panel immunoassay. Brain dissection westernblots were analysed using the ChemiDox XRS+ Molecular Imager (Biorad) and Quantity One software (Biorad). MRI data were converted to nifti format and entered into a python-scripted, preprocessing pipeline to do the following steps: brain extraction, movement correction and co-registration to a study-created brain template, slice timing correction, Gaussian smoothing to 0.8mm, bandpass filtering 0.01-0.2Hz using the FSL Toolbox. Seed analysis was conducted by temporal correlation of whole brain data to mean signal extraction from regions of interest at the pre-processed subject-level data, and then at the group-level within the general linear model framework implemented by using FSL Feat, with statistical correct at 2.1z, P<0.01 cluster-based correction. Distribution of brain networks into a community architecture was evaluated by calculation of modularity using 1000 iterations of the Louvain algorithm180. Modules derived from this process were then further classified at the nodal level by computing the classification diversity and classification consistency as described previously181 as implemented under GraphVar.

Tensor-based deformation analysis: Structural RARE data were converted to nifti image format, brain extracted from extracranial tissue using FSL BET182. All T2-weighted anatomical data were used to construct a study-specific, mean deformation template (MDT) images using the ants Multivariate Template Construction script183 from Advanced Normalization Tools (ANTs, v.2.3.3)that consisted of bias field correction and a three-stage coregistration procedure of rigid, affine and non-linear deformable registrations. The raw data were then separately coregistered to the MDT template using a two-stage affine and symmetric diffeomorphic registration under ANTs. The resulting deformation field was then used to derive the Jacobian determinant that provides an indication of the local tissue expansion or atrophy compared to the MDT. All data were masked for a common space to ensure a contribution from all mice to each voxel, following which statistical group differences in voxel-based Jacobian values were computed using non-linear permutation testing implemented under FSL Randomise185 under the general linear model framework using cluster-based thresholding set at z=3.1 (P<0.001) and variance smoothing of 0.1mm. Data were then corrected for multivoxel comparison using false discovery rate correction at q=0.011. The primary outcomes for resting-state fMRI was functional connectivity changes assessed by network-based statistics and by network modularity measurements at baseline and after acute rapamycin treatment. The primary outcome for structural imaging were voxel-wise tissue volume changes (Jacobian determinant) in multiple brain regions.

For manuscripts utilizing custom algorithms or software that are central to the research but not yet described in published literature, software must be made available to editors and reviewers. We strongly encourage code deposition in a community repository (e.g. GitHub). See the Nature Portfolio guidelines for submitting code & software for further information.

Data

Policy information about availability of data

All manuscripts must include a data availability statement. This statement should provide the following information, where applicable:

- Accession codes, unique identifiers, or web links for publicly available datasets
- A description of any restrictions on data availability
- For clinical datasets or third party data, please ensure that the statement adheres to our policy

The data that support the findings of this study are available as follows. Single-nucleus RNA sequencing data have been deposited in the NCBI Gene Expression Omnibus (GEO) under these accession codes [superseries #GSE328222 with subseries GSE328221 (single cell) and subseries GSE328220 (bulk)] and are publicly available as of the date of publication. Raw and preprocessed resting-state functional MRI and structural MRI data have been deposited in [Zenodo.org] under DOI [https://doi.org/10.5281/zenodo.19491368]. Univariate blood cytokine, western blot, brain wet weight, behavioral analysis, and slice electrophysiology data in this study have been deposited in [FigShare.com] under DOI [https://doi.org/10.6084/m9.figshare.31966476]. Source data underlying all figures and supplementary figures are provided with this paper. Any additional information required to reanalyze the data reported in this paper is available from the corresponding author upon request.

Research involving human participants, their data, or biological material

Policy information about studies with human participants or human data. See also policy information about sex, gender (identity/presentation), and sexual orientation and race, ethnicity and racism.

Reporting on sex and gender	n/a
Reporting on race, ethnicity, or other socially relevant groupings	n/a
Population characteristics	n/a
Recruitment	n/a
Ethics oversight	n/a

Note that full information on the approval of the study protocol must also be provided in the manuscript.

Field-specific reporting

Please select the one below that is the best fit for your research. If you are not sure, read the appropriate sections before making your selection.

☒ Life sciences ☐ Behavioural & social sciences ☐ Ecological, evolutionary & environmental sciences

For a reference copy of the document with all sections, see nature.com/documents/nr-reporting-summary-flat.pdf

Life sciences study design

All studies must disclose on these points even when the disclosure is negative.

Sample size	Sample sizes were determined based on our own previous experiments and relevant experiments published in the literature to achieve a statistical power of 0.8 with an alpha of 0.05 to detect an effect size (Cohen's d) of 1.2. The significance and the effect size were reported for every result in the figure legends and results section. Group numbers has been reported in all figure legends and individual data points are plotted in result figures.
Data exclusions	Mice from multiple litters were randomly assigned to each group and no selection criteria were used. An equal number of male and female mice were used in each group. Mice injured due to fighting in home cages, which happened occasionally, were excluded from study due to possible confounding effects of single housing and medical treatments. No animal exclusions were necessary due to a failure to perform the behavioral tests. Because we used an outbred strain of mice (CD1) that have some genetic variability, a range of phenotype severities was expected. Therefore, outlier data and outlier animals were not removed from any experiment since these mice likely represent the possible range of outcomes in the model.
Replication	Multiple litters of maternal inflammation-exposed offspring and vehicle control-exposed offspring were generated and equal numbers of male and female offspring were randomly selected from all litters to generate each experimental group. Some mice were used as young adults and other animals from the same litters were kept for experiments on older adult mice. These experiments were performed with 52 total litters generated over several years of experimentation (see supplemental table 2). Every new litter generated was evaluated for repetitive behaviors in an open field to confirm that the MIR phenotype was reproduced in the new offspring before using them for experiments, including non-behavior experiments. No MIR litters failed to display a significant repetitive behavior phenotype in offspring, demonstrating the reproducibility of the model. Because batch variations in LPS strength/effectiveness have been reported we used the same batch of LPS, stored in small aliquots at -80 degrees C, and made up fresh in saline for injection for every MIR litter generated. See Fig. 1A for an outline of this experimental timeframe.
Randomization	Mice were randomly selected from multiple litters to form experimental groups and equal numbers of male and female mice were used in each group for all experiments. Separate cohorts of mice were used as young adult ages (P60-90) and old adult ages (P200-400) for specific experiments.
Blinding	Animal group identification was not written on cage cards so that researchers were blinded to experimental groups during data collection. Group identification was known to the investigator analyzing the data only after all data had been collected. Treatment group but not litter group was known when rapamycin or vehicle treatment was administered. However, due to the development of extreme repetitive behaviors in a third of the MIR offspring at older ages it was not possible to be fully blinded to the group for the old-adult mice. MIR mice at older ages also frequently weighed less due to constant repetitive activity compared to control mice which normally become more sedentary and increase in weight significantly with age. MIR mice at young adult ages were not readily identifiable by inspection of home cage behavior.

Reporting for specific materials, systems and methods

We require information from authors about some types of materials, experimental systems and methods used in many studies. Here, indicate whether each material, system or method listed is relevant to your study. If you are not sure if a list item applies to your research, read the appropriate section before selecting a response.

Materials & experimental systems

n/a	Involved in the study
☐	☒ Antibodies
☒	☐ Eukaryotic cell lines
☒	☐ Palaeontology and archaeology
☐	☒ Animals and other organisms
☒	☐ Clinical data
☒	☐ Dual use research of concern
☒	☐ Plants

Methods

n/a	Involved in the study
☒	☐ ChIP-seq
☒	☐ Flow cytometry
☐	☒ MRI-based neuroimaging

Antibodies

Antibodies used	For western blot: phospho-S6 (S235/236; 1:2000, Cell Signaling Technology, #4858), S6 (1:2000, Cell Signaling Technology #2217), and b-actin (1:2000, Cell Signaling Technology #4970), and for immunohistochemistry: IBA1 (1:200 Wako 019-19741), NeuN (1:200 ThermoFisher #78499), and Egr1 (1:100 Cell Signaling Technology #4153), secondary fluorescent Alexa antibodies (1:2000 red/green) from ThermoFisher
Validation	The companies, Cell Signaling Technology, Wako, and ThermoFisher, provide antibody validation at their website for the commercially available antibodies used for westernblot and fluorescent immunohistochemistry as specified.

Animals and other research organisms

Policy information about studies involving animals; ARRIVE guidelines recommended for reporting animal research, and Sex and Gender in Research

Laboratory animals	CD-1 mus musculus
Wild animals	n/a
Reporting on sex	Equal numbers of male and female offspring were used in every experimental group. The mice were sex-pooled because we did not observe any sexually dimorphic behavioral patterns in brain or behavior phenotypes as documented in our previous work with this model (Le Belle, et al., 2014) and this is consistent with previously reported outcomes in MIR models (Fatemi, et al., Cell Mol Neurobiol 22, 25–33 (2002)). Information on sex is included in the revised manuscript.
Field-collected samples	n/a
Ethics oversight	UCLA animal research committee (IACUC)

Note that full information on the approval of the study protocol must also be provided in the manuscript.

Plants

Seed stocks	n/a
Novel plant genotypes	n/a
Authentication	n/a

Magnetic resonance imaging

Experimental design

Design type	Resting state BOLD and T1-weighted anatomical scans
Design specifications	Resting state 450 repetitions (15mins) /session, 2 session per mouse
Behavioral performance measures	none

Acquisition

Imaging type(s)	functional and structural
Field strength	7 Tesla
Sequence & imaging parameters	Structural: T2-weighted structural scans were acquired with a Rapid-Relaxation-with-Enhancement (RARE) sequence (RARE factor=8, Echo time (TE)=56ms, repetition time (TR)=6,020 ms, 4 averages, data matrix=128x128 in a field-of-view (FOV)=20× 20mm, slice thickness=0.5 mm, 14 slices, FA=90 deg, bandwidth (BW)=50 kHz).<br><br>Functional: Functional (BOLD) data were acquired using the same image geometry as the structural scans, with a one-shot, interleaved, gradient-echo echo planar imaging sequence with the following parameters: TE=19 ms, TR=2,000 ms, FA=30 degrees, BW 400 kHz and a data matrix of 90×60) in a FOV of 20mx20mm. 10 dummy scans were used to allow the T1 signal to reach steady-state prior to signal acquisition after which 450 repetitions were acquired for 15mins.
Area of acquisition	14 Slices beginning by positioning the anterior field of view on the gap between the olfactory bulbs and the forebrain proper at Bregma +3.56mm.

Diffusion MRI ☐ Used ☒ Not used

Preprocessing

Preprocessing software	FSL Toolbox, ANTS Toolbox, GraphVar
Normalization	Slice-time corrected, normalized using non-linear ANTS to a mean deformation template calculated from the study.
Normalization template	Study-specific mean deformation template calculate dying ANTS software.

Noise and artifact removal	Movement corrected
Volume censoring	None

Statistical modeling & inference

Model type and settings	General linear model and network based statistics for functional. Non-parametric permutation testing of structural data with FSL randomize.
Effect(s) tested	No task. Anova used.

Specify type of analysis: ☐ Whole brain ☐ ROI-based ☒ Both

Anatomical location(s): Using an atlas (Ekham solutions)

Statistic type for inference (See Eklund et al. 2016)	Structural cluster-wise z=3.1 (P<0.001) and variance smoothing of 0.1mm and FDR q=0.01. Functional: Seed analysis: 2.1z, P<0.01 cluster-based correction. Edgewise: Network-based statistics P<0.01, 2-tailed, using non-parametric permutation testing over 1000 iterations.
Correction	FDR q=0.01, Cluster-wise error correction P<0.01

Models & analysis

n/a	Involved in the study
☐	☒ Functional and/or effective connectivity
☐	☒ Graph analysis
☒	☐ Multivariate modeling or predictive analysis

Functional and/or effective connectivity	Edgewise strength analysis by Pearson Correlations
Graph analysis	Wighted graph, modularity using the Louvain algorithm and nodal classification diversity and classification consistency values.
