# Supplementary material for: Acute rapamycin treatment reveals distinct mechanisms of dysfunction in a maternal inflammation mouse model
Source: Nat Commun. 2026 Jul 23;17:6386. doi: 10.1038/s41467-026-74958-1 (PMC13396360; doi:10.1038/s41467-026-74958-1)
Supplement: Supplementary file 3 — Transparent Peer Review file [file 41467_2026_74958_MOESM3_ESM.pdf]

nature portfolio

Peer Review File

Acute rapamycin treatment reveals novel mechanisms of dysfunction in a maternal inflammation mouse model

Corresponding Author: Dr Harley Kornblum

This file contains all reviewer reports in order by version, followed by all author rebuttals in order by version.

Version 0:

Reviewer comments:

Reviewer #1

(Remarks to the Author)

This well-written manuscript describes remarkable rescues of autism-relevant phenotypes by a single acute dose of rapamycin in young adult and old adult CD-1 mice born to dams given a low dose of the inflammatory agent lipopolysaccharide during pregnancy (MIR mice). Two hours after one intraperitoneal dose of the mTOR inhibitor rapamycin, phenotypes of MIR mice were reversed on behavioral, electrophysiological, and functional connectivity measures. In further support of an mTOR mechanism, single-cell RNA seq from sensorimotor cortex of MIR mice detected differentially expressed genes in the mTOR pathway.

The Introduction includes a scholarly overview of the literature on the mTOR pathway in autism spectrum disorder. The Discussion includes a careful interpretation of the results and their relationship to past studies. Referencing is comprehensive.

The authors are to be complimented on their attention to sensory processing, a phenotype that is often overlooked in mouse models of autism. Their modification of the traditional light/dark test for anxiety-related behaviors to include a component of paw tactile hypersensitivity to floor textures is elegant. Sequence of conducting the 5 behavioral assays proceeded appropriately from least to most stressful. Further, their studies include an interesting comparison with Plexxikon treatment to deplete brain microglia, and an insightful control experiment using RapaBlock, a brain impermeable small molecule which restricts rapamycin effects to the central nervous system.

Although most aspects of the manuscript demonstrate a high level of attention to methodological details, the behavioral components do not. Major issues are apparent:

1.Numbers of mice per treatment group appear to be far below the acceptable range for each of the 5 behavioral assays conducted. In fact, Ns are not stated within the text or in the legends for Figures 1, 3, 4, and 5. Scatter plot data points indicate Ns as low as 4, with maximum numbers appearing to be 8 per group in the bar graphs.

Mice are inherently variable in their behavioral scores. With these low Ns, just one or two unusual mice within a group could substantially skew the results. Rigor and reproducibility require Ns in the range of 12-15 per treatment group for most mouse behavioral assays.

It will be necessary to repeat the behavioral experiments with a new, separately bred cohort consisting of at least 12 mice per treatment group.

2. Sex of the subject mice is not stated. It is best to test both males and females, and then conduct statistical analyses for sex differences. Ideally, the replication experiment will include N=12-15 of each sex for each treatment.

3. Behavioral methods are referenced but only minimally described, in contrast to extensive methods descriptions for all other assays. Please add considerably more methodological details for each of the five behavioral tests. For example, which repetitive behaviors were scored, and how was testing conducted? If self-grooming and cage circling were scored in an open field apparatus as part of the open field exploratory behavior session, the arena should be extensively described in

terms of size, presence or absence of litter, prior habituation, etc. How was the arena cleaned between sessions? Did human observers score the behaviors from videos or in person? How were the scorers kept blind to treatment?

Analogous methodological details are needed for the other behavioral assays.

4. Three-chambered data are displayed on the y-axis as “ratio of interaction time (mouse:empty cup).” This derived statistic obscures the actual results. For example, higher open field activity in MIR mice is likely to directly influence social approach ratios. Please present the data in the standard format, displaying time spent (# seconds) with the novel mouse and time spent with the novel object (empty cup) as two bars for each treatment group.

Sociability is defined as more time with the novel mouse than with the novel object. Absence of sociability is defined as no significant difference between time with the novel mouse and time with the novel object. Statistical analysis should be WITHIN group only.

The 3-chambered assay for sociability is a binary, yes-or-no test which is not sensitive enough to compare scores across treatment groups. A more sensitive test such as reciprocal social interactions between two freely moving mice is needed to conduct meaningful comparisons between treatment groups.

Minor issues:

5. Why were three daily rapamycin injections administered before the 3-chambered social interaction test, while all of the other assays were conducted after a single rapamycin injection?

6. In the drug treatment methods section, please provide more information about the ages of the subject mice when rapamycin and the S6K1 inhibitor PF-4708671 inhibitor were administered. As the authors realize, it is surprising that a single dose of these drugs was able to reverse behavioral, electrophysiological, and neuroanatomical phenotypes. Further, younger ages might be predicted to be more sensitive to pharmacological interventions. Were the young adults exactly sixty days old, or were some younger? Were most of the old adults closer to 200 days, or closer to 400 days old, or distributed continuously across this wide age range?

7. In the Discussion section, please expand on the fact that clinical trials with the rapamycin analog Everolimus have been unsuccessful to date.

8. In the Acknowledgements section, please add the initials of the investigators who are the recipients of each grant listed.

Reviewer #2

(Remarks to the Author)
Manuscript ID: Nature Communications

Acute rapamycin treatment reveals novel mechanisms of behavioral, physiological, and functional dysfunction in a maternal inflammation mouse model of autism and sensory over-responsivity

Le Belle, Condro, Cepedaa, Oikonomoua, Tessemaa, Dudley, Schoenfield, Kawaguchia, Geschwind, AJ Silva, Zhange, Shokate, Harris, & Kornblum

Maternal inflammation (MIA), during early gestation in mice induces a cascade of physiological and behavioral changes that have been associated with autism spectrum disorder (ASD). Previous work has shown 1) neuroinflammation, 2) mTOR activation and 3) brain overgrowth. The authors assemble and discuss work from an earlier study along with current report, in which they report mild MIA (presumably “mild” is represented by the dose of LPS and/or the response of inflammation and/or illness in the Dams, but this was not reported in a numerical fashion or a standardized way to compare from earlier and other MIA papers, resulted in 1) chronic systemic and neuroinflammation, 2) mTOR pathway activation, 3) mild brain overgrowth followed by 4) regionally specific volumetric changes, 5) sensory processing dysregulation, and 6) social and repetitive behavior abnormalities.

This article is unique, as essentially all prior work with rapamycin as a drug intervention has been performed with chronic or semi-chronic treatment, this has been to prevent significant physical brain changes, such as tumor formation in tuber sclerosis mouse models. Moreover, the work presented here used acute rapamycin (5 mg/kg i.p. in 100% DMSO), 2 hrs prior to assessment for phenotypes, related to mTOR pathway signaling. Thus, the authors report, in their opinion, that within 2 hours, rapamycin treatment could rapidly rescue neuronal hyper-excitability, seizure susceptibility, functional network connectivity and brain community structure, and repetitive behaviors and sensory over-responsivity in adult offspring with persistent brain overgrowth. They also performed in depth -omics and identified associated alterations of the expression of several ASD-, ion channel-, and epilepsy-associated genes, in the same time frame, as the other phenotypes. Therefore, this manuscript is a tour de force in technical capabilities assembled to make quite substantial claims regarding a single injection of 5 mg/kg of rapamycin, in 100% DMSO. While the sheer number of technical abilities assembled herein is impressive and the utility of acute rapamycin using an environmentally induced in vivo model of NDD of MIA versus rare genetic NDDs, i.e., TSC2 or PTEN is intriguing; the authors to make huge conclusions that mTOR dysregulation in MIA

offspring is a key contributor, even with this single dose and single timepoint, to various levels of brain dysfunction, including neuronal excitability, altered gene expression in multiple cell types, sensory functional network connectivity, and modulation of information, can rescue of behaviors associated with ASD in adult preclinical models, but did not alter the long-term physical alterations to the brain that were observed in varying MIA models and have been observed in human developmental brains (Corcheschene et al.,). The basis of these substantial conclusions is behavior that is not directly causal, nor validated, nor performed with rigor, and reliability AND by weighing wet freshly dissected brains, which is a gross rudimentary index. The MRI work is impressive and if it was clear which brains had the elevations in cytokines and delivered which scores in behavior, the article may have been more convincing but there were way too many variables left up to the reader's interpretation.

The Behavioral Figures are not standardized, which severely affects the quality of the data. There have been many reports on how to interpret 3 chamber task data in Silverman et al., 2015 and Yang et al., 2011, CPNS, but most importantly, more recently in Silverman et al., 2022. The sample size is not large enough, sex effects are not considered, litter effects are not considered, there is no discussion of the criteria was to illustrate a "mild" effect. These can all be found in a wide variety of references from Crawley et al., many articles and Sukoff-Rizzo et al., 2016. Raw seconds for three chambers must be used over percentages bc there is no single # of seconds that = social. For e.g., if you investigate for 300 sec out of 600 sec are you social? 50% of the assay? This is not known bc there is not reliability from cohort to cohort in the number of raw seconds that an animal spends in each chamber. This is discussed at length in Bales et al., 2024, and Silverman et al., 2015. Nearly all the challenges with behavioral neuroscience and preclinical models that are highlighted in Silverman et al., GBB, 2022 are employed here, by lacking the control for sex effects, litter effects, motor abilities, In the Von Frey calling this assay something that it has not been validated to assay. In behavior, a task must be "validated" to say that a task is measuring the behavior, it in fact says it is measuring. Validation is done by lesion, pharmacology and/or genetics, there is no validation of the Von Frey assay; thus, it is an over statement to say "it is measuring X or Y, without proof or validation of such.

The authors may want to familiarize themselves with the weakness of the 3 chamber task, in Jabrain and Wagner's, beyond the 3-chamber assay.

The statements of the authors over rely on the 3 chambered assay, which should be utilized with greater caution, there are other social interaction tasks such as juvenile social dyads, the partition test and, of course, the highlighted 3-chambered approach task. However, each mouse social task is limited in its utility bc of a variety of reasons including: 1) mouse do not have a strong effect size (signal to noise ratio <15% of task time) for sociability; 2) mice are highly variable in performance on an anxiety-like /social assay, given they are highly variable on either test independently, and inconsistent across days and times of day; 3) Moreover, mice social signals are small in effect size, compared to rats.

It is disconcerting to see authors make robust "causal" statements based on an unreliable behavior that is not conserved via neural circuitry such as the 3-chamber task, over the years' scientists have pondered is a shy mouse a thing; does the dominance hierarchy affect 3 chamber performance? There are numerous confounding variables in social assays, such as motor and olfactory abilities, age of subject and stimulus mice, sex and background strain of subject and stimulus mice. Do these complications mean there are limited tests of social behavior, or does it mean the available tests are of limited value? The authors have overlooked this decade of work.
What is the authors definition of standardized? Standardized to me, means well validated and accepted across the field and being employed similarly across multiple laboratory environments. The task is discussed as sensitive and reliable test. This would have to be proven by sensitivity in adjusting or moving the signal in control mice on the task with a drug or a lesion r some type of injury that would change their social behavior, sensitive to change. This was not shown. Reliable again is like reproducible but different in that if a task is reliable, it should yield the same values (numbers) and effect sizes across laboratories. If your sensory skills are impaired, is the task still reliable and sensitive? It seems to me the Von Frey task would have a different outcome than the 3-chamber task based on this logic, and they should not be viewed as corresponding or corroborating indices.
Is sex ever considered, it doesn't appear so? Or litter effects?
There is never a mention of ARRIVE guidelines in the METHODOLOGY, for which the Editors may have rejected this paper, as some journals are moving toward confirming ARRIVE guidelines. Many missing methods for social tasks and neurodevelopmental modeling are provided as critical points in Silverman et al., Genes brain and behavior 2022.
The authors must dampen their conclusions substantially and alter their entire story. A major issue was the lack of mention of sex and/or standardization of the background strain. Behavior is highly variable to background strain, so the mixed background of one line cannot be directly compared to the congenic background of another.
It is for the vast differences in the authors' interpretation of their data versus this reviewer's interpretation of their data of the authors' appropriately and laboriously collected data that I cannot provide a positive recommendation to accept this manuscript.

Minor
• All citations regarding the mTOR work were reviews, not the original articles that showed effect sizes and doses in various models
• There is no mention of how and/or they chose their dose, they chose CD1 as the background strain, and there is no discussion n 100% DMSO as a vehicle despite work that has shown DMSO to adversely affect behavior.
• With the low sample size, there hardly looks to be a difference between the PLEX treated mice versus the vehicle in the older group, one would especially want to see the critical control point that as mice age, they move less but this is not captured in these figures as they are.
• The mice presented in behavior are not correlated to the level of inflammation in the blood or brain, so again, causal

statements are very much overstated.

Reviewer #3

(Remarks to the Author)
The work by LeBelle et al investigates the molecular, morphological and physiological effects of Maternal Inflammatory Response and the mTOR pathway on the onset and progression of symptoms of the autism spectrum disorder. The team has a consolidated experience on the subject, having shown that induction of inflammatory response in the dams produces the appearance of autistic phenotype overtime in the offspring, and that the effects are mitigated by rapamycin. In this work they focus on quantifying the acute effects of rapamycin in order to uncover the early mechanisms that will lead to a mitigation of the MIR. They succeed in their task using a variety of techniques, from single-cell and bulk analysis of transcripts, to behavioural essay, electrophysiology and imaging, and they identify the rebalance of excitation and inhibition as the candidate driver of rapamycin's action, although the scRNAseq data do not explicitly go in this direction.
This work is relevant because measuring the acute effect can indeed provide some hints do develop new potential treatments to improve the symptoms of these disorders, and the rationale and performance of the experiments is sound.
There are two points that the authors need to address before the manuscript can be published.
1) Genetic background of the animals studied. The authors perform their experiments on CD42 mice, while most of the animal models for ASD and related disorders (including those they mention: Mecp2 and Fmr1 mutant mice) have a bl6 or C57 background. Since the genetic background can indeed influence the molecular mechanisms during development and progression of symptoms, the authors should discuss the application of their findings in this context.
2) Statistical Analysis. The description of statistical analysis is vague. It would help to have the number of animals used clearly stated in the methods, the results, and the figures. Also, the data need to be distributed normally, and the test to be chosen adequate to the number.

Version 1:

Reviewer comments:

Reviewer #1

(Remarks to the Author)

All of the previous concerns have been admirably addressed. The authors are to be congratulated on increasing the Ns, adding a new behavioral experiment evaluating reciprocal social interactions, including more complete descriptions of behavioral methods, stating full statistical results in the figure legends, and mentioning negative results of Everolimus clinical trials. No further changes are required in this excellent manuscript which represents an impressive set of comprehensive studies.

It is recommended that the Supplementary Methods paragraph at the end of the manuscript (lines 2582-2591), describing the new reciprocal social interaction test, be moved up to the main Methods section describing the other behavioral tests (after line 806), and that the reference in this paragraph be inserted into the main reference list. This modification is at the discretion of the Editor.

Reviewer #2

(Remarks to the Author)
The authors substantial additions and revisions are a clear sign that they understood the rveiewer's comments and made every attempt possible to add clarity, details and justify why they did what they did in the original draft.

This is a well revised acceptable version.

A nice small, minor revision, might be to mentioned they added mice for sufficiant power and that they performed statistical analysis illustrating no differences in the WT vehicle across cohorts, which provides them justification to combine smaller groups of mice together for one larger cohort for reporting. with thsi smaller addition, the atypical experimental design to reach power is justified sceintifically and statistically. This can be done at the proofing stage though, I do not see the need for more revisions, at this point.

Since this justification is not readily available to cite, having it outlined herein, would increase the impact of similar behavioral studies and provide a reference.

Open Access This Peer Review File is licensed under a Creative Commons Attribution 4.0 International License, which permits use, sharing, adaptation, distribution and reproduction in any medium or format, as long as you give appropriate credit to the original author(s) and the source, provide a link to the Creative Commons license, and indicate if changes were made.
In cases where reviewers are anonymous, credit should be given to 'Anonymous Referee' and the source.
The images or other third party material in this Peer Review File are included in the article's Creative Commons license, unless indicated otherwise in a credit line to the material. If material is not included in the article's Creative Commons license and your intended use is not permitted by statutory regulation or exceeds the permitted use, you will need to obtain permission directly from the copyright holder.
To view a copy of this license, visit https://creativecommons.org/licenses/by/4.0/

We sincerely thank the reviewers and editors for their careful evaluation of our manuscript (NCOMMS-24-44298A-Z) and for the thoughtful, detailed critiques that have strengthened the work. In response, we have substantially revised the manuscript to improve rigor, clarity, and transparency. Specifically, we have expanded and standardized the behavioral methods in accordance with ARRIVE guidelines; added detailed reporting of group sizes, effect sizes (Cohen's d), degrees of freedom, and statistical approaches to all figure legends; clarified cohort structure and litter contributions with a new supplemental table; incorporated additional animals in select behavioral analyses; and provided expanded justification for strain, drug dosing, vehicle, and age parameters. Importantly, we have removed the three-chamber sociability assay and added a reciprocal social interaction test in a new cohort, refined language to avoid overstatement of causality, and expanded the Discussion to address clinical everolimus trials and limitations of the study design. We believe these revisions directly address the major concerns raised and substantially strengthen the manuscript, and we respectfully submit it for reconsideration. We have responded to each comment below. Our responses are in blue text after each comment.

Reviewers' comments:

Reviewer #1 (Remarks to the Author):

This well-written manuscript describes remarkable rescues of autism-relevant phenotypes by a single acute dose of rapamycin in young adult and old adult CD-1 mice born to dams given a low dose of the inflammatory agent lipopolysaccharide during pregnancy (MIR mice). Two hours after one intraperitoneal dose of the mTOR inhibitor rapamycin, phenotypes of MIR mice were reversed on behavioral, electrophysiological, and functional connectivity measures. In further support of an mTOR mechanism, single-cell RNA seq from sensorimotor cortex of MIR mice detected differentially expressed genes in the mTOR pathway.

The Introduction includes a scholarly overview of the literature on the mTOR pathway in autism spectrum disorder. The Discussion includes a careful interpretation of the results and their relationship to past studies. Referencing is comprehensive.

The authors are to be complimented on their attention to sensory processing, a phenotype that is often overlooked in mouse models of autism. Their modification of the traditional light/dark test for anxiety-related behaviors to include a component of paw tactile hypersensitivity to floor textures is elegant. Sequence of conducting the 5 behavioral assays proceeded appropriately from least to most stressful. Further, their studies include an interesting comparison with Plexxikon treatment to deplete brain microglia, and an insightful control experiment using RapaBlock, a brain impermeable small molecule which restricts rapamycin effects to the central nervous system.

Although most aspects of the manuscript demonstrate a high level of attention to methodological details, the behavioral components do not. Major issues are apparent:

Numbers of mice per treatment group appear to be far below the acceptable range for each of the 5 behavioral assays conducted. In fact, Ns are not stated within the text or in the legends for Figures 1, 3, 4, and 5. Scatter plot data points indicate Ns as low as 4, with maximum numbers appearing to be 8 per group in the bar graphs.

1. Response: We agree that we did not include enough detail in the original manuscript. N-number information and Cohen's d calculation of effect size has been added to the figure legends for every experiment/technique and the appropriate selection of animal number is now directly addressed in the methods section. For all significant findings we can demonstrate that our experiments were appropriately powered and have large effect

sizes. When adding some new experiments suggested by the reviewers to the manuscript (see reciprocal social interaction data in supplemental figure 1B and Apocynin treatment in figure 5D) we took the opportunity to increase the animal number for some behavior experiments using these animals and n-numbers can be seen in the figure legends (figure 3B, 5A-B). We also updated the graphing of some data where individual data points were overlapping in the bar charts in the original submission, making the animal number look smaller, so that you can now see every data point in a box and violin plot (see Fig 1-5).

Mice are inherently variable in their behavioral scores. With these low Ns, just one or two unusual mice within a group could substantially skew the results. Rigor and reproducibility require Ns in the range of 12-15 per treatment group for most mouse behavioral assays.

2. Response: We have provided more information on effect size (Cohen d) and degrees of freedom for every experiment/figure in the figure legends. We can demonstrate that our experiments were appropriately powered because the effect sizes for all significant findings were all large to very large. In some cases, more animals have now been added to behavior experiments (see response 1). Although it is not appropriate to publish group size calculations performed after the data has been collected we did these calculations for all of our significant findings for confirmation and they showed us that the groups we have used are an adequate size based on the results (statistical power of 0.8 with an alpha of 0.05 to detect and effect size of 1.2).

It will be necessary to repeat the behavioral experiments with a new, separately bred cohort consisting of at least 12 mice per treatment group.

3. Response: We apologize for not making it clear that the experiments in this manuscript were completed over the course of several years and involved 52 separately bred cohorts of MIR and vehicle-control offspring. To clarify this, we have added this information to the methods and we have also added supplemental table 2, which catalogues the number of litters used and the experiments/figures in which each was used. In our previous publication of this mild MIR model (Le Belle, et al., 2014) we report our comparison of different litters did not indicate significant litter effects and we also tested cross-fostering of litters of MIR offspring to control dams which also did not produce any significant litter effects. The core behavioral phenotypes that we have observed in this model have been replicated in 26 MIR litters used in this manuscript which demonstrates a robust reproducibility.

In addition, whenever a new cohort of offspring were generated for experiments from new pregnant dams, we performed a confirmatory behavioral test (repetitive behaviors in an open field) on each new litter to establish that the offspring had the expected behavioral phenotype as demonstrated by previous cohorts of the mice. This confirmatory data was previously not included in the repetitive behavior data presented in the manuscript for the mice that were generated and used for other experimental outcomes besides the behavioral testing, such as electrophysiology, fMRI, etc. To further address concerns about animal numbers used, we have now added repetitive behavior data from an additional 24 mice to figure 3C, which increased the number of animals but did not change the result (significantly more repetitive behaviors in MIR offspring that are significantly reduced by acute rapamycin treatment).

Sex of the subject mice is not stated. It is best to test both males and females, and then conduct statistical analyses for sex differences. Ideally, the replication experiment will include N=12-15 of each sex for each treatment.

4. Response: Equal numbers of male and female offspring were used in every experimental group. The mice were sex-pooled because we did not observe any sexually dimorphic behavioral patterns in brain or behavior phenotypes as documented in our previous work with this model (Le Belle, et al., 2014) and this is consistent with previously reported outcomes in MIR models (Fatemi, et al., Cell Mol Neurobiol 22, 25–33 (2002)). Information on sex is included in the revised manuscript.

Behavioral methods are referenced but only minimally described, in contrast to extensive methods descriptions for all other assays. Please add considerably more methodological details for each of the five behavioral tests. For example, which repetitive behaviors were scored, and how was testing conducted? If self-grooming and cage circling were scored in an open field apparatus as part of the open field exploratory behavior session, the arena should be extensively described in terms of size, presence or absence of litter, prior habituation, etc. How was the arena cleaned between sessions? Did human observers score the behaviors from videos or in person? How were the scorers kept blind to treatment?

5. Response: This brevity was due to space limitations. However, we agree with the reviewer that greater detail is important for replication. Therefore, we have included a very detailed description of these behavioral methods in the supplemental methods section, addressing all of the requested additional information above. We also include all information from the ARRIVE guidelines in the methods section and supplemental methods sections of the manuscript. In response to these specific questions: Mice were housed 4 per cage on a 12-hour light/dark cycle with ad libitum access to food and water. All behavioral experiments were performed during the light phase (10:00-16:00) by a blinded experimenter. Animals were habituated to handling for 3 days prior to experiments and habituated to the testing room for 60 minutes the day before and the day of any test. Mice which were habituated to the testing apparatus in a 10-minute habituation session on two consecutive days before testing. As part of a battery of tests performed on the same cohort of mice the test was administered in order of least to most stressful, with at least 48 hours between tests with the open field test being the first and least stressful test. Behavioral equipment was cleaned between animals with 70% ethanol followed by tap water and then dried to remove residual odor cues. Measurement of the cumulative time spent performing repetitive behaviors in an open field chamber was performed during a 10-minute session according to the methods of Peñagarikano et al. The total amount of time that the mice spent performing repetitive grooming while stationary or while moving on all body regions (and repetitive circling seen in some of the older mice) was measured. The primary outcome measure was the percentage of total testing time spent performing repetitive behaviors of any kind. A square open-field arena (40 × 40 x 40 cm) constructed of opaque gray PVC was used. The floor was non-textured and non-absorbent and no bedding was present. Illumination at the arena was set to 50 lux during testing. Human observers scored the repetitive behaviors in person with a stop watch and timer. These details are now included in the methods sections.

Analogous methodological details are needed for the other behavioral assays.

6. Response: We have expanded on this detail for all behavioral assays and handling, habituation, and housing protocols in the supplemental methods section.

Three-chambered data are displayed on the y-axis as “ratio of interaction time (mouse:empty cup).” This derived statistic obscures the actual results. For example, higher open field activity in MIR mice is likely to directly influence social approach ratios. Please present the data in the standard format, displaying time spent (# seconds) with the novel mouse and time spent with the novel object (empty cup) as two bars for each treatment group.

7. Response: Due to space limitations in the main manuscript we had presented this data as the ratio of mouse/object. However, we have previously published the 3-chamber social test in this same mild MIR model which included all of the further testing information requested by the reviewer (Le Belle, et al., 2014). In response to reviewer 2, who has very strong objections to the 3-chamber test as a valid measure of sociability in mice in general, we have removed it from this manuscript and instead performed the reciprocal social

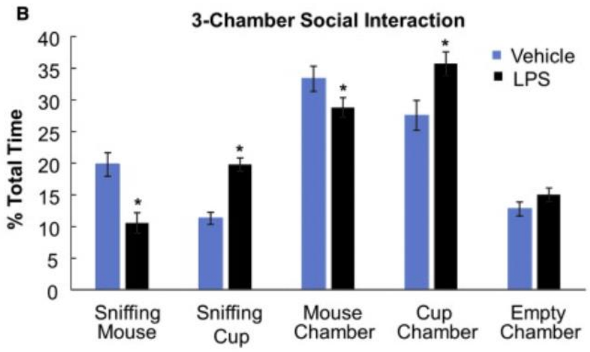

interaction test (supplemental figure 1B) which confirms a social deficit in this mild MIR model has been previously published. The 3-chamber social test has been previously published for this MIR model which included the time spent sniffing the novel mouse and novel object and the time spent in the social, middle (empty), and object chambers (Le Belle, et al., 2014). See below:

However, this test is no longer included in the current manuscript.

Sociability is defined as more time with the novel mouse than with the novel object. Absence of sociability is defined as no significant difference between time with the novel mouse and time with the novel object. Statistical analysis should be WITHIN group only.

8. Response: Thank you for your guidance on this statistical issue. This data has been removed (see response 7).

The 3-chambered assay for sociability is a binary, yes-or-no test which is not sensitive enough to compare scores across treatment groups. A more sensitive test such as reciprocal social interactions between two freely moving mice is needed to conduct meaningful comparisons between treatment groups.

9. Response: We included the 3-chamber sociability test in this manuscript due to our previous understanding of its wide acceptance as a useful pre-clinical behavioral test of social preference in rodent models. As a result of the limitations of the 3-chamber test detailed by this reviewer and reviewer 2 we have now removed the 3-chamber social data from this manuscript. However, we agree with both reviewers that there are other useful and informative social behavioral tests that can add to our understanding of social behavior in our mild MIR model, including reciprocal interactions, as suggested by the reviewer, with a novel mouse in a home cage environment. Therefore, we performed the reciprocal social interaction experiment on juvenile (P25) MIR and Control offspring with and without acute rapamycin and RapaBlock treatment according to the methods of Silverman et al., 2010 (Behavioral Phenotyping Assays for Mouse Models of Autism, Nat Rev Neurosci 11(7):490-502). This data and the detailed methodology of the experiment has been added to supplemental methods. The results in supplemental figure 1B show deficits in social behaviors and increases in self-grooming in MIR offspring compared to control offspring that is improved by acute rapamycin treatment and not affected by co-administration of RapaBlock. This new social data is in agreement with our previously published deficits in the 3-chamber test (Le Belle, et al., 2014).

Minor issues:

Why were three daily rapamycin injections administered before the 3-chambered social interaction test, while all of the other assays were conducted after a single rapamycin injection?

10. Response: We have removed the 3-chamber test from the manuscript for the reasons discussed above (response 7 & 9). The reason for the 3 daily injections previously was based on the use of rapamycin in this particular behavioral test by our collaborators in the Alcino Silva lab which also rescued behaviors in adult TSC mutant mice. They used a single repeated treatment daily for 3 days and this prior work was also the basis of our rapamycin dosage (5mg/kg). In the reciprocal social interaction test presented in the revised manuscript, we used a single injection, consistent with the rest of experiments in the manuscript.

In the drug treatment methods section, please provide more information about the ages of the subject mice when rapamycin and the S6K1 inhibitor PF-4708671 inhibitor were administered. As the authors realize, it is surprising that a single dose of these drugs was able to reverse behavioral, electrophysiological, and neuroanatomical phenotypes. Further, younger ages might be predicted to be more sensitive to pharmacological interventions. Were the young adults exactly sixty days old, or were some younger? Were most of the old adults closer to 200 days, or closer to 400 days old, or distributed continuously across this wide age range?

11. Response: The reviewer raises an interesting point regarding animal ages. Furthermore, we, too, were surprised that the effects of any of the agents were so acute. This led us to our hypothesizing that the effects of mTOR inhibition are regulating neural activity sub-acutely rather through elaboration or inhibition of anatomical functions. These findings are consistent with prior studies demonstrating that acute rapamycin can regulate the expression of proteins, such as ion channels that can then regulate neuronal function. To directly address the reviewer's questions, we have included more detailed information about ages of mice for each treatment/experiment in the figure legend describing the data for that specific experiment. Generally, the treatments of the mice with all of the pharmaceutical drugs was more effective in young adult MIR mice compared to the old adult mice, many of which had developed more extreme repetitive behaviors at older ages. Data on old adult and young adult mice were put in the same figures so that the effect of age can be easily visualized.

In the Discussion section, please expand on the fact that clinical trials with the rapamycin analog Everolimus have been unsuccessful to date.

12. Response: The original manuscript had addressed this briefly but we have added more detail about this in the discussion: "Rapid reduction of this neuronal hyper-excitability and seizure susceptibility with rapamycin treatment within the same acute time-frame as behavioral rescue suggests that restoring excitatory-inhibitory regulation in the brain may underlie the normalization of sensory dysfunction and ASD-related behaviors in the MIR model. In support of this, others have shown that an acute 2-hour pre-treatment with the rapamycin analog everolimus also reduces seizure susceptibility and suppressed neuro-inflammatory responses in a kainic acid rat epilepsy model[267]. A recent clinical trial (EXIST-3) of everolimus for epilepsy in patients with tuberous sclerosis complex (TSC) found moderate success with chronic treatment improving seizure status in around 60% of subjects that lasted at least 48 weeks[268]. However, the clinical evidence to date for everolimus effectiveness for treating other autism phenotypes like neurocognitive function or social behavioral skills is mixed with some studies finding no positive effects and others finding small improvements in autism symptoms[138,139,269]." We hypothesize that some of the failures of chronic treatment are due to the development of resistance

mechanisms. Our findings of tachyphylaxis in MIR mice with daily rapamycin treatment over 5 weeks suggests that the lack of consistent or significant effects in clinical everolimus treatment that have been reported could be related to this if a similar habituation were to take place in humans. Our preclinical approach in this manuscript was to use the acute rapamycin effects to identify novel mechanisms that could lead to the development of treatments beyond rapamycin/everolimus. Our data suggest that other drugs aimed at restoring excitatory/inhibitory balance or that address chronic systemic or brain inflammation could be effective treatments for several ASD phenotypes. We additionally identify novel targets for neuromodulation of brain sensory circuitry or brain modularity in information flow with TMS or focused ultrasound interventions.

In the Acknowledgements section, please add the initials of the investigators who are the recipients of each grant listed.

13. Response: This has been added.

Reviewer #2 (Remarks to the Author):

Maternal inflammation (MIA), during early gestation in mice induces a cascade of physiological and behavioral changes that have been associated with autism spectrum disorder (ASD). Previous work has shown 1) neuroinflammation, 2) mTOR activation and 3) brain overgrowth. The authors assemble and discuss work from an earlier study along with current report, in which they report mild MIA (presumably “mild” is represented by the dose of LPS and/or the response of inflammation and/or illness in the Dams, but this was not reported in a numerical fashion or a standardized way to compare from earlier and other MIA papers, resulted in 1) chronic systemic and neuroinflammation, 2) mTOR pathway activation, 3) mild brain overgrowth followed by 4) regionally specific volumetric changes, 5) sensory processing dysregulation, and 6) social and repetitive behavior abnormalities.

This article is unique, as essentially all prior work with rapamycin as a drug intervention has been performed with chronic or semi-chronic treatment, this has been to prevent significant physical brain changes, such as tumor formation in tuber sclerosis mouse models. Moreover, the work presented here used acute rapamycin (5 mg/kg i.p. in 100% DMSO), 2 hrs prior to assessment for phenotypes, related to mTOR pathway signaling. Thus, the authors report, in their opinion, that within 2 hours, rapamycin treatment could rapidly rescue neuronal hyper-excitability, seizure susceptibility, functional network connectivity and brain community structure, and repetitive behaviors and sensory over-responsivity in adult offspring with persistent brain overgrowth. They also performed in depth -omics and identified associated alterations of the expression of several ASD-, ion channel-, and epilepsy-associated genes, in the same time frame, as the other phenotypes. Therefore, this manuscript is a tour de force in technical capabilities assembled to make quite substantial claims regarding a single injection of 5 mg/kg of rapamycin, in 100% DMSO. While the sheer number of technical abilities assembled herein is impressive and the utility of acute rapamycin using an environmentally induced in vivo model of NDD of MIA versus rare genetic NDDs, i.e., TSC2 or PTEN is intriguing; the authors to make huge conclusions that mTOR dysregulation in MIA offspring is a key contributor, even with this single dose and single timepoint, to various levels of brain dysfunction, including neuronal excitability, altered gene expression in multiple cell types, sensory functional network connectivity, and modulation of information, can rescue of behaviors associated with ASD in adult preclinical models, but did not alter the long-term physical alterations to the brain that were observed in varying MIA models and have been observed in human developmental brains (Corcheschene et al.,).

The basis of these substantial conclusions is behavior that is not directly causal, nor validated, nor performed with rigor, and reliability AND by weighing wet freshly dissected brains, which is a gross rudimentary index.

14. We thank the reviewer. We believe that most of the concerns are related to our failure to adequately describe our results, rather than a lack of rigor in our methodology. We acknowledge, however, that we make observations that are correlated with other findings in the paper and may not be causal in the ultimate phenotype. We feel that the information that we do provide is important, however, and that it will serve to inform the community. With respect to the specific concerns, brain wet weights were only one measure of brain overgrowth performed in this paper and in our previously published paper on this model (Le Belle, et al., 2014). In Figure 2B-C we demonstrate with volumetric MRI measurements that adult MIR offspring have enlargement of some brain regions and reduced size in others. It is unfortunately not in our budget to perform volumetric MRI from birth through late adulthood. Taken together, our brain wet weight and adult MRI volumetric data support the finding of mild brain overgrowth in this model. Other labs have also produced and published mild brain overgrowth following mild maternal inflammation (Fatemi, et al., Cell Mol Neurobiol 22, 25–33 (2002)).

The MRI work is impressive and if it was clear which brains had the elevations in cytokines and delivered which scores in behavior, the article may have been more convincing but there were way too many variables left up to the reader's interpretation.

15. The large range of experimental methodologies used in our manuscript to determine some of the important mechanistic underpinnings of the effect of acute rapamycin in our MIR model required the assembly of a large, multi-disciplinary team of collaborators in several different labs (behavior, electrophysiology, MRI, sequencing/bioinformatics, etc.). Therefore, it was not practically possible to time all of the work so that all of these different measures were performed on the same animal, especially given the progression of phenotypes that we have documented with age. We agree that if this were possible it would allow greater power for statistical comparisons and would be the ideal protocol. However, for practical, real-world reasons this was not possible. Despite this, the data show significant effects in different cohorts of mice from different litters generated at different times. We believe that this demonstrates the robustness of the MIR effect on offspring which consistently have the same abnormal phenotypes. The fact that the experiments were performed on different cohorts of mice has been clarified further in the manuscript so that readers can appropriately interpret the significance of the findings from these cohorts. A chart detailing the litters and which experiments each contributed to has been added in supplemental table 2.

The Behavioral Figures are not standardized, which severely affects the quality of the data. There have been many reports on how to interpret 3 chamber task data in Silverman et al., 2015 and Yang et al., 2011, CPNS, but most importantly, more recently in **Silverman et al., 2022.**

16. Response: As mentioned in the response to reviewer 1 above (response number 7), the 3-chamber test was chosen because it has been a widely used pre-clinical behavioral test for social preference that has shown deficits in a large range of both genetic and acquired murine models of autism-associated phenotypes. However, given the recent criticisms and the questions raised about the 3-chamber test's ability to measure something like sociability in mice raised by the reviewer and in Silverman et al., 2022, we have removed this data and have performed the reciprocal social interaction test as described in Silverman et al., 2010, Behavioral Phenotyping Assays for Mouse Models of Autism, Nat Rev, Neurosci, 11(7):490-502 with juvenile MIR and control offspring from a new cohort of mice. This new data and the detailed methodology of the experiment has been added to supplemental figure 1B and supplemental methods. Although the MIR mice also show a social deficit in this additional behavioral test, the social behaviors of these mice were not a major focus of this manuscript. Therefore, we have added the new reciprocal social interaction data to supplemental data.

The sample size is not large enough, sex effects are not considered, litter effects are not considered, there is no discussion of the criteria was to illustrate a “mild” effect. These can all be found in a wide variety of references from Crawley et al., many articles and Sukoff-Rizzo et al., 2016.

17. Response: Please see our detailed response to reviewer 1 about this issue in responses number 1-3 above. In short, we have performed effect size and other analyses on all of our data and demonstrated that our experiments have been appropriately powered as demonstrated by their large to very large effect sizes. We have added the Cohen’s d effect size and degrees of freedom to the figure legend for all experiments. We have added animals to some behavioral experiments from the new cohort of mice generated for the new social testing. Please see our detailed response about this in reply number 3 above. These additional animals also demonstrate significant MIR dysfunction in these tests and did not alter our results or conclusions when added to the data sets. Our previous published studies with this MIR model did not display significant litter or sex effects (Le Belle et al., 2014). We used equal numbers of male and female offspring in each group in the current manuscript. This maternal inflammation model is considered “mild” both because a low LPS dose was chosen that produces a cytokine response but not overt sickness behavior in the pregnant dam and because the amount of brain overgrowth in the MIR offspring was mild (15-20% greater than vehicle control offspring but not macrocephalic) as we have previous published (Le Belle, et al., 2014).

Raw seconds for three chambers must be used over percentages bc there is no single # of seconds that = social. For e.g., if you investigate for 300 sec out of 600 sec are you social? 50% of the assay? This is not known bc there is not reliability from cohort to cohort in the number of raw seconds that an animal spends in each chamber. This is discussed at length in Bales et al., 2024, and Silverman et al., 2015.

18. We agree with the reviewers 1 & 2 that a fuller data set including seconds in each chamber rather than using percentages and the ratio of time spent is a thorough way to present this data. We have previously published this data on this MIR model with time spent in each chamber (Le Belle, et al., 2014). Please see response 7 above wherewe have presented this data. However, for multiple reasons described by the reviewer we are removing the 3-chamber data from the manuscript. We have now added a different social test, the reciprocal social interaction experiment on juvenile (P25) MIR and Control offspring with and without acute rapamycin and RapaBlock treatment according to the methods of Silverman et al., 2010 (Behavioral Phenotyping Assays for Mouse Models of Autism, Nat Rev Neurosci 11(7):490-502). See supplemental figure S1 and supplemental methods.

Nearly all the challenges with behavioral neuroscience and preclinical models that are highlighted in Silverman et al., GBB, 2022 are employed here, by lacking the control for sex effects, litter effects, motor abilities, In the Von Frey calling this assay something that it has not been validated to assay. In behavior, a task must be “validated” to say that a task is measuring the behavior, it in fact says it is measuring. Validation is done by lesion, pharmacology and/or genetics, there is no validation of the Von Frey assay; thus, it is an over statement to say “it is measuring X or Y, without proof or validation of such.

19. We appreciate the reviewer’s criticism and will change the wording in the manuscript to reflect the fact that the Von Frey test results show a lower fiber force strength threshold for limb withdrawal in MIR offspring rather than saying that it shows greater sensory sensitivity. However, taken together the multiple sensory-related tests that we performed indicate that the MIR mice show abnormal sensory processing which is supported by the functional brain imaging findings in MIR mice. Relatedly, rapamycin pharmacologically improves all of the sensory-related behaviors.

The authors may want to familiarize themselves with the weakness of the 3 chamber task, in Jabrain and Wagner's, beyond the 3-chamber assay. The statements of the authors over rely on the 3 chambered assay, which should be utilized with greater caution, there are other social interaction tasks such as juvenile social dyads, the partition test and, of course, the highlighted 3-chambered approach task. However, each mouse social task is limited in its utility bc of a variety of reasons including: 1) mouse do not have a strong effect size (signal to noise ratio <15% of task time) for sociability; 2) mice are highly variable in performance on an anxiety-like /social assay, given they are highly variable on either test independently, and inconsistent across days and times of day; 3) Moreover, mice social signals are small in effect size, compared to rats.

20. Response: The 3-chamber social test was one of a large battery of tests performed on the mice and was not a major focus of the paper. However, we appreciate the concerns of the reviewer and have re-assessed our approach for the reasons described above. Therefore, as stated above, we have removed the 3-chamber test from the manuscript and added an additional reciprocal interaction social test to supplemental data.

It is disconcerting to see authors make robust "causal" statements based on an unreliable behavior that is not conserved via neural circuitry such as the 3-chamber task, over the years' scientists have pondered is a shy mouse a thing; does the dominance hierarchy affect 3 chamber performance? There are numerous confounding variables in social assays, such as motor and olfactory abilities, age of subject and stimulus mice, sex and background strain of subject and stimulus mice. Do these complications mean there are limited tests of social behavior, or does it mean the available tests are of limited value? The authors have overlooked this decade of work.

21. Response: The 3-chamber test was one of many behavioral tests performed in this manuscript. The reviewer makes a strong case for social behaviors being difficult to measure given all of the confounds that have been mentioned. For this reason, we are taking the 3-chamber data out of the manuscript. We have updated the manuscript results and discussion to make certain that we do not make any causal claims about social behaviors.

What is the authors definition of standardized? Standardized to me, means well validated and accepted across the field and being employed similarly across multiple laboratory environments. The task is discussed as sensitive and reliable test. This would have to be proven by sensitivity in adjusting or moving the signal in control mice on the task with a drug or a lesion r some type of injury that would change their social behavior, sensitive to change. This was not shown. Reliable again is like reproducible but different in that if a task is reliable, it should yield the same values (numbers) and effect sizes across laboratories. If your sensory skills are impaired, is the task still reliable and sensitive? It seems to me the Von Frey task would have a different outcome than the 3-chamber task based on this logic, and they should not be viewed as corresponding or corroborating indices.

22. Reply: As discussed above, we have removed all of the 3-chamber social data and discussion of it out of the paper. It is beyond the scope of this work to do lesion studies on top of trying to understand the neurodevelopmental changes in the MIR brain. We do use a drug in these studies that changes social behavior, rapamycin. The focus of this paper is identifying novel mechanisms for the rapid effects of acute rapamycin treatment on a large range of behavioral and physiological phenotypes present in the MIR model. In multiple tests we see that MIR offspring have increased sensory sensitivity/reactivity and we do not observe any indication of decreased sensory perception. We interpret our findings to mean that there are sensory sensitivity and processing abnormalities that have been seen with similar effect sizes in other labs with the genetic autism FMR1 and Mecp2 mouse models.

Is sex ever considered, it doesn't appear so? Or litter effects?

23. Please see response to reviewer 1 (response number 3 & 4).

There is never a mention of ARRIVE guidelines in the METHODOLOGY, for which the Editors may have rejected this paper, as some journals are moving toward confirming ARRIVE guidelines. Many missing methods for social tasks and neurodevelopmental modeling are provided as critical points in Silverman et al., Genes brain and behavior 2022.

24. We agree with the reviewer that the ARRIVE guidelines are an important approach for reporting behavioral methods and appreciate being alerted to this. We have now fully addressed the ARRIVE guidelines in the methods and supplemental methods section of the manuscript.

The authors must dampen their conclusions substantially and alter their entire story. A major issue was the lack of mention of sex and/or standardization of the background strain. Behavior is highly variable to background strain, so the mixed background of one line cannot be directly compared to the congenic background of another.

25. All of the MIR offspring and (vehicle) control offspring used in the experiments in this manuscript were the exact same CD-1 strain of mice. The MIR model is an acquired model, not a genetic model. Therefore, no mixed background strains were used. CD-1 mice are an outbred strain which is more clinically relevant for studying the effects of maternal inflammation. Using an outbred strain is a strength of the mild MIR model as it more closely resembles the human condition and produces offspring with different severities of the same phenotypes as is seen clinically. Further information about the strain (CD-1) has been added to the methodology. We agree that we can't generalize our studies across all mouse strains. We discuss in the manuscript that this model developed in the CD-1 strain is unusual in that it has a larger than normal array of autism-relevant phenotypes that may not be replicated using another strain. We have worked with CD-1 strain for years for our studies on the cellular mechanisms of brain overgrowth (Le Belle, et al., 2011) because CD-1 neural stem and progenitor cell cultures share a similar Hif-1alpha-mediated response to low (physiological) oxygen levels with human cells by increasing stem cell self-renewal, progenitor proliferation, and neurogenesis. The ubiquitous C57BL/6 strain of mice do not appear to have this same Hif-1 alpha-redox response and therefore may not have a response to maternal inflammation as similar to humans as the CD-1 strain models.

It is for the vast differences in the authors' interpretation of their data versus this reviewer's interpretation of their data of the authors' appropriately and laboriously collected data that I cannot provide a positive recommendation to accept this manuscript.

26. The majority of the issues raised by the reviewer were specifically related to social behavior testing which has been addressed by removing the 3-chamber data and providing an additional social test that has been added to the supplemental data. Concerns about experimental and statistical detail have also been fully addressed. The ARRIVE guidelines have been used to update and improve the methods section. We hope that the reviewer can now change their recommendation.

Minor

• All citations regarding the mTOR work were reviews, not the original articles that showed effect sizes and doses in various models

27. The following <u>primary articles</u> related to mTOR have been cited: Groszer, M. et al. Proc Natl Acad Sci U S A 103, 111–116 (2006); Gregorian, C. et al J Neurosci 29, 1874–1886 (2009); Antoine, M. W., et al. Neuron 101,

648-661.e4 (2019); Kassai, H. et al. Cell Rep 7, 1626–1639 (2014); Angliker, N., Burri, M., Zaichuk, M., Fritschy, J.-M. & Rüegg, M. A. Eur J Neurosci 42, 2595–2612 (2015); McCabe, M. P. et al. Elife 9, e51440 (2020); Backman, S. A. et al.. Nat Genet 29, 396–403 (2001); Weston, M. C., Chen, H. & Swann, J. W. Front Mol Neurosci 7, 1 (2014); Kwon, C. H. et al. Nat Genet 29, 404–411 (2001); Barrows, et al. J Neurosci 37, 8595–8611 (2017); Zhao, J.-P. & Yoshii, A. Mol Brain 12, 6 (2019); Malik, R. et al. Nat Commun 10, 4994 (2019); Artinian, J. et al. J Neurosci 39, 8439–8456 (2019); Fu, C. et al. Cereb Cortex 22, 2111–2119 (2012); Haji, N. et al. Mol Autism 11, 29 (2020); Weston, M. C,. et al Neurosci 32, 11441–11452 (2012); Marchese, M. et al. BMC Med Genet 15, 26 (2014); Jansen, L. A. et al. Brain 138, 1613–1628 (2015); Koboldt, D. C. et al. Brain 144, 2971–2978 (2021); Rosina, E. et al. Transl Psychiatry 9, 1–10 (2019); Onore, C., et al. Dynamic Akt/mTOR Signaling in Children with Autism Spectrum Disorder. Front Pediatr 5, 43 (2017); Xing, X. et al. Neuropharmacology 165, 107816 (2020); Kabitzke, P. A. et al. Genes Brain Behav 17, 4–22 (2018); Canales, C. P. et al. Elife 10, e60100 (2021).

• There is no mention of how and/or they chose their dose, they chose CD1 as the background strain, and there is no discussion n 100% DMSO as a vehicle despite work that has shown DMSO to adversely affect behavior.

28. The dose and vehicle were based on previous published work from the Alcino Silva lab (Ehninger, et al., Nature Medicine 14: 843–848 (2008) and Lopez-Aranda, et al., Science Advances Vol. 7, No. 38 (2021)) and the appropriate vehicle controls were used for all drug treatments administered to animals. Further information about this has been added to the methods section. No significant effects on behavior or other physiological measures was seen from any vehicle treatment in this manuscript. We have added further explanation for choosing the CD1 strain of mice in the methods: The CD-1 strain of mice was used because of the similarity that their stem and progenitor brain cells have to human brain Hif1-alpha redox responses that are relevant to an inflammation model, their large litter sizes, and their consistent responses in behavioral testing as seen in our original publication of this MIR model (Le Belle, et al., 2014)

• With the low sample size, there hardly looks to be a difference between the PLEX treated mice versus the vehicle in the older group, one would especially want to see the critical control point that as mice age, they move less but this is not captured in these figures as they are.

29. Total distance traveled in the open field for control and MIR offspring has been added to supplemental data to demonstrate the reduced movement with age. We report and discuss in the manuscript that we did not observe a strong effect of the Plexxikon drug on the older mice, only the younger mice. We have updated the results to emphasize this more. These findings suggest that microglia may mediate the genesis of the behavioral phenotype, but are not required for the ongoing severe behavior seen in older mice.

• The mice presented in behavior are not correlated to the level of inflammation in the blood or brain, so again, causal statements are very much overstated.

30. The reviewer points out a limitation to our study. As discussed in the response to reviewer 1 (response number 15) we agree that it would have been ideal to collect all of the data from the same animal but this was not possible. We will update the manuscript to reflect this and to refrain from unwarranted causal statements. Our data from different cohorts/litters of MIR mice show the robustness of the MIR effect on offspring which consistently have the same abnormal phenotypes.

Reviewer #3 (Remarks to the Author):

The work by LeBelle et al investigates the molecular, morphological and physiological effects of Maternal Inflammatory Response and the mTOR pathway on the onset and progression of symptoms of the autism

spectrum disorder. The team has a consolidated experience on the subject, having shown that induction of inflammatory response in the dams produces the appearance of autistic phenotype overtime in the offspring, and that the effects are mitigated by rapamycin. In this work they focus on quantifying the acute effects of rapamycin in order to uncover the early mechanisms that will lead to a mitigation of the MIR. They succeed in their task using a variety of techniques, from single-cell and bulk analysis of transcripts, to behavioural essay, electrophysiology and imaging, and they identify the rebalance of excitation and inhibition as the candidate driver of rapamycin's action, although the scRNAseq data do not explicitly go in this direction.

This work is relevant because measuring the acute effect can indeed provide some hints do develop new potential treatments to improve the symptoms of these disorders, and the rationale and performance of the experiments is sound.

There are two points that the authors need to address before the manuscript can be published.

1) Genetic background of the animals studied. The authors perform their experiments on CD42 mice, while most of the animal models for ASD and related disorders (including those they mention: Mecp2 and Fmr1 mutant mice) have a bl6 or C57 background. Since the genetic background can indeed influence the molecular mechanisms during development and progression of symptoms, the authors should discuss the application of their findings in this context.

Response: We used the CD1 outbred strain of mice because its genetic variability is more relevant to human condition as reflected in the range of severity of phenotypes in the mild MIR model. This has been addressed in the methods. Maternal inflammatory response (MIR) is an acquired model of neurodevelopmental changes, not a genetic mutation study so the use of the C57BL/6 strain of mice that is a common control in studies with transgenic mutants was not needed. The goal is to produce a murine model with consistent phenotypes relevant to autism, which the CD1 mice exposed to maternal inflammation successfully provide. We produced multiple MIR litters over several years with consistent repetitive behaviors, sensory dysregulation, brain overgrowth, chronic m-TOR activation and other phenotypes relevant to autism. We have previously published this MIR model in CD1 mice (Le Belle, et al., 2014) and have extended our studies with them in this manuscript.

2) Statistical Analysis. The description of statistical analysis is vague. It would help to have the number of animals used clearly stated in the methods, the results, and the figures. Also, the data need to be distributed normally, and the test to be chosen adequate to the number.

Response: The description of group numbers, statistical analyses, and results have been updated in the manuscript to address these concerns and further information about effect sizes, statistical tests, degrees of freedom and group numbers have been added to all figure legends.
